# Exceptional Uptake, Limited Protein Expression: Liver Macrophages Lost in Translation of Synthetic mRNA

**DOI:** 10.1002/advs.202409729

**Published:** 2025-01-10

**Authors:** Cheng Lin, Adrian Kuzmanović, Nan Wang, Liangliang Liao, Sabrina Ernst, Christian Penners, Alexander Jans, Thomas Hammoor, Petra Bumnuri Stach, Mona Peltzer, Ines Volkert, Elisabeth Zechendorf, Reham Hassan, Maiju Myllys, Christian Liedtke, Andreas Herrmann, Gurudas Chakraborty, Christian Trautwein, Jan Hengstler, Gerhard Müller‐Newen, Junqing Wang, Ahmed Ghallab, Matthias Bartneck

**Affiliations:** ^1^ Department of Internal Medicine III University Hospital RWTH Aachen Pauwelsstraße 30 52074 Aachen Germany; ^2^ Department of Rheumatology and Shanghai Institute of Rheumatology Renji Hospital School of Medicine Shanghai Jiao Tong University Shanghai 200240 China; ^3^ Department of General Surgery Ruijin Hospital Shanghai Jiao Tong University School of Medicine Shanghai 200240 China; ^4^ Japan Union Hospital of Jilin University 130033 Changchun China; ^5^ Confocal Microscopy Facility Interdisciplinary Center for Clinical Research IZKF University Hospital RWTH Aachen 52074 Aachen Germany; ^6^ DWI – Leibniz Institute for Interactive Materials Forckenbeckstraße 50 52074 Aachen Germany; ^7^ Institute of Technical and Macromolecular Chemistry RWTH Aachen University Worringerweg 2 52074 Aachen Germany; ^8^ Department of Intensive and Intermediate Care University Hospital RWTH Aachen 52074 Aachen Germany; ^9^ Leibniz Research Centre for Working Environment and Human Factors 44139 Dortmund Germany; ^10^ Department of Forensic and Veterinary Toxicology Faculty of Veterinary Medicine South Valley University 83523 Qena Egypt; ^11^ Institute of Biochemistry and Molecular Biology RWTH Aachen University Pauwelsstraße 30 52074 Aachen Germany

**Keywords:** hepatocytes, lipid nanoparticles, non‐parenchymal cells, ploidy, synthetic mRNA

## Abstract

Most gene therapies exert their actions via manipulation of hepatocytes (parenchymal cells) and the reasons behind the suboptimal performance of synthetic mRNA in non‐parenchymal cells (NPC) such as Kupffer cells (KC), and liver macrophages, remain unclear. Here, the spatio‐temporal distribution of mRNA encoding *enhanced green fluorescent protein* (*Egfp*), siRNA, or both co‐encapsulated into lipid nanoparticles (LNP) in the liver in vivo using real‐time intravital imaging is investigated. Although both KC and hepatocytes demonstrate comparable high and rapid uptake of mRNA‐LNP and siRNA‐LNP in vivo, the translation of *Egfp* mRNA occurs exclusively in hepatocytes during intravital imaging. Despite attempts such as inhibiting intracellular ribonuclease, substituting uridine bases in mRNA with pseudouridine, and using a different ionizable lipid in the LNP mixture, no substantial increase in *Egfp* translation by NPC is possible. The investigation reveals that hepatocytes, which are distinct from other liver cells due to their polyploidy, exhibit significantly elevated levels of total RNA and protein, along with a higher proportion of ribosomal protein per individual cell. Consequently, fundamental cellular differences account for the low mRNA translation observed in NPC. The findings therefore suggest that cellular biology imposes a natural limitation on synthetic mRNA translation that is strongly influenced by cellular ploidy.

## Graphical abstract

Most gene therapies function via manipulating hepatocytes, while it remains difficult to modulate macrophages and other non‐parenchymal cells. Fundamental cell biology is demonstrated, but not a lack of uptake accounts for the lack of translation: hepatocytes are polyploid cells and their polyploidy enables them to contain significantly higher amounts of DNA, RNA, and proteins.

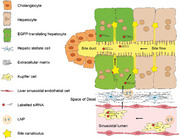

## 1 Introduction

Genetic medicines that are injected systemically are virtually only successful in treating diseases related to processes in hepatocytes, the parenchymal cells of the liver. While intramuscular injections such as mRNA vaccines effectively reach and instruct immune cells in muscle tissue, it remains a particular challenge to target hepatic non‐parenchymal cells (NPC) with mRNA upon systemic injection. Hepatocytes have various functions, they are involved in blood filtration and endocytic uptake of lipids, growth factors, and trophic agents through specific receptors.^[^ ^1^ ^]^ Interestingly, drugs based on lipid nanoparticles (LNP),^[^ ^2^ ^]^ but also other carriers that are approved for gene therapy exert their way of action via hepatocytes.^[^ ^3^ ^]^

While hepatocytes are a recognized target for genetic medicines, the introduction of mRNA to Kupffer cells (KC) could pave the way for new therapeutic strategies. This is because these cells are key NPC that process immune system‐related responses and thereby play a crucial role in regulating tolerance and anti‐tumor immunity.^[^ ^4^ ^]^ In recent years, LNP formulations were designed to increase the accumulation of synthetic RNA in hepatic NPC: one attempt included replacing the helper lipid 1,2‐distearoyl‐sn‐glycero‐3‐phosphocholine (DSPC) with 1,2‐distearoyl‐sn‐glycero‐3‐phosphoglycerol (DSPG).^[^ ^5^ ^]^ Others have reported that LNP containing piperazine‐based ionizable lipids increase absorption by NPC and, especially, immune cells.^[^ ^6^ ^]^ The successful delivery of Cre‐mRNA into different NPC, particularly KC, was proven by gene deletion in different liver cells of gene‐floxed mice.^[^ ^7^ ^]^ Thus, several studies suggested that hepatic macrophages such as KC translate synthetic mRNA at detectable levels.^[^ ^5^, ^7^, ^8^ ^]^

However, an examination of the longitudinal protein translation by NPC in real‐time remains an unmet need in the field. Here, we generated LNP loaded with labeled siRNA, *enhanced green fluorescent protein* (*Egfp*) encoding mRNA, fluorochrome‐labeled *Egfp* mRNA, or coencapsulated siRNA and mRNA. We used intravital microscopy (IVM) to study the uptake of LNP by different liver cells. IVM enables to direct record of biological events spatiotemporally at cellular and subcellular resolution.^[^ ^9^ ^]^ Hepatocytes and NPC were isolated from mice treated with LNP and studied by RNA sequencing to characterize the effects of the carriers in each cell type. We further studied the potential role of ribonucleases for a potential intracellular degradation of synthetic RNA and explored the role of mRNA modifications as well as of alternative ionizable lipids on mRNA translation. Moreover, we investigated fundamental cellular biology including the quantities of total RNA and proteins per cell. Finally, we examined the impact of cellular polyploidy on mRNA translation by hepatocytes and other cell types.

## 2 Results and Discussion

### 2.1 Exploring *Egfp mRNA* Translation Efficiency Across Liver Cell Types

We initially hypothesized that the different liver cells were similar in their translation of synthetic mRNA, based on previous studies.^[^ ^5^, ^6^, ^7^ ^]^ Thus, we encapsulated unmodified commercial *Egfp* mRNA into LNP using the lipid mixture of the approved drug Onpattro by microfluidic mixing (LNP1).^[^ ^10^ ^]^ These and all other batches of LNP generated in this study were also characterized using an optical imaging‐based method that detects the size and number of LNP and generates LNP micrographs (Figure S1, Supporting Information). We further analyzed the hydrodynamic diameter of LNP using dynamic light scattering (DLS) and assessed the zetapotential of the LNP. The average size of the LNP1 was 130 nm (**Table** **1**), which was within the expected size range of 80–130 nm based on previous studies of our group.^[^ ^2^ ^]^

**Table 1:** Physicochemical characterization of LNP with encapsulated *Egfp* mRNA. LNP were generated using microfluidic mixing. Data of at least 3 measurements ± SEM.

LNP no.	mRNA	Ionizable Lipid	Size by NS [nm]	Concentration by NS [particles/mL]	Size by DLS [nm]	Zetapotential [mV]
1	*Egfp*‐mRNA‐LNP (HS)	MC3	130.0 ± 31.9	3.60 ± 0.48E+08	103.5 ± 1.23	−4.95 ± 0.43
2	AF488‐labelled *Egfp*‐mRNA‐LNP (LS)	MC3	129.4 ± 38.4	4.63 ± 0.87E+08	92.65 ± 0.163	−6.65 ± 0.52
3	*Egfp*‐mRNA‐5moU (HS)	MC3	138.6 ± 34.1	9.24 ± 0.78E+08	105 ± 2.4	−2.81 ± 0.808
4	*Egfp*‐mRNA‐5moU (HS)	SN102	127.8 ± 29.7	5.42 ± 0.43E+08	107.7 ± 2.25	−2.74 ± 0.319
5	*Egfp*‐mRNA (HS)	SN102	135.6 ± 36.4	3.74 ± 0.33E+08	105 ± 2.4	−4.21 ± 0.878
6	*Egfp*‐mRNA‐ m1Ψ (HS)	MC3	142.2 ± 34.8	4.60 ± 0.12E+08	105.2 ± 0.793	−3.76 ± 0.998
7	*Egfp*‐mRNA‐ m1Ψ (HS)	SN102	127.5 ± 26.1	5.74 ± 0.45E+08	104.2 ± 0.3	−0.24 ± 0.756

Table abbreviations: DLS: Dynamic light scattering; NS: NanoSight; LS: low batch; HS: high batch; mV: milli Volt; 5moU: 5‐methoxyuridine; m1Ψ: N1‐Methylpseudouridine.

**Figure 1:**
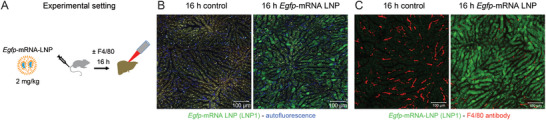
Translation of *Egfp* mRNA by liver cells in vivo. A) Experimental setup of the in vivo experiment. B) Intravital microscopy of liver 16 h after the injection of DMSO control and 16 h after the injection of DMSO and 2 mg k^−1^g *Egfp* mRNA‐LNP (LNP1) into C57BL6J wild type mice. C) Intravital microscopy done as in panel B, but with additional in vivo staining of F4/80.

We therefore studied the spatial distribution of EGFP in vivo using IVM following intravenous injection of LNP1 at the dosage of 2 mg k^−1^g into C57BL6J wild‐type mice (**Figure** **1**A). Intravital imaging was performed 16 h after LNP administration, a time point selected based on preliminary experiments. Interestingly, EGFP signals were detected only in hepatocytes (hepatocytes can be identified by their mosaic pattern). The intensity of protein translation varied between different hepatocytes; larger hepatocytes exhibited stronger signals than smaller ones. Surprisingly, no obvious signal was detected in any other hepatic cell type (Figure 1B) and in vivo staining of the KC marker F4/80 demonstrated that there were no EGFP^+^ KC. We also observed that, following LNP treatment, KC appeared smaller and more rounded compared to the F4/80 staining in control mice, where the KC in untreated mice exhibited their typical elongated morphology (Figure 1C).

### 2.2 Comparison of the uptake and translation of LNP1 by different cell types

**Figure 2:**
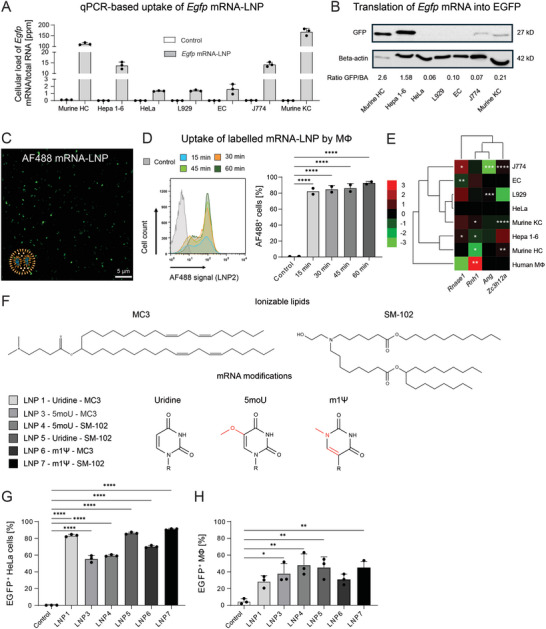
Uptake and translation of *Egfp* mRNA‐LNP by different cell types. A) Various different cell types were left untreated or were incubated with LNP1 and the uptake was assessed using quantitative Realtime‐PCR (expressed as parts per million, ppm of total RNA). B) EGFP protein translation by different cell types determined via immunoblot. C) Confocal micrograph of LNP2 (AF488‐labelled mRNA). D) Uptake of AF488‐labelled *Egfp* mRNA by macrophages assessed by flow cytometry after different time‐points. E) qPCR‐based analysis of the gene expression of selected ribonucleases on different cell types. F) Ionizable lipids and key types of modified *Egfp* mRNA in which the uridine base of unmodified mRNA was replaced with 5moU or m1Ψ used to generate LNP formulations. G) Hela cells were transfected with 1 µg mL^−1^ *Egfp* mRNA‐LNP for 24 h and then subjected to flow cytometry and compared to H) human primary macrophage with 1 µg mL^−1^ *Egfp* mRNA‐LNP. Data represent mean of *n* = 3 ± SD; *p < 0.05, **p < 0.01, ***p < 0.001, **** *p* < 0.0001 (One‐way ANOVA).

Stimulated by the surprisingly low level of EGFP‐derived signals in KC, we studied the uptake of LNP1 in vitro by incubating primary murine hepatocytes, the murine hepatoma cell line Hepa 1–6, human cervical cancer cells (HeLa), murine L929 fibroblasts, human endothelial cells (EA.hy926), murine J774.1 macrophages, and murine KC for 24 h with LNP in medium with 5% serum. We used quantitative Realtime‐PCR (qPCR) to assess the uptake of *Egfp* mRNA relative to the amount of total RNA in each cell type (details in Figure S2, TableS1, Supporting Information). We used only Mycoplasma‐free cell cultures in our study (Figure S3, Supporting Information). We observed that KC, followed by hepatocytes and the Hepa 1–6 cells, internalized the largest amounts of LNP1 (**Figure** **2**A). We confirmed the low EGFP protein translation by KC using flow cytometry (Figure S4A). Since particularly hepatocytes exhibit autofluorescence which can lead to false positive signals in the green light region that also tracks EGFP (Figure S4B, Supporting Information), we analyzed protein translation using an immunoblot. It turned out that hepatocytes, and similarly the Hepa 1–6 cells, were most efficient in EGFP protein production (Figure 2B), quantifications of the immunoblot signals revealed that these differences were statistically significant (Figure S4C, Supporting Information).

We next generated LNP2 which contained 5‐Propargylamino‐CTP‐PEG5‐AF488 labeled *Egfp* mRNA, thereby allowing the direct visualization of the mRNA. We found using confocal microscopy that the signals in LNP2 were evenly distributed over different particles (Figure 2C). Physicochemical characterizations demonstrated that the LNP2 exhibited similar properties as the LNP1 (Table 1). To further confirm the rapid uptake of LNP by macrophages, we incubated human primary macrophages with LNP2 for up to 60 min and analyzed the uptake using flow cytometry. We noted that they rapidly absorbed significant amounts of LNP2 after as few as 15 min (Figure 2D).

Knowing that the cell type‐specific uptake of LNP could not explain the lack of EGFP translation in vivo, we further studied the impact of LNP1 on the expression of selected intracellular ribonucleases and a ribonuclease inhibitor since we hypothesized that macrophages and endothelial cells (EC) might degrade the mRNA after uptake by their intracellular ribonucleases. We, therefore, studied the effects of LNP1 on the expression of selected ribonuclease genes and a ribonuclease inhibitor by the cells studied in Figure 2A and human primary macrophages using qPCR. In detail, we selected *ribonuclease 1* (*Rnase1*) as it was reported to be expressed by endothelial cells^[^ ^11^ ^]^ and macrophages,^[^ ^12^ ^]^ the *zinc finger CCCH type containing 12A* (*Zc3h12a*) as it was shown to exhibit RNase activity for certain mRNA targets,^[^ ^13^ ^]^ as well as *Angiogenin* (*Ang, Rnase5*), a gene that is activatable in macrophages.^[^ ^14^ ^]^ We also analyzed *Ribonuclease inhibitor 1* (*Rnh1*), a leucine‐rich repeat protein that degrades the RNASE1 protein.^[^ ^15^ ^]^

Interestingly, the LNP1 stimulated enhanced expression of *Rnase1* and *Zc3h12a* whereas they led to downregulated expression of *Ang* in J774.1 cells (Figure 2E). The LNP treatment evoked reduced expression of *Rnase1* in EC and caused increased expression of *Ang* in fibroblasts, whereas HeLa cells were nearly unaffected by the LNP. We detected an induction of *Rnh1* and of *Zc3h12a* in murine KC upon LNP treatment. In Hepa 1–6 cells, the LNP caused a downregulation of *Rnh1* and *Rnase1* whereas murine HC showed reduced expression of *Rnh1* but enhanced expression of *Zc3h12a* following LNP treatment. The LNP induced a significant upregulation of the *Rnh1* gene in human primary macrophages (Figure 2E).

Although statistical significance was detected for several conditions in Figure 2D, the actual changes in gene expression were very moderate, with the highest fold‐change being a six‐fold induction of *Rnh1* in human primary macrophages. Since *Rnh1* is an antagonist of *Rnase1*,^[^ ^15^ ^]^ and to study the potential impact of *Rnase1* on mRNA translation by macrophages, we used siRNA‐LNP to knock down *Rnase1* for 24 h before treating the cells with LNP1 for another 24 h. Flow cytometry showed that the EGFP signal remained unaffected by the knockdown of *Rnase1* (Figure S4D, Supporting Information).

In summary, there was no apparent induction of any ribonuclease by LNP1 which could provide an explanation for the lack of EGFP translation by KC. While earlier studies by other groups demonstrated that macrophages,^[^ ^14^ ^]^ KC,^[^ ^12^ ^]^ and LSEC^[^ ^11^ ^]^ express ribonucleases, this expression is more likely indicative of other biological functions of these proteins within these cells.

The next step included the exploration of the role of mRNA modifications and of a different ionizable lipid on mRNA translation by HeLa cells and macrophages. We namely chose SM‐102,^[^ ^16^ ^]^ to replace the ionizable lipid MC3. The COVID‐19 vaccines have employed modified mRNA with the uridine derivatives N1‐methyl‐Pseudouridine (m1Ψ)^[^ ^17^ ^]^ and 5‐methoxyuridine (5moU).^[^ ^18^ ^]^ We therefore generated and characterized 5 additional LNP formulations LNP3‐LNP7 (Table 1) which included combinations of the ionizable lipid SM‐102 and m1Ψ or 5moU modified *Egfp* mRNA (Figure 2F). We observed using flow cytometry that HeLa cells translated the mRNA of all formulations into EGFP protein at significant amounts (Figure 2G). In contrast to HeLa cells, the translation by macrophages was low, irrespective of the type of ionizable lipid or mRNA used (Figure 2H).

These findings indicate that the low rate of mRNA translation by KC and macrophages cannot be fundamentally enhanced by modifying mRNA or by employing a different ionizable lipid.

### 2.3 Longitudinal Biodistribution of LNP Containing Labelled RNA

**Figure 3:**
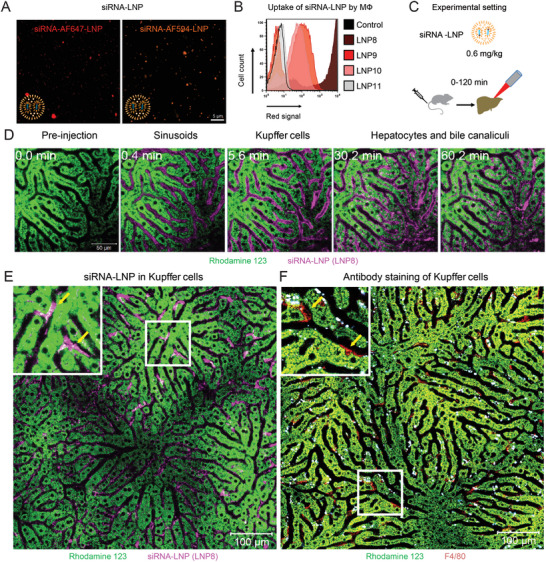
Uptake and excretion of LNP containing labeled RNA by liver cells. A) Visualization of LNP by confocal microscopy. B) Uptake of LNP containing labeled siRNA (LNP8:AF647; LNP9: AF594; LNP10: 2×594; LNP11: unlabelled; see Tables 2 and 3 for details) by human primary macrophages in vitro determined by flow cytometry (details on LNP are shown in Tables 2 and 3). C) Setup of the in vivo IVM experiment which covers 120 min after LNP injection. D) Rhodamine‐staining of HC followed by injection of LNP8 imaged by IVM at the indicated time points. E) Enlarged view of panel D and a zoom‐in on the KC with yellow arrows pointing at KC. F) Staining of hepatocytes using Rhodamin 123 as in panel D and anti‐F4/80 antibody staining of KC.

After ruling out a significant influence of ribonucleases, specific mRNA modifications, and ionizable lipids on the translation of synthetic mRNA, we investigated the cell‐specific biodistribution of LNP in vivo, as it might account for the observed differences in mRNA translation. For instance, a rapid excretion of LNP from KC could be the reason for the lower translation of synthetic mRNA in vivo. To this end, we encapsulated 2 types of labeled siRNA into LNP: a commercial negative control siRNA labeled with Alexa Fluor 647 and 2 types of custom‐made negative control siRNA labeled with one or 2 molecules of Alexa Fluor 594 per duplex RNA. Three different types of unlabelled siRNA were used as negative controls (siRNA no. 1–6, **Table** **2**). The fluorophores had a limited impact on the properties of the resulting LNP (LNP no. 8–13, **Table** **3**). We used confocal microscopy to study the LNP8 containing siRNA‐AF647 and LNP9 that were loaded with siRNA‐AF594. We noted that both siRNA exhibited a homogenous distribution over different LNP and that LNP8 led to slightly brighter signals in confocal microscopy (**Figure** **3**A).

**Table 2:** siRNA sequences and fluorescent labels.

siRNA no.	Name of siRNA	Sequence and Label 1 (5′→3′)	Sequence and Label 2 (5′→3′)
1	Negative control siRNA with one 3′ label	Confidential (Qiagen)‐siRNA‐AF647	Confidential (Qiagen)‐siRNA‐AF647
2	Negative control siRNA with one 3′ label	rCrGrUrUrArArUrCrGrCrGrUrArUrA rArUrArCrGrCrGrUA‐T/3AlexF594N/	ArUrArCrGrCrGrUrArUrUrArUrArC rGrCrGrArUrUrArArCrGrArC
3	Negative control siRNA with 2 labels	/5Alex594N/rCrGrUrUrArArUrCrGrCrG rUrArUrArArUrArCrGrCrGrUAT	/5Alex594N/rArUrArCrGrCrGrUrArUrU rArUrArCrGrCrGrArUrUrArArCrGrArC
4	Unlabelled Hprt1‐Dsi‐RNA	Confidential (IDT)	Confidential (IDT)
5	Negative control siRNA	rGrCrUrArCrUrUrUrArCrCrUrUrGrArCrArU	rArUrArArGrArGrArUrGrUrCrArArGrArUrArArArGrUrA
6	Tlr4 Dsi‐RNA	Confidential (IDT)	Confidential (IDT)

Table legend: r: following base is RNA; no prefix = DNA; 3AlexF594N: 3′ coupled Alexa Fluor 594; AF647: Alexa Fluor 647.

**Table 3:** Physicochemical characterization of LNP with unlabelled and labelled siRNA. The siRNA used here can be seen in Table 2. All LNP were generated at LS. Mean data of at least 3 measurements ± SEM.

LNP no.	Content	Size by NS [nm]	Concentration by NS [LNP/mL]	Size by DLS [nm]	Zetapotential [mV]
8	siRNA 1 (AF647)	109.2 ± 0.6	6.61E+08 ± 2.70E+08	129 ± 0.3	−4.72 ± 0.99
9	siRNA 2 (AF594)	145.4 ± 2.6	4.43E+08 ±7.45E+07	154.53 ± 1.53	−3.11 ± 0.5
10	siRNA 3 (AF594×2)	76.6 ± 2.2	1.28 E+09 ± 4.92E+07	108.4 ± 1.86	−5.24 ± 0.16
11	siRNA 4 (UL Ctrl)	105.8 ± 4.9	1.94E+09 ± 9.17E+07	75.65 ± 4.09	‐ 4.73 ± 0.99
12	siRNA 5 (UL Tlr4‐Ctrl)	127.5 ± 26.1	5.74 ± 0.45E+08	104.2 ± 0.3	−0.24 ± 0.756
13	siRNA 6 (UL Tlr4)	116.9 ± 24.6	5.86 ±0.32E+08	105.2 ± 0.793	−3.76 ± 0.998

Table legend: NC: negative control; NS: NanoSight; ND: not done; UL: unlabelled.

**Figure 4:**
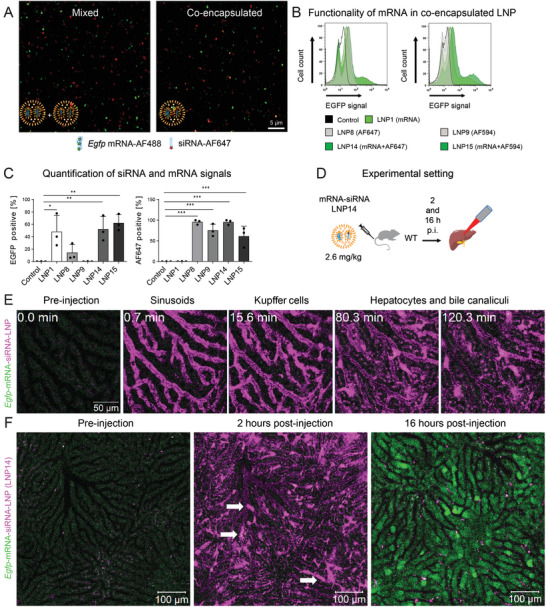
In vivo validation of mRNA‐siRNA LNP co‐encapsulation. LNP with siRNA or mRNA were either generated separately and mixed at equal volumes or co‐encapsulated at a ratio of 1:1 (mRNA:siRNA). A) Confocal imaging of mRNA‐siRNA co‐encapsulation into LNP: left side: AF488‐labelled *Egfp* mRNA‐LNP mixed with AF647‐siRNA‐LNP, right side co‐encapsulated. B) Flow cytometric analysis of the EGFP signals observed from human macrophages transfected with co‐encapsulated *Egfp* mRNA‐LNP on day 6 for 24 h and C) quantifications thereof. D) Intravital microscopy (IVM) setup, p.i.: post‐injection E). Selected time‐points of IVM with *Egfp* mRNA co‐encapsulated with AF647‐siRNA into LNP for the first 2 h and F) 16 h post intravenous injection. White arrows point at KC that internalized LNP and appear purple. Data represent mean of *n* = 3 ± SD; *p < 0.05, **p < 0.01, ***p < 0.001 (One‐way ANOVA).

Following this, we evaluated the uptake of selected formulations by human primary macrophages in vitro after incubating the cells with the LNP for 24 h at the concentration of 1 µg mL^−1^. The signal intensity of LNP8 was closely followed by those of the LNP labeled with either one (LNP9) or 2 groups of AF594 (LNP10). Intriguingly, the presence of 2 labels on a siRNA did not result in increased signals compared to siRNA with a single label. The LNP11 was used as a negative control without a label (like LNP12 and LNP13, not shown for the purpose of clarity), and as expected, these LNP did not cause a signal in flow cytometry (Figure 3B). We also confirmed that cultured murine KC efficiently internalized LNP8 (FigureS4E, Supporting Information).

To study the biodistribution of the siRNA‐LNP in vivo, we initiated intravital imaging immediately after injecting LNP8 at a dosage of 0.6 mg k^−1^g body weight (Figure 3C). Within seconds after intravenous injection, LNP8 appeared in the liver sinusoids and immediately enriched in KC. Approximately 15 min after injection, the siRNA‐LNP was observed in hepatocytes and became concentrated in the bile canaliculi (Figure 3D, Movie S1, Table S5, Supplementary Movie). LNP with AF594‐siRNA (LNP9, Table 3) showed a similar longitudinal biodistribution as LNP8 (Figure S5A, Movie S2, Supplementary Movie). In contrast to LNP‐encapsulated labeled siRNA, free AF647‐siRNA (siRNA1, Table 2) was only transiently visible in the hepatic sinusoids with neither enrichment in KC nor hepatocytes (Figure S5B, Movie S3, Supplementary Movie).

These data demonstrate that the highest spatial accumulation of LNP with labeled siRNA occurred in KC followed by hepatocytes. Therefore, it was clear that differences in the biodistribution or excretion did not account for the differences in mRNA translation.

### 2.4 Co‐encapsulated mRNA and siRNA are efficiently taken up by both Kupffer cells and hepatocytes, but visible translation exclusively occurs in hepatocytes

Previous experiments illustrate that the uptake of LNP containing labeled RNA was most pronounced in KC, followed by hepatocytes. We then posed the question of whether LNP co‐encapsulating mRNA and siRNA would also be taken up by KC at a comparable rate. For this purpose, AF488‐labelled mRNA as used in LNP2 was either first mixed with AF647‐siRNA or AF594‐siRNA and then encapsulated at equal ratios (1:1) (co‐encapsulation) or ready‐made LNP2 were mixed with LNP8 at a ratio of 1:1 (mono‐encapsulated LNP). Confocal microscopy of LNP revealed co‐encapsulation of mRNA and siRNA within the same LNP (**Figure** **4**A). Evaluations of confocal microscopy indicated that ≈80% of the mRNA and siRNA were concurrently coencapsulated (Figure S6, Supporting Information). The physicochemical properties of the co‐encapsulated LNP14‐LNP17 (as shown in **Table** **4**) were comparable to the LNP containing exclusively mRNA (Table 1) or siRNA (Table 2).

**Table 4:** Physicochemical characterization of LNP with coencapsulated mRNA and siRNA. All mRNA and siRNA were co‐encapsulated at a ratio of unmodified *Egfp* mRNA:siRNA at a ratio of 2:1.

LNP no.	Content	Size by NS [nm]	Concentration by NS [LNP/mL]	Size by DLS [nm]	Zeta potential [mV]
14 (LS)	*Egfp* mRNA + siRNA1 (AF647)	153.9 ± 0.6	9.90E+007 ± 1.50E+08	138.97 ± 2.35	−4.73 ± 0.99
15 (HS)	*Egfp* mRNA + siRNA2 (AF594)	103.8 ± 1.2	3.38E+011 ± 8.81E+09	129.03 ± 0.81	−4.88 ± 0.41
16 (HS)	*Egfp* mRNA + siRNA5 (NC siRNA)	118.8 ± 30.5	5.45 ± 0.62E+08	114.1 ± 0.78	−2.98 ± 0.62
17 (HS)	*Egfp* mRNA + siRNA6 (Tlr4‐siRNA)	129.4 ± 38.4	4.63 ± 0.87E+08	116.3 ± 0.14	−5.01 ± 0.28

Table abbreviations: DLS: Dynamic light scattering; HS: high scale; LS: low scale; mV: milli Volt; NC: negative control; NS: NanoSight; UL: unlabelled.

To investigate the potential influence of the co‐encapsulated labeled siRNA on mRNA translation, we incubated human primary macrophages with LNP14‐LNP17 that contained both mRNA and siRNA. Subsequent flow cytometric analyses revealed that the EGFP signal was not negatively affected by the siRNA as shown in representative overlays (Figure 4B). Vice versa, the red signal from the siRNA was not affected by the presence of mRNA in the same LNP (Figure 4C).

Following the characterization and validation of the mRNA‐siRNA co‐encapsulation, we next performed intravital imaging in the first 2 h (to characterize the uptake by hepatocytes and KC) and also 16 h after injection (to study the translation of the *Egfp* mRNA into proteins) (Figure 4D). Similar to the LNP8 containing only siRNA, the LNP with co‐encapsulated siRNA and mRNA was initially highly enriched in KC immediately after injection, followed by accumulation in hepatocytes and secretion into bile canaliculi (Figure 4E, Movie S4, Supplementary Movie). Interestingly, but consistent with the previous findings, EGFP translation also after 16 h was observed solely in hepatocytes. The siRNA‐derived purple signals were significantly reduced after 16 h and were only visible as small spots within the KC (Figure 4F; Movie S5, Supplementary Movie).

### 2.5 Effects of mRNA‐siRNA‐LNP on Hepatocytes and KC In Vivo

**Figure 5:**
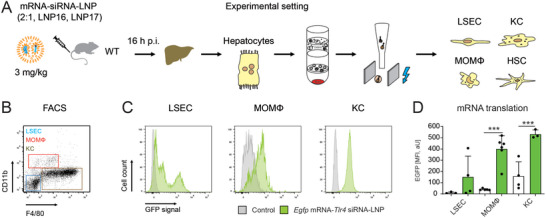
Translation of synthetic mRNA by non‐parenchymal liver cells in vivo. A) Experimental scheme of murine liver cell isolation after LNP‐treatment with co‐encapsulated mRNA (2 mg k^−1^g) and control siRNA (1 mg k^−1^g), in total 3 mg k^−1^g body weight. B) After initial isolation of HC by centrifugation, hepatic LSEC, MOMF, and KC were isolated by a Nycodenz gradient and flow cytometric cell sorting. C) Representative flow cytometric plots from isolation of LSECs and hepatic macrophages, modal Y‐axis settings. D) Cell type‐specific quantification of EGFP signal by mean fluorescence intensity (MFI). Data represent the mean of *n* = 6 ± SD; ***p < 0.001 (One‐way ANOVA).

Our findings from IVM suggested that there is a significant disparity in mRNA translation among various cell types within the liver. We therefore sought out molecular proof of the functionality of siRNA and mRNA in parenchymal cells and NPC in vivo. To this end, we generated LNP with coencapsulated *Egfp* mRNA and a negative control siRNA at the ratio of 2:1 mRNA:siRNA (LNP16), and these LNP were injected intravenously at the concentration of 3 mg k^−1^g. At 16 h post‐injection, we isolated subsets of parenchymal cells and NPC from the livers using established methods (**Figure** **5**A).^[^ ^19^ ^]^ These included hepatocytes, CD31^+^ liver endothelial sinusoidal cells (LSEC), CD31^−^F480^+^CD11b^low^ KC, and CD31^−^F4/80^+^CD11b^+^ monocytic macrophages (MoMΦ). The MoMΦ are another subset of hepatic macrophages that originate from circulating monocytes (Figure 5B). We also isolated hepatic stellate cells (HSC) based on their retinol cell inclusions via a UV laser as demonstrated before.^[^ ^19^ ^]^

We quantified the EGFP signal in LSEC, MoMΦ, and KC by flow cytometry and detected signals in each cell type (Figure 5C). Quantitative analysis of mean fluorescence signal intensities revealed that both MoMΦ and KC expressed the EGFP protein at a detectable and significant level, albeit low (Figure 5D). It is crucial to note that both HSC and hepatocytes display a pronounced autofluorescence, which unfortunately renders flow cytometric analysis unfeasible. QPCR‐based detection of *Egfp* mRNA demonstrated that LNP16 were taken up in significant amounts by hepatocytes and KC, whereas HSC did not absorb the LNP at a significant level (Figure S7A, Supporting Information). To further study the effects of the siRNA by knockdown of a target gene, we generated LNP bearing an siRNA against *Tlr4* and the same *Egfp* mRNA (LNP17) and treated mice with the LNP using the identical dose and time frame. The LNP16 containing control siRNA did not lead to a knockdown of *Tlr4* mRNA as expected, whereas the LNP17 caused a significant reduction of *Tlr4* mRNA in HC, KC, and HSC (Figure S7B, Supporting Information).

In summary, the most important readout of this experiment was the fact that KC, MoMΦ, and LSEC, all being important types of NPC, actually did express EGFP fluorescence – but on a very low level which can only be detected with a sensitive method such as flow cytometry.

**Figure 6:**
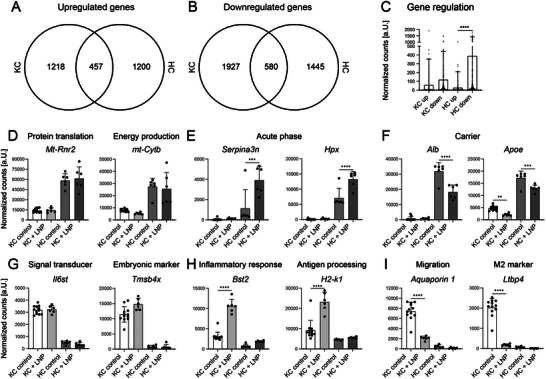
LNP‐induced transcriptomic changes in hepatocytes and Kupffer cells in vivo. Total RNA was purified from HC and Kupffer cells treated as described in Figure 5 followed by RNA sequencing. A) VENN diagram for up‐ and B) downregulated genes of HC and KC. C) Mean counts of the 200 most up and downregulated genes of KC and HC as a measure of LNP effect. D) Hepatocyte‐specific genes unaffected by LNP, E) genes induced, and F) down‐regulated by LNP in HC. G) KC‐specific genes, H) genes up‐regulated and (I) down‐regulated in KC by LNP. Data represent mean of *n* = 6–12 ± SD; *p < 0.05, **p < 0.01, ***p < 0.001, ****p < 0.0001 (One‐way ANOVA).

The next step included RNA seq of the samples purified from HC and KC that aimed to further comprehend our understanding of the individual molecular properties and responses of each cell type. The LNP induced upregulation of similar numbers of genes in both cell types with 1218 in KC and 1200 in HC (**Figure** **6**A). A total of 1927 genes were downregulated in KC and 1445 in HC (Figure 6B). Despite the similar numbers of genes involved in up or downregulation by both cell types, the most pronounced effect of the LNP in both cell types was the downregulation in HC (Figure 6C).

Enrichment analyses of the 300 most significantly upregulated and downregulated genes in each cell type revealed that the genes predominantly affected by LNP in KC were associated with the extracellular space and extracellular regions, regardless of whether they were up or downregulated (Figure S8A,B, Supporting Information). The upregulated genes in hepatocytes exhibited inconclusive patterns, encompassing genes related to protein digestion and absorption, as well as the upregulation of genes associated with neutrophil extracellular traps (Figure S8C, Supporting Information). The downregulated genes of hepatocytes comprised 3 groups of genes associated with the external side of the membrane and the cell surface. The second most prominent gene cluster included 5 groups of genes associated with the endoplasmic reticulum (Figure S8D, Supporting Information).

In the final stage of gene expression analysis, we focused on identifying specific key genes that fell into 3 distinct categories: a) genes specific to each cell type that did not respond to LNP, b) genes upregulated, and c) genes downregulated by LNP in KC or HC.

The *mitochondrial 16S rRNA 2 (mt‐Rnr2)*, a ribosomal RNA involved in mRNA translation, and the *mitochondrial cytochrome b (mt‐Cytb)*, which functions in ATP production and reflects energy expenditure, were identified as HC markers of the first category (Figure 6E). The LNP caused upregulation of the protease inhibitor *Serpin family A member 3 n* (*Serpina 3n*) and *Hemopexin* (*Hpx*), a high affinity heme‐binding protein. Both genes encode for 2 well‐known acute phase proteins (APP) (Figure 6E). Down‐regulation in HC by LNP was noted for the most highly secreted serum protein *albumin* (*Alb*), which is a well‐known negative APP, and for the important serum protein *Apolipoprotein E* (*ApoE*) that is a key factor for lipid transport and which binds to LNP thereby enabling the uptake by hepatocytes through the Low‐density lipoprotein receptor (LDLR) (Figure 6F).

The key marker genes of KC were the *Interleukin 6 Cytokine Family Signal Transducer* (*Il6st*) that encodes for a secretory protein that evokes cytokine signal transmission, and the *Thymosin Beta 4 X‐Linked* (*Tmsb4x*) that encodes a protein with RNA binding activity (Figure 6G). The LNP led to highly significant upregulation of *Bone marrow stromal cell antigen* (*Bst2*) that is associated with inflammatory responses and of *histocompatibility 2, K1, K region* (*H2‐k1*) in KC. The induction of *H2‐k1* in KC by LNP might further affect the activation of T cells by KC (Figure 6H).^[^ ^20^ ^]^ Down‐regulation was noted for a single *Aquaporin* gene, *Aquaporin 1*, which was nearly exclusively expressed by KC, thereby suggesting that the LNP may have an impact on KC migration. Lower mRNA levels were also detected for the *Latent transforming growth factor beta binding protein 4* (*Ltbp4*) that binds to *Transforming growth factor β* (*Tgfb*) (Figure 6I). Supplementary genes are included in the supporting information (Figure S9, Supporting Information).

Overall, the gene expression data enabled a comprehensive understanding of the response of each cell type to LNP: in hepatocytes, the observed upregulation of APP such as *Hp*, *Saa2*, and *Hpx*, coupled with the concurrent downregulation of the negative APP *albumin*, suggests the initiation of an acute phase response. However, it is important to note that APP was also intricately linked to Apolipoprotein pathways, potentially reflecting cellular processes related to the uptake and intracellular transport of lipid nanoparticles.^[^ ^2b^ ^]^ Particularly, the 2 facts that gene expression of *Crp* was not elevated in hepatocytes and that they did not show signs of hepatocyte toxicity in intravital imaging support the notion that the LNP rather affected transport processes than that they caused a pronounced acute phase response, as suggested by earlier own studies.^[^ ^2b^ ^]^

In contrast to hepatocytes, KC constitutively expresses inflammation‐associated markers such as Il6st (Gp130), which enables myeloid KC to exert inflammatory functions in liver disease.^[^ ^21^ ^]^ The specific inflammatory pattern of the KC as triggered by LNP included upregulation of MHC class I markers such as *H2‐k1* which activates T‐cell immunity.^[^ ^20^, ^22^ ^]^ The induction of MHC class I proteins is further supported by the upregulation of *B2m* which encodes a serum protein.^[^ ^23^ ^]^

### 2.6 Differences in the cell biology of HC and KC that influence mRNA translation

**Figure 7:**
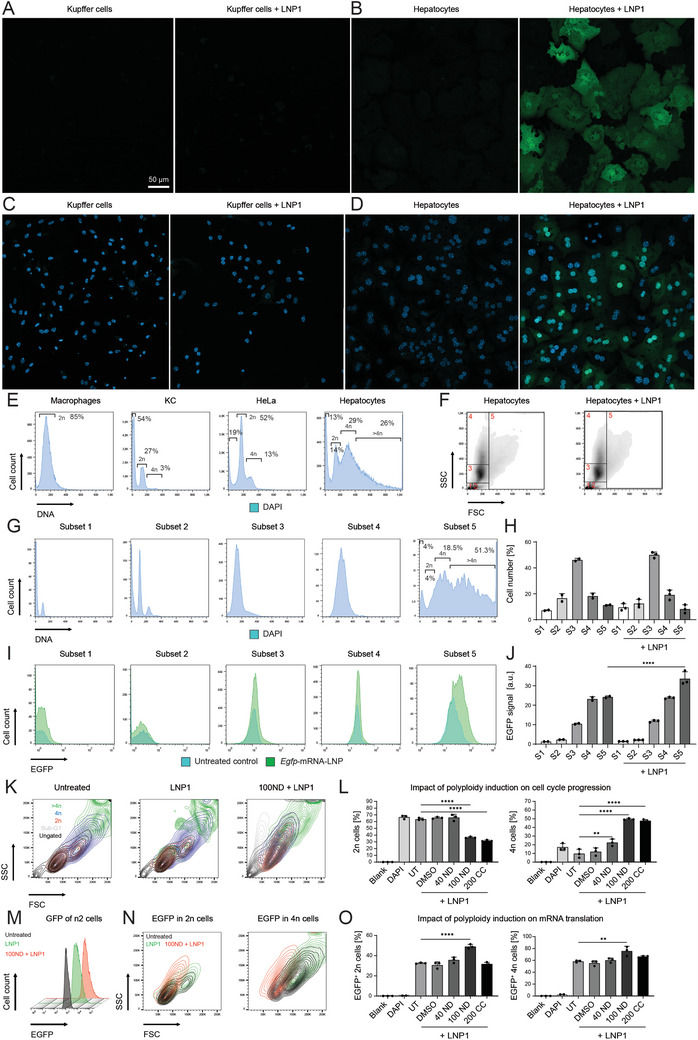
Protein translation and ploidy analysis of primary hepatocytes and Kupffer cells. Primary murine HC and KC were incubated with 1 µg mL^−1^ *Egfp* mRNA‐LNP for 24 h and analyzed by confocal microscopy or flow cytometry. A) Cultures of untreated KC (left) and treated with LNP1 (bar accounts for items A‐D), B) HC treated and recorded as in A, using identical image processing. C) KC as treated in A with additional staining of nuclei, D) nuclei staining of HC treated as in B, recorded as in C. E) Analysis of ploidy of selected cell types by nuclei staining. F) Hepatocyte subsets assigned based on granularity and size from untreated controls or after 24 h of treatment with *Egfp‐*mRNA‐LNP. G) Measurement of DNA content by DAPI staining in the subsets defined in F (left panel), and H) quantifications of the cell numbers in the respective subsets. I) Comparison of the EGFP signals in untreated and transfected HC in the subsets defined in F (left panel), and J) quantifications of the mean fluorescence intensity (MFI). K) Representative flow cytometric plots depicting cellular morphology of Hepa 1–6 cells either kept untreated or treated for 20 h with LNP1 or combination treatment with LNP1 and 100 ng mL^−1^ nocodazole (100 ND). L) In addition to the treatment in K, pre‐treatment was done with 40 ng mL^−1^ nocodazole (40 ND), 200 ng mL^−1^ colchicine (200 CC) compared to Hepa 1–6 cells without treatment (blank), stained with DAPI alone, untreated, or with only LNP1 (UT). Ploidy (2n, 4n) was assessed by flow cytometric analysis of nuclei staining. M) Influence of 100 ND on EGFP‐derived signals in 2n Hepa 1–6 cells. N) Backgating on the morphology of the EGFP^+^ cells in selected groups. Untreated cells are shown as a reference. O) Impact of PP induction on the EGFP expression in Hepa 1–6 cells. Data represent mean of *n* = 2–3 ± SD; *p < 0.05, **p < 0.01, ***p < 0.001, ****p < 0.0001 (One‐way ANOVA).

To explore potential molecular cell type‐specific factors that affect mRNA translation directly or indirectly, we studied primary murine hepatocytes and KC 24 h after incubation with 1 µg mL^−1^ of LNP1. We observed in confocal microscopy that KC exhibited very weak signals (**Figure** **7**A), whereas hepatocytes expressed EGFP in visible amounts throughout the whole cell (Figure 7B). The translation of *Egfp* mRNA into protein was completely inhibited by the antibiotic cycloheximide (Figure S10A, Supporting Information).

In our pursuit to explore the potential correlation between hepatocyte ploidy (specifically nuclei per cell) and mRNA translation, we conducted nuclear staining of the samples. The micrographs demonstrated that murine KC possessed only a single nucleus and exhibited very weak signals (Figure 7C), whereas hepatocytes contained one or 2 nuclei (Figure 7D). The next aim was to study the degree of ploidy between human primary macrophages, murine KC, HeLa cells, and murine hepatocytes. We hence performed nuclear staining of the cell types after 24 treatments with LNP1 as adapted from earlier studies.^[^ ^24^ ^]^ This experiment demonstrated that human primary macrophages that are non‐proliferative exhibited only a single 2n peak in nuclear staining. In contrast to the human macrophages, the murine KC showed a slightly different ploidy profile exhibiting the largest SubG1 population of all cell types with 54%. Cells in the SubG1 phase contain fragmented DNA, which can originate from apoptosis. Based on our experiences with murine KC, this is expected because the cells experience stress from the isolation process. Nevertheless, the KC was partially proliferating, as indicated by a modest 4n peak. The HeLa cells were partially polyploid, with 13% of cells in the 4n phase, consistent with previous reports.^[^ ^25^ ^]^ Hepatocytes clearly exhibited the highest degree of polyploidy with ≈29% of cells being 4n and 26% of the cells being >4n (Figure 7E). The transfection with LNP1 only had a minor impact on the cell cycle progression of hepatocytes (Figure S10B Supporting Information). Quantification of cell numbers across the 4 stages of the cell cycle revealed a slight increase in the 4n and >4n hepatocytes, along with a reduction in 2n cells when hepatocytes were treated with LNP (Figure S10C, Supporting Information).

To study a potential correlation between cellular parameters such as size, granularity, cell cycle progression, and mRNA translation, we categorized the hepatocytes into distinct subpopulations based on their granularity and size. The treatment with LNP did not affect the pattern of hepatocyte subsets (Figure 7F). The first subset consisted of cells with a very small peak corresponding to 2n (diploid) cells, whereas the second subset was composed of slightly larger cells with identical granularity and exhibited an additional peak corresponding to 4n cells. Subsets 3 and 4 combined the size characteristics of subsets 1 and 2 but displayed larger granularity. Notably, subset 5, composed of large cells with varying granularity, exhibited the highest level of polyploidy with ≈70% of cells scoring 4n or greater (Figure 7G). Quantification of flow cytometric signals revealed that there were no statistically significant changes in the cell numbers of the 5 subsets following LNP treatment (Figure 7H).

To study the relation between the ploidy of hepatocyte subsets and *Egfp* mRNA translation, we studied HC that were transfected with LNP1 for 24 h and analyzed the EGFP signals of the different subsets using flow cytometry. We found that the big hepatocytes (subset 5) also expressed the highest levels of EGFP as detected in flow cytometry (Figure 7I), and these were statistically significant in this subset only (Figure 7J).

We further employed 2 small molecules to induce polyploidy in Hepa1‐6 cells through cell cycle arrest during early mitosis. To this end, we either pretreated the cells with 40 or 100 ng mL^−1^ nocodazole or 200 ng mL^−1^ colchicine. Control groups included Hepa 1–6 cells without treatment (blank), stained with DAPI alone, untreated, or only treated with LNP1 for 24 h. We discovered using flow cytometry that 100 ng mL^−1^ nocodazole or 200 ng mL^−1^ colchicine both had a pronounced effect on cellular morphology and led to increased granularity (reflected by sideward scattering light, SSC), and size (reflected by forward scattering light, FSC), compared to treatment with LNP1 or untreated cells (Figure 7K). Statistical quantifications on the cell cycle progression demonstrated that 100 ng mL^−1^ nocodazole or 200 ng mL^−1^ colchicine both led to a significantly reduced number of 2n and a significantly increased number of polyploid 4n cells (Figure 7L). Flow cytometric analysis of the EGFP signals demonstrated that the mRNA translation of the 2n was increased as shown by signal overlays (Figure 7M). Backgating on the EGFP signal in FSC‐SSC gating unraveled that the cells translating EGFP exhibited increased size and granularity (Figure 7N). Statistical quantifications demonstrated that the induction of the EGFP signal reached statistical significance by treatment with 100 ng/mL of nocodazole (Figure 7O). Details on the flow cytometric data evaluation can be found in the supporting information (Figure S10D–G, Supporting Information).

**Figure 8:**
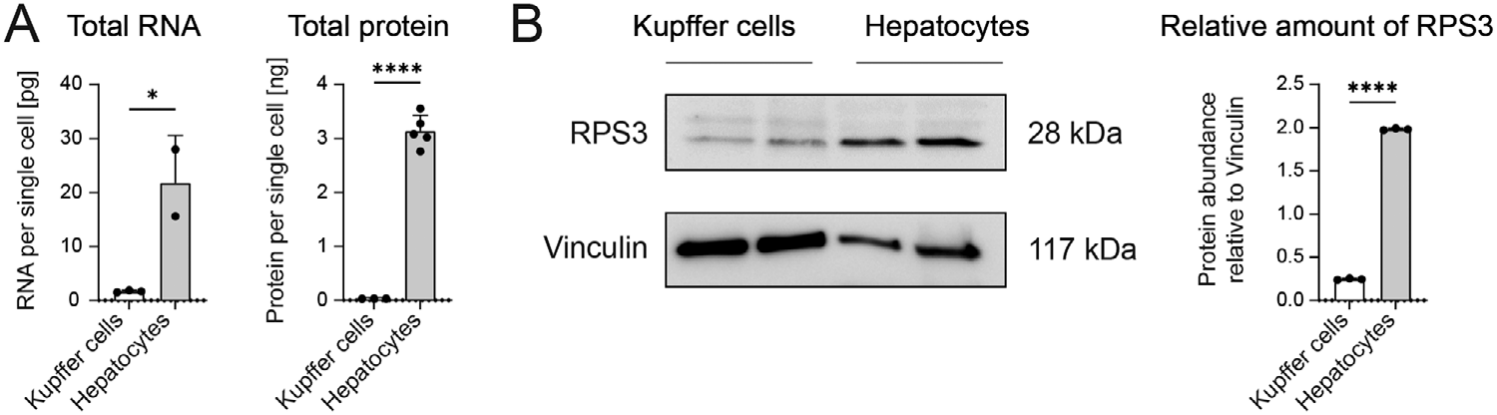
Quantification of total RNA and protein of Kupffer cell and hepatocytes. Primary murine KC and HC were cultured for one day after isolation. Cells were counted and RNA or protein were isolated. A) Quantifications of total RNA and total protein from KC and HC. B) Immunoblot‐based analysis of selected proteins including a quantification of the immunoblot signals. Data represent mean of n = 3 ± SD; **p < 0.01, ****p < 0.0001 (*t*‐test).

After establishing polyploidy as an important novel factor for mRNA translation, we proceeded to investigate whether the polyploid hepatocytes also contain more total RNA and total protein as compared to KC. We therefore isolated total RNA and protein from both cell types and found that the amount of total RNA with 21.8 pg per single hepatocyte was ≈13 times higher than the amount per KC. The amount of total protein in hepatocytes was 3 ng per single cell and thereby 97‐fold higher in hepatocytes compared to KC (**Figure** **8**A). Protein translation by cells requires ribosomes and therefore, we performed an immunoblot to quantify the ribosomal protein 3 (RPS3) in HC and KC. We found that the amount of this key ribosomal protein was significantly more abundant in hepatocytes compared to KC (Figure 8B).

In summary, these data demonstrate that basic cellular differences and, to a certain extent, the degree of ploidy between the parenchymal hepatocytes and the NPC accounts for the magnificent differences in mRNA translation. The role of the biology of individual cell types in mRNA delivery and subsequent translation may previously have been underestimated. This revelation holds significant implications for the RNA delivery domain, as the efficiency of delivery is often deemed the pivotal element for therapeutic efficacy. Our experiments demonstrated that even administering high dosages of mRNA does not enable mRNA translation in NPC.

Our results certainly have implications beyond mRNA‐based therapies for the liver, but potentially also prompt a reassessment of therapeutic strategies for additional organ systems that contain polyploid cells. Typically, mammalian tissues comprise up to 20% polyploid cells.^[^ ^26^ ^]^ Popular examples of polyploid cells are cardiomyocytes (heart muscle cells) which can be polyploid, particularly when they are exposed to stress or during development.^[^ ^27^ ^]^ Megakaryocytes, cells in the bone marrow which give rise to platelets, are commonly also polyploid.^[^ ^28^ ^]^ Placental trophoblast giant cells, which play an important role in the exchange of nutrients between the mother and the fetus, are polyploid,^[^ ^29^ ^]^ and also keratinocytes which are located in the outer layer of the skin can become polyploid during wound healing.^[^ ^30^ ^]^

Cellular polyploidy and the resulting increase in cellular size play an important role in tissue regeneration of the liver, heart, epidermis, and intestine.^[^ ^31^ ^]^ Nevertheless, the deregulation of polyploidy has been linked to genomic instability and cancer and the majority of cancers exhibit aneuploidy, with ≈90% of solid tumors and 75% of hematopoietic cancers showing abnormal chromosome numbers.^[^ ^32^ ^]^ Consequently, the transient emergence of polyploidy could potentially be associated with innovative therapeutic approaches for mRNA delivery.

## 3 Conclusion 

Dysfunctions of the liver can lead to critical disease symptoms not only in the liver as such, but also in distant organs, such as the heart,^[^ ^33^ ^]^ the nervous system,^[^ ^34^ ^]^ or the blood coagulation system.^[^ ^35^ ^]^ This is owed to the fact that hepatocytes generate large amounts of secretory proteins which are transported to liver distant sites.^[^ ^1^ ^]^ Therefore, abnormalities in protein production can affect the entire body and thus understanding liver biology is crucial for curing numerous types of diseases related to protein dysfunction.

Polyploidy, the condition of having more than 2 complete sets of chromosomes, is actually more common in plants than in mammals and a higher harvest of proteins from plants is correlated with polyploidy.^[^ ^36^ ^]^ Polyploidy enables cells to exhibit a larger size, having more chromosomes and gene copy numbers, more total RNA and proteins, and thereby gives hepatocytes an advantage in competition with other cell types in the liver.^[^ ^1^ ^]^ The large amounts of ribosomal proteins such as RPS3 and ribosomal RNA that we detected in hepatocytes illustrate the molecular pattern that enables HC to be potent protein producers.^[^ ^37^ ^]^ Although polyploidy can be induced experimentally by knockdown of E2F Transcription Factor 8 or Anillin in vivo,^[^ ^38^ ^]^ we assume that inducing polyploidy in NPC does not represent a feasible option to enhance mRNA translation because interfering with the cell cycle very likely is accompanied by severe side effects.

We hereby shed light on the discrepancy between mRNA delivery and translation by different liver cell types. It became apparent in our study that the absence of mRNA translation in KC is not due to a reduced uptake of RNA‐loaded LNP, but that it is owed to a fundamentally different cell biology. Hence, our data help to understand the huge success of nucleic acid‐based drugs in hepatocytes.^[^ ^3^ ^]^ We conclude that neither generating sophisticated LNP formulations nor other types of carriers for mRNA can overcome this lack of mRNA translation by NPC because the diploidy of NPC limits their amount of total RNA and protein per single cell.

## 4 Experimental Methods

### RNA and Lipids

Negative control siRNA was purchased from IDT (Coralville, IA, USA) (available and commercial sequences shown in Table 2). Negative control siRNA labeled with Alexa Fluor 647‐labeled (AllStars siRNA) was obtained from Qiagen (Venlo, Netherlands). Heptatriaconta‐6,9,28,31‐tetraen‐19‐yl‐4 (dimethylamino) butanoate (DLin‐MC3‐DMA, MC3) was obtained from Hycultec GmbH (Beutelsbach, Germany). The ionizable lipid SM‐102 was obtained from Cayman Chemical (Michigan, USA). The helper lipid 1,2‐distearoyl‐sn‐glycero‐3‐phosphorylcholine (distearoly‐phosphatidlycholine) (DSPC), and the Polyethylene (PEG) lipid 1,2‐dimyristoyl‐rac‐glycero‐3‐methoxypolyethylene glycol‐2000 (DMG‐PEG 2000), and cholesterol were obtained from Sigma‐Aldrich (St Louis, MO, USA). CleanCap EGFP mRNA and CleanCap EGFP mRNA fully substituted is obtained with (5moU) from Trilink (San Diego, CA, USA). Egfp mRNA fully substituted with 1‐Methylpseudouridine (m1Ψ), generated with PureBoost technology, was acquired from Cellerna (Baesweiler, Germany).

### Materials used for the Custom Synthesis of mRNA by In Vitro Transcription (IVT)

HiScribe T7 High Yield RNA Synthesis Kit (New England Biolabs), HiScribe T7 mRNA Kit with CleanCap Reagent AG (New England Biolabs), Monarch RNA Cleanup Kit (500 µg) (New England Biolabs), 5‐Propargylamino‐CTP‐PEG5‐AF647 (Jena Bioscience), 5‐Propargylamino‐CTP‐PEG5‐AF488 (Jena Bioscience, c = 10 mM), 5‐Propargylamino‐CTP‐PEG5‐AF647 (Jena Bioscience, c = 10 mM), RiboLock RNase Inhibitor (40 U/µl) (Thermo Scientific), ElectroMAX Stbl4 competent cells (Invitrogen), CloneJET PCR‐Cloning Kit (Thermo Scientific), GenElute HP Plasmid Maxiprep Kit (Sigma‐Aldrich), FastDigest XbaI (Thermo Scientific), FastDigest NotI (Thermo Scientific), Monarch DNA Gel Extraction Kit (New England Biolabs).

### Synthesis of Custom mRNA

The AF488 labeled mRNA was synthesized using a HiScribe T7 High Yield RNA Synthesis Kit (New England Biolabs) with an amended kit protocol: diethylpyrocarbonate (DEPC) treated water (3 µL), RNAse Inhibitor (2 µL), 10x Reaction Buffer (4 µL), ATP (4 µL), CTP (3 µL), 5‐Propargylamino‐CTP‐PEG5‐AF488 (10 µL), GTP (4 µL), UTP (4 µL), linearized DNA template (2 µL, c = 2 µg µL^−1^) and T7 Polymerase (4 µL) were gently mixed by pipetting. The mRNA was generated via the T7 mRNA Kit with CleanCap Reagent AG (New England Biolabs) following the protocol: DEPC treated water (8 µL), RNAse Inhibitor (2 µL), 10x CleanCap Reagent AG Reaction Buffer (4 µL), ATP (4 µL), CTP (4 µL), GTP (4 µL), UTP (4 µL), Clean Cap AG (4 µL), linearized DNA template (2 µL, c = (2 µg µL^−1^)) and T7 Polymerase (4 µL) were mixed by pipetting. Following, both reaction mixtures were vortexed and incubated at 37 °C for 2 h before cleaning them up with the Monarch RNA Cleanup Kit (500 µg) following the instructions of the manufacturer.

### Fabrication of RNA‐LNP

Lyophilized siRNA was dissolved in a sterile 0.1 m acetate buffer of pH 4. The low‐scale (LS) encapsulation of RNA (up to 13 µg) was done using a NanoAssemblr Spark (Precision Nanosystems Inc., Vancouver, Canada). High‐scale (HS) encapsulation of ≥100 µg RNA was done using 2 PHD ULTRA syringe pumps (Harvard apparatus) connected to a herringbone microchip (Microfluidic Chip Shop, Jena, Germany). The lipid components (MC3, DSPC, Chol, and PEG2000‐lipid) were dissolved in absolute ethanol and mixed at a molar ratio of (50:10:38.5:1.5) as described earlier.^[^ ^2b^ ^]^ For LS LNP, the lipid mix was adjusted to 50 mM and setting 3 of a NanoAssemblr Spark was used. For HS LNP production, the lipid mix was adjusted to 12.5 mM and the generated LNP products were diluted 1:10 with sterilized phosphate‐buffered saline (PBS) and subsequently concentrated using molecular weight cut‐off concentrators (Sartorius Vivaspin 20, 100000 MWCO PES, Cat. no. VS2042). For co‐encapsulation of mRNA and siRNA, we mixed both mRNA and siRNA in a sterile 0.1 m acetate buffer of pH 4 at specific ratios. LNP was stored at 4° C until usage and used within 48 h.

### Imaging‐Based and Physicochemical Characterization of LNP

We used a NanoSight NS300 (Malvern Instruments, Malvern, UK) with a 488 nm filter to obtain sizes, concentrations, and images of LNP. The cuvette and filter were both carefully cleaned using isopropanol and dust‐free tissues. Then, the cuvette was attached to the filter using screws. Using a syringe, 1 mL of sterile Milli‐Q water with a maximum conductivity of 0.55 µS cm^−1^ was rinsed through the cuvette before attaching the tubing. To prepare HS LNP for the measurement, the RNA content of the LNP from HS was measured by a RiboGreen assay and then they were adjusted to a concentration of 1 µg mL^−1^ in PBS. For the LS LNP, these were diluted 1:200 in PBS and the values were multiplied with the dilution factor 200. Subsequently, another 1 mL syringe containing the LNP suspension was attached to the cuvette. The cuvette was filled completely with the LNP suspension, with at least 0.2 mL remaining in the syringe. The camera was focused and the software NanoSight NS300 NTA v1.0 was set to fivefold measurement with 60 s each and active temperature control (25 °C). After starting the measurement, the syringe was used to convey the suspension by 0.5 mL when prompted. After every LNP measurement, the cuvette was rinsed with 10 mL Milli‐Q water. Before storage, the cuvette and filter were cleaned with Milli‐Q water and dust‐free tissues as well as dried using compressed air. After the experiment, automatic image analysis was done using the Nanosight NTA software 3.44, resulting in mean (number average) LNP size and concentration for every 5 measurements done per sample.

### Light Scattering‐Based Characterization of LNP

For the measurement of the LS LNP, 5 µL of LNP suspension was diluted in 65 µL of PBS. In the HS production, first, the concentration of RNA in the LNP was determined by a Ribogreen assay, adjusted to the concentration of 1 µg mL^−1^ and then 5 µL of the LNP suspension was diluted in 295 µL PBS. The diluted suspensions were transferred to ZEN0040 cuvettes and placed in a Malvern Instruments Nano ZS ZEN3600. The Z‐average of the particles was measured 3 times. During the measurement, an attenuation of less than 11 and a count rate of less than 500 kcps were ensured. The measured distribution was checked for multiple peaks caused by impurities or particle degradation. For further analysis, the calculated mean of each individual measurement was used. Additionally, the polydispersity indices (PDI) were recorded.

### Zeta Potential Measurements

The Zeta potential (ζ) was determined using a Malvern Instruments Nano ZS ZEN3600. The LNP suspension of each sample was diluted 1:100 with DPBS to a final volume of 1 mL. After rinsing the used DTS1070 cuvette with DPBS and air using a 1 mL‐syringe, the diluted LNP suspension was slowly transferred into the cuvette using a syringe. The cuvette was closed with caps on both ends. After checking for the absence of air bubbles in the cuvette and complete coverage of the electrodes, the cuvette was placed in the Malvern Nano ZS. The Z‐average of the particles was measured 3 times and an attenuation below 11 was noted. Afterward, the cuvette was removed, emptied, and rinsed with DPBS and air as before for further use or storage. The values acquired for each individual measurement were used for further data analysis.

### Mouse Experiments

C57Bl/6J wild‐type (WT) mice were obtained from Janvier (France). Animals for all experiments except for the intravital imaging were housed under specific pathogen‐free conditions at the animal facility of the University Hospital Aachen, as approved by the LANUV NRW under the animal grant number 81‐02.04.2020.A302. The experiments were done in accordance with the EU Directive 2010/63/EU for animal experiments and the ARRIVE guidelines (https://arriveguidelines.org/arrive‐guidelines). Mice were treated as described in the respective parts.

### Intravital Imaging

Functional intravital imaging of the livers of anesthetized mice was performed at IfaDo in Dortmund using inverted two‐photon (LSM MP7) or confocal (LSM 880) microscopes (Zeiss, Germany), as previously described.^[^ ^9^, ^39^ ^]^ Before recording, some of the mice received a bolus tail vein injection of rhodamine123 (Thermo Fisher Scientific); a marker of mitochondrial membrane potential that allows visualization of liver morphology and lobular zonation.^[^ ^9b,d^ ^]^ KC was visualized intravitally by tail vein injection of a fluorescently labeled F4/80 antibody (Thermo Fisher Scientific), as described before.^[^ ^39^ ^]^ The LNP or free siRNA was administered during imaging via mouse catheters (SAI Infusion Technologies, USA) fixed in the tail veins.

### Isolation and Fluorescence‐Activated Cell Sorting of Liver Cells

Primary hepatic NPC (KC, and LSEC, and HSC) were isolated using a modified earlier protocol for liver digestion,^[^ ^19^ ^]^ and a specialized method was used to isolate hepatocytes (for details see Supporting information).

### Cell Culture

Primary murine KC was isolated as described in detail in the supporting information. KC was cultured at a concentration of 3 × 10^6^ cells/mL by resuspension in RPMI1640 medium supplemented with 1% Pen/Strep, 10 ng mL^−1^ M‐CSF, and 10% FCS (of which 30% was L929‐conditioned medium) on Petri dishes for 2 h, subsequently, the supernatant was removed and replaced with fresh medium. To generate human primary macrophages, peripheral blood mononuclear cells (PBMC) were isolated from blood as published before.^[^ ^40^ ^]^ Experiments with human primary immune cells were approved under the ethics approval number CTC‐A 276 or obtained from Transfusion Medicine of the University Hospital Aachen.

The cell lines J774A.1 (ATCC TIB‐67), Hepa1‐6 (ATCC CRL‐1830), NCTC clone 929 (L929 fibroblasts; ATCC CCL‐1), the human endothelial cell line EA.hy926 (ATCC CRL‐2922), and HeLa cells (ATCC CCL‐2) were purchased from the American Type Culture Collection (ATCC, Manassas, VA, USA) and cultured in Dulbecco's modified Eagle medium (DMEM, PAN Biotech, Aidenbach, Germany) containing 10% FBS, 100 U mL^−1^ penicillin, and 100 µg mL^−1^ streptomycin. During transfection with LNP, the FBC content was reduced to 5% serum.

### Induction and Detection of Cellular Ploidy

Cellular ploidy was induced by using 40 or 100 ng mL^−1^ Nocodazole (Sigma‐Aldrich, Cat.No: 31430‐18‐9) or 200 ng mL^−1^ Colchicine (Thermo Fisher Scientific, Cat.No: J61072.03) diluted in DMSO. The cells were treated with the inhibitors for 20 h before receiving 1 µg mL^−1^ of LNP1. The ploidy was determined using 4′,6‐diamidino‐2‐phenylindole (DAPI) staining (Thermo Fisher Scientific). After transfection (constantly 5% FCS), 5 × 10⁵ cells were fixed with 4% paraformaldehyde for 15 min at 4 °C, washed 3 times with PBS, and subsequently stained with 200 µL of 1 µg mL^−1^ DAPI solution for 10 min at 4 °C, protected from light. After staining, cells were washed again with PBS to remove excess DAPI and stored in PBS until flow cytometric analysis. DAPI signals were recorded at a linear scale.

### Confocal Microscopy of LNP and Cells

The cells were prepared for imaging by seeding 50000 murine KC or 30000 murine hepatocytes into ibiTreat µ‐Slide 8 Well chamber slides (Ibidi, 80 826) with 200 µL medium. The cells were transfected 24 h before imaging with 1 µg mL^−1^ LNP1, and the imaging was done 2 days after seeding. Confocal imaging was done using the Zeiss Laser Scanning Microscope (LSM) 980 with Airyscan 2, and the ZEN blue 3.6 software with the addition of the LSM Plus module. Additional details can be found in the supporting information.

### Analysis of total RNA, RNA Sequencing, and Data Evaluation

To isolate the total RNA from cell culture samples, the RNeasy Mini Kit (Qiagen, 74 106) was used, according to the manufacturers’ protocol, including the optional DNase on‐column DNase digestion. For the isolation of total RNA from cells isolated by FACS, the RNeasy Plus Micro Kit (Qiagen, 74 034) was used according to the manufacturers’ protocol. The High‐Capacity cDNA Reverse Transcription Kit (Applied Biosystems, 4 368 814), with the addition of RNase inhibitor (Applied Biosystems, N8080119), was used for cDNA synthesis. RNA seq and data evaluation were done as described earlier.^[^ ^2b^ ^]^

### Quantitative Realtime‐PCR

Quantitative real‐time PCR (qPCR) reaction was done in the MicroAmp Fast 96‐Well Reaction Plate (Applied Biosystems, 4 346 907) using the PowerUp SYBR Green Master Mix for qPCR (Applied Biosystems, A25742) and 5 ng of each sample. The measurement was done using a QuantStudio 5 thermocycler (Applied Biosystems). Target‐specific primers were designed using the NCBI Primer‐BLAST tool and the primers were ordered from Eurofins (sequences shown in **Table** **5**). *Gapdh* was used as a reference gene to perform the ΔΔCT data analysis as previously described by Livak and Schmittgen in 2001.^[^ ^41^ ^]^ The quantification of the uptake of *Egfp* mRNA by different cell types was done by relating the *Egfp* mRNA as parts per million of the total RNA, based on a calibration curve obtained (Figure S2, Supporting Information) with pure *Egfp* mRNA (resulting data are shown in Figure 2A; Figure S7A, Supporting Information).

**Table 5:** Sequences of DNA primers for Realtime‐PCR.

Primer	Species	Sequence (5′–3′)
*Egfp*	Jellyfish	Forward: AAGCAGAAGAACGGCATCAA Reverse: GGGGGTGTTCTGCTGGTAGT
*Gapdh*	Mouse	Forward: TGTTGAAGTCACAGGAGACAACCT Reverse: AACCTGCCAAGTATGATGACATCA
*Rnase1*	Mouse	Forward: CCCCACCTACTGCAACCAAA Reverse: GACATCTGCCAAGGGCTCAT
*Rnh1*	Mouse	Forward: GCTTCGTTCTTCCTGAGACTG Reverse: CCAGCCTGACCACTTCGTAT
*Tlr4*	Mouse	Forward: TCCCTGCATAGAGGTAGTTCC Reverse: TCAAGGGGTTGAAGCTCAGA
*Zc3h12a*	Mouse	Forward: TGTGCCTATCACAGACCAGC Reverse: ACGTGTCATTGGAGACCACC
*Ang*	Mouse	Forward: TTCGCCATCCCAACAGGAAG Reverse: CCAACAGAGATTCCTGGACCC
Gapdh	Human	Forward: GACAGTCAGCCGCATCTTCT Reverse: AAATGAGCCCCAGCCTTCTC
*Rnase1*	Human	Forward: GCTGCAGATCCAGGCTTTTCTGGG Reverse: GCTGCTGCTGGGGGAACTGTC
*Rnh1*	Human	Forward: GATCTGGGAGTGTGGCATCA Reverse: CTGCAGGACTTCACCCACAG
*Zc3h12a*	Human	Forward: CGCATGGCGGGTAGGAG Reverse: GGACAGGCTTCTCTCCACAG
*Ang*	Human	Forward: ATTCTTCCTCCTGGGAGCCTG Reverse: GGGTCAGGAAGTGTGTGTACC

### Isolation and Quantification of Cellular Protein

Primary human macrophages and mouse hepatocytes were homogenized in ice‐cold NP40 lysis buffer (50 mM Tris‐HCl pH 7.5, 150 mM NaCl, 0.5% NP‐40, and 50 mM NaF) supplemented with Complete Mini (Roche), PhosSTOP (Roche), 1 mM orthovanadate, and 1 mM phenylmethylsulfonyl fluoride (Pefablock). Isolated protein was quantified using a Bradford assay (Biorad) and the same amounts of protein (10–40 µg) were dissolved in 12% sodium dodecyl sulfate‐polyacrylamide gel electrophoresis (SDS‐PAGE), and then transferred to a nitrocellulose membrane. Unspecific binding sites were blocked in TBST (10 mM Tris‐HCl, 150 mM NaCl, 0.1% Tween 20, pH 7.6) containing 5% milk powder. Immunoblotting was performed using the following primary antibodies: RPS3 Polyclonal Antibody (binding both murine and human RPS3) (Thermo Fisher Scientific, dilution 1:1000), Anti‐GFP antibody (3H9) (Abcam, ab252881, dilution 1:1000), Anti‐Vinculin antibody (Abcam, ab129002, dilution 1:5000) and Beta‐actin (Bio‐Rad, Munich, dilution 1:1000). Anti‐rabbit‐HRP (Cell Signalling, dilution 1:5000) and anti‐mouse‐HRP (Santa Cruz, dilution 1:5000) was used as secondary antibodies. The light emission of the protein of interest was determined using ImageQuant LAS 4000 software (GE Healthcare, Chicago, IL, USA). The immunoblots shown in the figures were optimized in brightness in contrast, and the sequence of appearance of some bands was changed for presentation clarity. Original blots are deposited in Zenodo as stated below.

### Inhibition of Protein Translation

A solution of cycloheximide in DMSO (Sigma Aldrich, Catalog No. C4859) was employed to impede protein synthesis. This was achieved by adding the solution at a final concentration of 10 µg mL^−1^ to the cell culture medium, 1 h prior to the transfection with LNP1.

### Statistical Analysis

There was no pre‐processing of data that was not mentioned in the respective method sections. The data are expressed as mean ± SD or mean ± SEM. The sample sizes are shown below each figure for each experimental group. Differences between groups with *p < 0.05, **p < 0.01, ***p < 0.001, ****p < 0.0001 were considered significant. We utilized One‐way ANOVA for multiple group comparisons and a *t*‐test for the comparison of 2 groups. Statistical analysis of data was conducted using GraphPad Prism 10 (GraphPad Software, La Jolla, USA).

## Conflict of Interest

The authors declare no conflict of interest.

## Author Contributions

C.L. conceived and M.B. designed the mouse experiments, M.P. and I.V. designed cell sorting, A.K., L.L., N.W., and P.B.S. isolated and analyzed RNA. E.S. and G.M.‐N. performed the confocal microscopy. C.P. and C.L. developed a diploidy assessment, E.Z. and L.L. performed cell culture experiments, and T.H. and A.H. generated synthetic mRNA. All authors discussed the results and reviewed the manuscript.

## Supporting information



Supporting information

Supplementary Movie1

Supplementary Movie2

Supplementary Movie3

Supplementary Movie4

Supplementary Movie5

## Data Availability

The data that support the findings of this study are openly available in GEO (RNA seq data), Zenodo (all other original data) at 10.5281/zenodo.14511049, reference number 14511049.
