# Supplementary material for: Exceptional Uptake, Limited Protein Expression: Liver Macrophages Lost in Translation of Synthetic mRNA
Source: Adv Sci (Weinh). 2025 Jan 10;12(9):2409729. doi: 10.1002/advs.202409729 (PMC11884593; doi:10.1002/advs.202409729)
Supplement: Supplementary file 1 — Supporting information [file ADVS-12-2409729-s001.docx]

Supporting Information

**Exceptional Uptake, Limited Protein Expression: Liver Macrophages Lost in Translation of Synthetic mRNA**

Cheng Lin ^1,2^, Adrian Kuzmanović ^1^, Nan Wang ^1,3^, Liangliang Liao ^1,4^, Sabrina Ernst ^5^, Christian Penners ^1^, Alexander Jans ^1^, Thomas Hammoor ^6,7^, Petra Bumnuri Stach ^1^, Mona Peltzer ^1^, Ines Volkert ^1^, Elisabeth Zechendorf ^8^, Reham Hassan ^9,10^, Maiju Myllys^9^, Christian Liedtke ^1^, Andreas Herrmann ^6,7^, Gurudas Chakraborty ^6^, Christian Trautwein ^9^, Jan Hengstler ^9^, Gerhard Müller-Newen ^11^, Junqing Wang ^3^, Ahmed Ghallab ^9,10^, and Matthias Bartneck ^1,6,7*^

**Table of contents**

Figure S1: Microscopical imaging of *Egfp* mRNA-LNP (LNP1) .......................................................................... **2**

Details on qPCR for detection of *Egfp* mRNA by qPCR (Figure S2, Table S1) ................................................... **2**

Details on liver cell isolation .................................................................................................................................. **3**

Mycoplasma detection (Figure S3, Table S2-4) ..................................................................................................... **4**

Table S5: List of movies ......................................................................................................................................... **6**

Details on confocal microscopy .............................................................................................................................. **6**

Figure S4: Impact of ribonuclease 1 inhibition on mRNA translation.................................................................... **8**

Figure S5: Intrahepatic distribution of siRNA-LNP and free siRNA ..................................................................... **9**

Figure S6: Analysis of mRNA-siRNA coencapsulation into LNP ...................................................................... **10**

Figure S7: QPCR-based validation of RNA seq .................................................................................................. **11**

Figure S8: Gene enrichment analysis of LNP-affected genes ............................................................................ **11**

Expanded discussion on the effects of LNP on gene expression of HC and KC (Figure S9) ........................ **12-13**

Figure S10: Microscopy of hepatocytes and macrophages treated with *Egfp* mRNA-LNP................................ **14**

Details on the *in vitro* transcription for synthesis of mRNA................................................................................ **15**

DNA Template generation ................................................................................................................................... **16**

Table S6-S9: List of the top 300 up/down genes in KC and HC ..................................................................... **17-44**

Supplementary references .................................................................................................................................... **44**

**
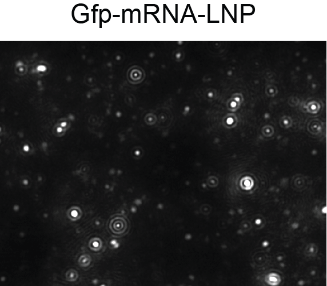
**

**Figure S1: Microscopical imaging of *Egfp-*mRNA LNP.** LNP1 were generated using microfluidic mixing and characterized using NanoSight. Please note that a scale bar cannot be added since the size of the particles is calculated based on their movement.

**Details on qPCR for detection of *Egfp* mRNA by qPCR**

Quantification of the *Egfp* mRNA in experimental RNA samples through qPCR, was calculated using a simple linear regression model based on Ct values of known amounts of *Egfp* mRNA-derived cDNA. A calibration curve was created using cDNA ranging from 0.00005 pg to 50 pg in 10-fold increments, and a qPCR reaction was performed. Additional inputs outside of the calibration curve were also examined, but the CT values deemed to be unreliable and were excluded from the calibration curve. The resulting CT values were plotted against Log10-transformed input amounts to generate a simple linear equation (**Figure S3**). For each experimental sample, 5 ng of cDNA was used in the qPCR reaction. The CT values from experimental samples were compared to the calibration curve to obtain a corresponding Log10-transformed RNA amount. The values were exponentiated to revert to the original scale and obtain the RNA amount in picograms. To normalize the *Egfp* mRNA against the total RNA content, the RNA amount was divided by 5 ng total cDNA input per reaction (**Table S5**).

**
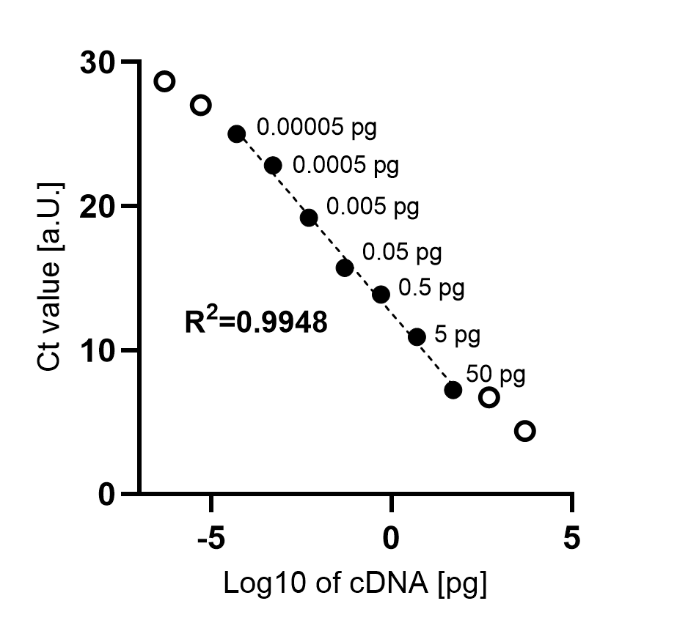
**

**Figure S2:** **Calibration curve for the quantification of *Egfp* mRNA by qPCR.** Calibration curve generated by plotting CT values of known *Egfp* mRNA amounts against the Log10-transformed input amounts.

**Table S1: Excel-based calculation sheet for the quantification of RNA amount** based on Ct values of known amounts of RNA.

| Sample | CT value of sample | Average | Quantity [pg] | Amount of cDNA in qPCR reaction [pg] | Quantity per total amount of RNA [pg/pg of total RNA] | Percentage in total RNA [%] |
| --- | --- | --- | --- | --- | --- | --- |
| Example 1 | 18,90 | 19 | 0,006488745 | 5000 | 1,29775E-06 | 0,00012977 |
| Example 2 | 19 |  |  |  |  |  |
| Example 3 | 19,1 |  |  |  |  |  |
|  |  | = Average (Example 1:Example 3) | =10^((Average - (Y-intercept)) / Slope) |  | = Quantity / (Amount of cDNA in qPCR reaction) | = Quantity per total amount of RNA*100 |
| Calibration curve Y-intercept | 12,552 |  |  |  |  |  |
| Calibration curve slope | -2,947 |  |  |  |  |  |

**Details on liver cell isolation**

*1. Isolation of Kupffer cells and liver sinusoidal endothelial cells:* Mice were sacrificed with a 10% isoflurane overdose. The liver was perfused by inserting a 20G catheter into the Vena cava inferior. The perfusion buffers were prewarmed to 37°C and pumped through the Vena cava inferior while also drained through the Vena portae hepatis at a rate of 5 mL/min. First 25 mL of perfusion buffer A (0.5 mM EGTA, 5 mM glucose, 10 mM HEPES, 5.4 mM KCl, 0.85 mM Na_2_HPO_4_, 137 mM NaCl, 0.57 mM NaH_2_PO_4_ x H_2_O, 4.2 mM NaHCO3, 0.017 mM Phenol Red, sterile filtered) was pumped through the liver, followed by 25 mL of perfusion buffer B (10 mM HEPES, 5.4 mM KCl, 0.85 mM Na_2_HPO_4_, 137 mM NaCl, 0.57 mM NaH_2_PO_4_ x H_2_O, 4.2 mM NaHCO_3_, 3.8 mM CaCl_2_ x 6H_2_O, 0.017 mM Phenol Red, sterile filtered), supplemented with 0.32 mg/mL pronase (Roche, 10165921001). This was followed by another 25 ml of perfusion buffer B supplemented with 0.2 U/mL collagenase D (Roche, 11088858001). The perfused liver was removed and placed into perfusion buffer B supplemented with 0.2 U/ml collagenase D, 0.48 mg/mL pronase and 2 mg/mL DNase I (Roche, 11284932001), for further digestion for 20 minutes in a 37 °C water bath. The cell suspension was filtered through a 70 µm cell strainer and rinsed with 10 mL of GBSS-B (5.5 mM glucose, 5 mM KCl, 0.42 mM Na_2_HPO_4_, 137 mM NaCl, 2.7 mM NaHCO_3_, 1.5 mM CaCl_2_ x 2H_2_O, 0.22 mM KH_2_PO_4_, 1 mM MgCl_2_ x 6H_2_O, 0.28 mM MgSO_4_ x 7H_2_O, 0.017 mM Phenol Red, sterile filtered). The suspension was centrifuged for 2 minutes at 45 rcf and 4 °C to generate a pellet containing the relatively big hepatocytes (these hepatocytes were used for RNA isolation after the *in vivo* experiments).

The supernatant containing KC and LSEC was centrifuged two times for two minutes at 45 rcf and 4 °C, separated again and then once more for another 8 minutes at 720 rcf and 4 °C. The pellet was resuspended in GBSS-B, supplemented with 0.003 mg/mL DNase I and the suspension was centrifuged for 5 minutes at 720 rcf and 4 °C. The subsequent pellet was resuspended in GBSS-B supplemented with 6.25 µg/mL DNase I and 10% of iohexol (Nycodenz). The suspension was carefully layered on top of GBSS-A (5.5 mM glucose, 5 mM KCl, 0.42 mM Na_2_HPO4, 2.7 mM NaHCO_3_, 1.5 mM CaCl_2_ x 2H_2_O, 0.22 mM KH_2_PO_4_, 1 mM MgCl_2_ x 6H_2_O, 0.28 mM MgSO_4_ x 7H_2_O, and 0.017 mM Phenol Red, sterile filtered) supplemented with 14.5% iohexol (Nycodenz). Finally, GBSS-B was layered on top of the two solutions, which were then centrifuged for 20 minutes at 1900 rcf and 4 °C with the centrifuge rotor break turned off. After gradient centrifugation, a layer containing the HSC and the interphase with KC and LSEC were separated into new tubes and washed once with GBSS-B, and finally centrifuged for 5 minutes at 720 rcf and 4 °C.

To isolate KC, cells were stained with antibodies (CD45 (eBioscience 557659), F4/80 (Invitrogen 2198632), CD11b (Invitrogen 2267416) (all diluted 1:400) and CD31 (diluted 1:100), for 15 minutes at 4 °C in Fluorescence-activated cell sorting (FACS) buffer (four parts of PBS and one part mixture of 2% each BSA, rabbit serum, 2% human serum and 2% mouse serum). The cells were centrifuged at 720 rcf and resuspended in DPBS for fluorescence-activated cell sorting using a Becton Dickinson (BD) FACS Aria Fusion. HSC were sorted to receive highly pure HCS which contain retinol and therefore exhibit a peak when emitted by a UV laser. KC were defined as F4/80^+^, CD11b^low^, CD31^-^, CD45^+^. Cells were sorted into the corresponding buffers for RNA isolation.

*2. Isolation of hepatocytes for cell culture:* Hepatocytes isolated using the method described above were utilized for RNA extraction to receive data for up to four different cell types from the same animal. For culturing hepatocytes, cells have to and were isolated by a different protocol. Here, the mice were sacrificed by 10% isoflurane overdose. Prewarmed perfusion buffers (37°C) were pumped through the *Vena cava inferior* using a 20G catheter at a flow rate of 5 mL/min. The *Vena portae hepatis* was transsected immediately. Initially, the liver was perfused with 50 mL of perfusion buffer A (50 mL Earle's Balanced Salt Solution [EBSS, Gibco, 14155048] supplemented with 250 µL of 100 mM EGTA, pH 8.3). This was followed by 30 mL of perfusion buffer B (30 mL EBSS, Gibco, 14155048). Finally, 50 mL of buffer C (50 mL EBSS [Gibco, 24010043] containing 1 mg/mL Collagenase Type IV [Worthington Biochemical, LS004188], 0.1 mg/mL Collagenase P+ [Roche, 11213865001], and 0.04 mg/mL trypsin inhibitor [Sigma-Aldrich, T6522-100MG]) was used.

After perfusion, the liver was removed and placed into bacterial grade petri dishes containing Dulbecco’s Modified Eagle Medium (DMEM, PAN Biotech, Aidenbach, Germany) supplemented with 10% FBS, 100 U/mL penicillin, and 100 μg/mL streptomycin. Subsequently, the liver was transferred to cell culture hood and the organ was gently dissected with a cell scraper. The gall bladder was removed, the liver capsule was ruptured, and hepatocytes were extracted by shaking. The cell suspension was collected and passed through a 70 µm cell strainer (Corning, 352350) into a 50 mL Falcon tube. The cell suspension was centrifuged for 5 minutes at 4°C at 350 rcf. The supernatant was discarded, and the resulting pellet was resuspended in 30 mL of cold DMEM medium (supplemented with 10% FBS, 100 U/mL penicillin, and 100 μg/mL streptomycin) by gently shaking the Falcon tube. This step was repeated twice. Cells were counted using Trypan blue and seed them (0,5 x 10^6^ cells/well) in 6-well plate (Sarstedt, 833920300) with warm culture medium. Let the hepatocytes attached in the cell incubator at 37 °C in a humidified 5% CO_2_ atmosphere for 4 hours. Afterwards, use warm PBS (37 °C) wash the cells twice and replaced with fresh medium at 37 °C.

**Detection of Mycoplasma contaminations in cell cultures**

We routinely assess all cell lines for Mycoplasma contamination. To this end, cell culture supernatants (100 µl) were collected, heated at 95°C for 5 minutes, and centrifuged at 15,000 rcf for 30 seconds and 50 µL of the supernatant were transferred to a new tube for PCR or stored at -20°C until further use. PCR was performed in a 25 µl reaction volume containing 12.5 µl RedTaq (Sigma-Aldrich, Germany), 1 µl forward primer mix (200 nM), 1 µl reverse primer mix (200 nM), 0.5 µl Cre-TO2 forward primer (200 nM), 0.5 µl Cre-TO2 reverse primer (200 nM), 0.7 µl mouse genomic DNA, and 6.8 µL H_2_O. The cycling conditions included an initial denaturation at 95°C for 3 minutes, followed by 35 cycles of 95°C for 10 seconds, 60°C for 20 seconds, and 72°C for 16 seconds (incrementing by two seconds per cycle), with a final extension at 72°C for 5 minutes. PCR products were resolved on a 2% agarose gel with ethidium bromide. A 510 bp band indicated Mycoplasma-positive samples, while a 300 bp band served as an internal control that indicates that the sample was free of Mycoplasma.

**Table S2: Forward Primer Mix**

| **Forward Primer Mix (each 5 µM)** | **Volume** |
| --- | --- |
| Myco-fwd1 (100 µM) | 5 µl |
| Myco-fwd2 (100 µM) | 5 µl |
| Myco-fwd3 (100 µM) | 5 µl |
| Myco-fwd4 (100 µM) | 5 µl |
| Myco-fwd5 (100 µM) | 5 µl |
| Myco-fwd6 (100 µM) | 5 µl |
| Myco-fwd7 (100 µM) | 5 µl |
| H₂O | 65 µl |
| Total | 100 µl |

**Table S3: Forward Primer Mix**

| **Reverse Primer Mix (each 5 µM)** | **Volume** |
| --- | --- |
| Myco-rev1 (100 µM) | 5 µl |
| Myco-rev2 (100 µM) | 5 µl |
| Myco-rev3 (100 µM) | 5 µl |
| H₂O | 85 µl |
| Total | 100 µl |

**Table S4: Primer Sequences**

| **Primer** | **Sequence** |
| --- | --- |
| Myco-fwd1 | CGCCTGAGTAGTACGTTCGC |
| Myco-fwd2 | CGCCTGAGTAGTACGTACGC |
| Myco-fwd3 | TGCCTGGGTAGTACATTCGC |
| Myco-fwd4 | TGCCTGAGTAGTACATTCGC |
| Myco-fwd5 | CGCCTGAGTAGTATGCTCGC |
| Myco-fwd6 | CACCTGAGTAGTATGCTCGC |
| Myco-fwd7 | CGCCTGGGTAGTACATTCGC |
| Myco-rev1 | GCGGTGTGTACAAGACCCGA |
| Myco-rev2 | GCGGTGTGTACAAAACCCGA |
| Myco-rev3 | GCGGTGTGTACAAACCCCGA |
| Cre-TO2-fwd | CTAGGCCACAGAATTGAAAGATCT |
| Cre-TO2-rev | GTAGGTGGAAATTCTAGCATCATCC |


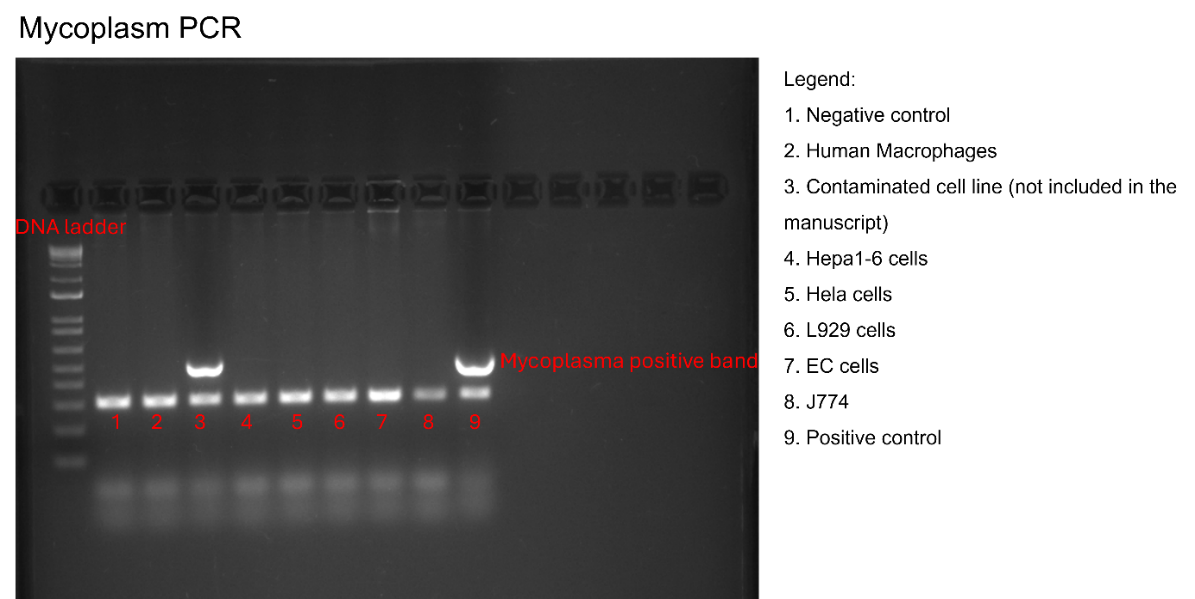


**Figure S3: Results of recent Mycoplasma PCR.** Two bands indicate contamination with mycoplasms.

**Table S5: List of movies generated by intravital microscopy of the liver.**

| **Movie** | **Content description** |
| --- | --- |
| 1 | Hepatocytes were labeled using Rhodamine123 staining. Then the recording began and the LNP8 with siRNA647 are injected and appear as purple color. |
| 2 | AF594-siRNA-LNP at 0.6 mg/kg were injected into mice and the recording of the movie began immediately after injection |
| 3 | Free siRNA was injected at the dose of 0.6 mg/kg and recording was started immediately after injection |
| 4 | *Egfp*-mRNA-AF647-siRNA-LNP (mRNA:siRNA 2:1) were injected into mice and the recording of the movie began immediately after injection. First two hours. |
| 5 | *Egfp*-mRNA-AF647-siRNA-LNP (mRNA:siRNA 2:1) were injected into mice and the recording of the movie began immediately after injection. 16 hours post injection |

**Details on confocal microscopy of cells and LNP**

An LD LCl Plan-Apochromat 40x objective (N.A. 1.2) was used with water immersion and the Axiocam 305 mono was used to capture the wide-field images. The optimal conditions for live cell imaging of hepatocytes and Kupffer cells was set up using the the XLmulti S2 DARK Standard incubator with the TempModul S1 and CO_2_ Modul S1, with the temperature set to 37 °C and the CO_2_ output to 5%. The EGFP signal was imaged using the 488 nm diode laser with 0.4% laser intensity and the GaAsP-PMT detector gain set to 760V, while the DAPI signal was imaged using the 405 nm diode laser, with 0.2% laser intensity and the Multialkali-PMT detector gain set to 750V. Final images consist of four stitched fields of view. The images were post-processed by applying the LSM Plus processing and stitching using the ZEN blue 3.6 software. White balance of the EGFP was set to 35k for both cell types, while the white balance of the DAPI signal was set to 35k or 12k for Kupffer cells or hepatocytes respectively.

To image LNP, 1 µg/mL LNP suspension was added directly to 200 µL DMEM medium and to ibiTreat µ-Slide 8 Well cell plates. Imaging was performed similarly to cells, but without temperature and CO_2_ control in the incubator. For simultaneous imaging of both AF488-mRNA and AF594-siRNA monoencapsualted or co-encapsulated into LNP, the AF594 signal was recorded using a 561 nm diode pumped solid state laser with 0.5% laser intensity and a Multialkali-PMT detector gain at 630V. The AF488 signal was imaged using the using the 488 nm diode laser with 3% laser intensity and the GaAsP-PMT detector gain set to 750V. For combined imaging of the AF488-mRNA and AF647-siRNA monoencapsualted or co-encapsulated into LNP, the AF647 signal was tracked using the 639 nm diode laser with 3% laser intensity and the Multialkali-PMT detector gain set to 800V. The AF488 signal was imaged using the using the 488 nm diode laser with 3% laser intensity and the GaAsP-PMT detector gain set to 720V. The images were post-processed by applying the LSM Plus processing using the ZEN blue 3.6 software. The images were analyzed using the ImageJ 1.54g software and the Just Another Colocalisation Plugin v2.1.4. We evaluated three different pictures from confocal microscopy and used the white balance settings of 25k for AF488 mRNA-LNP and 14k for AF647-siRNA-LNP. Co-encapsulation efficiency was calculated based on single particles that exhibited two overlay signals in three different pictures using ZEISS ZEN 3.0 software. For imaging of the AF647 siRNA-loaded LNP8, the white balance setting was set to 6000 for single LNP images or 4000 while in imaging mRNA LNPs, the black balance was set to 1000. Similarly, when imaging the AF594 siRNA LNP, the white balance was set to 31 000 when imaging single LNPs or 15 000 when imaging with the mRNA LNP, with the black balance set to 2800 or 0 accordingly. For the AF488 mRNA LNP the black balance was set to 3500 when imaging with siRNA-LNPs and 5000 when imaging the mRNA LNP alone, and the white balance was set to 15000 or 22000 accordingly.

**
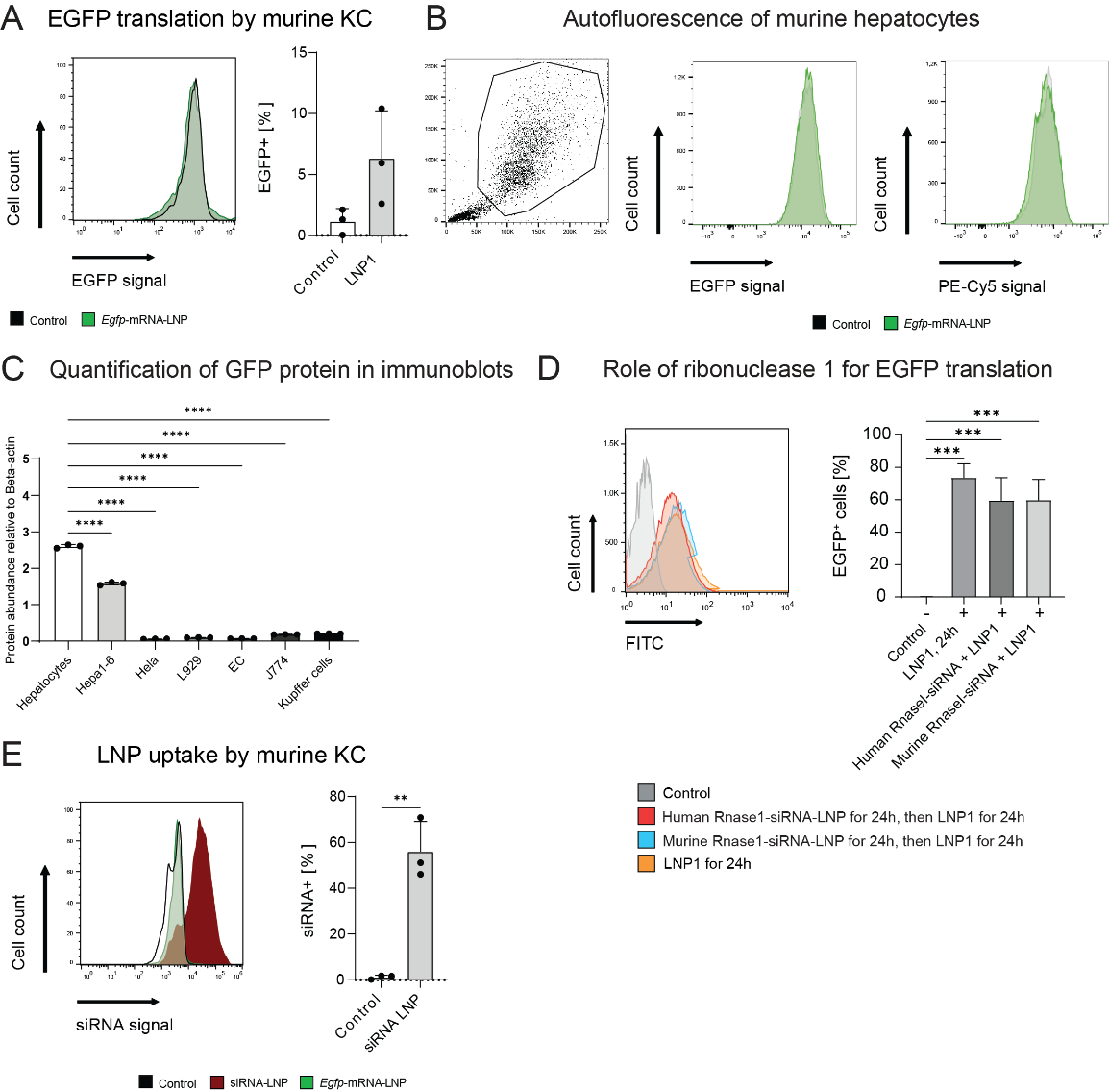
**

**Figure S4: Flow cytometric analysis of uptake and translation of LNP.** (A) Murine CD45^+^F4/80^+^ CD11b^low^ KC were isolated from murine livers and cultured for 24 hours with LNP1. EGFP signals were determined using flow cytometry and shown as overlays. (B) Gating of freshly FACS-isolated hepatocytes showing strong autofluorescence in the EGFP/FITC and PE-Cy5 channel. (C) Quantifications of the EGFP immunoblot of Figure 2B. (D) Impact of ribonuclease inhibition on EGFP translation by human primary macrophages. Human primary macrophages at day 5 of culture were pre-incubated with Dsi-RNA to knock down Ribonuclease 1. After 24 hours, the cells were treated with *Egfp*-mRNA-LNP for another 24 hours and then the cells were studied for EGFP protein expression using flow cytometry. (E) Liver-derived murine KC co-treated with LNP1 (mRNA) and LNP8 (siRNA) show uptake of the siRNA-LNP. Data represent mean of two measurements n = 2 ± SD; *p < 0.05, **p < 0.01, ***p < 0.001 (One-way ANOVA in C and t-test in D).


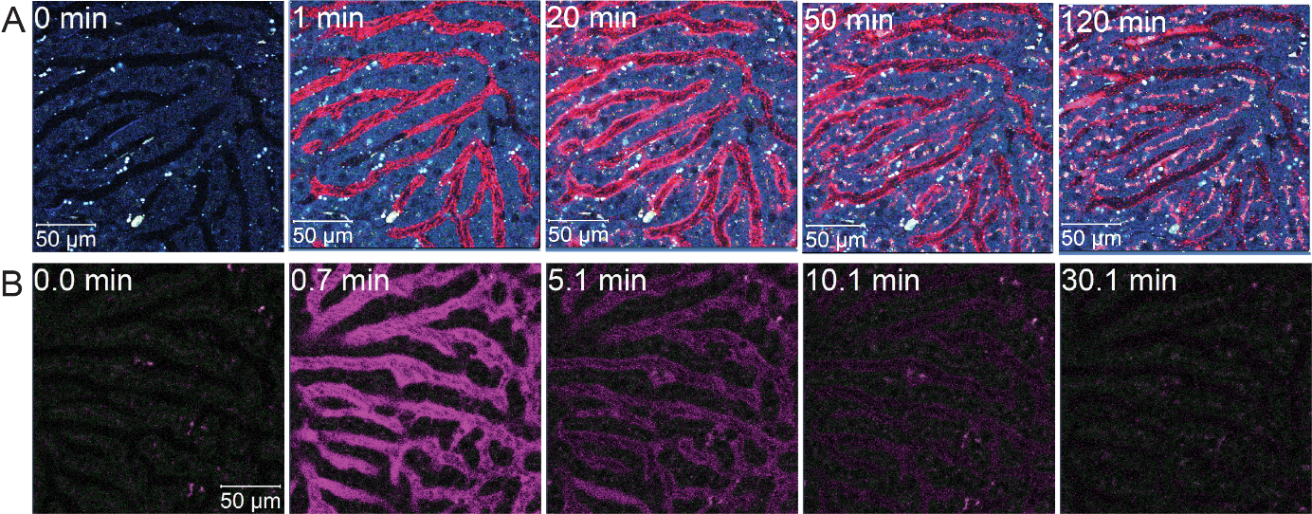


**Figure S5: Intrahepatic distribution of siRNA-LNP and free siRNA.** IVM was used to study the longitudinal distribution directly after intravenous administration of (A) AF594-siRNA-LNP, or (B) free AF647-siRNA.

**Investigation of the mRNA-siRNA conencapsulation**

LNP imaging with confocal microscopy demonstrated that if LNP were first generated separately and then mixed, they appeared also as clear green and red dots. In contrast, in coencapsulated LNP, the red and green signals were rather mixed and less clear (main Figure 4A). Quantifications of colour curves from microscopy demonstrated that the mixed ME LNP also displayed separated colour curves, while the curves were rather closely connected in the LNP obtained from coencapsulation (**Figure S6A**). The AF594-siRNA containing LNP (**Figure S6B**) exhibited similar optical properties as the first version, also confirmed in quantifications thereof (**Figure S6C**). We analysed the mono and co-encapsulation in more detail and evaluated microscopy-derived histograms, quantified the co-encapsulation, and also determined the correlation between both colours. These data suggest that about 80% of the CE LNP are co-encapsulated (**Figure S6D**). Similar observations were made for the AF594 siRNA-LNP (**Figure S6E**).


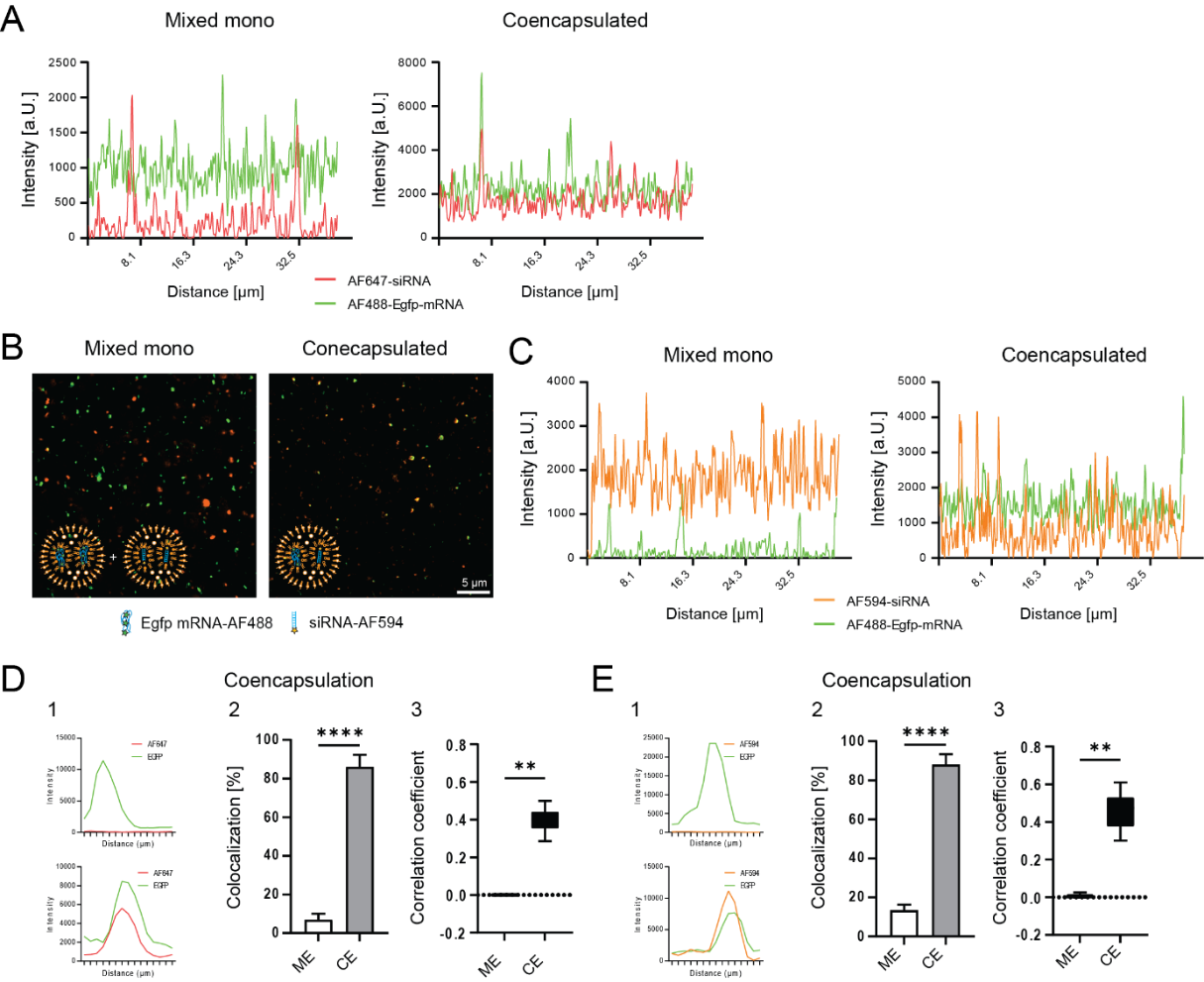


**Figure S6: Analysis of mRNA-siRNA conencapsulation into LNP.** (A) Signals detected in confocal microscopy of mixed monoencapsulated and coencapsulated LNP (AF488-mRNA and AF647-siRNA). (B) Confocal microscopy was done to study the coencapsulation of siRNA-AF594 and AF488mRNA. Monoencapsulated LNP were generated (AF488-labelled Egfp mRNA and AF594-siRNA), mixed and compared to LNP with mRNA and siRNA conencapsulated at a ratio of 2:1 (mRNA:siRNA). (C) AF488-labelled Egfp mRNA-LNP were mixed 1:1 with AF594-siRNA-LNP. (D) Analysis of coencapsulation showing (1) histograms from confocal microscopy, (2) analysis of coencapsulation, (3) Pearson correlation of signals of mixed monoencapsulated vs. coencapsulated LNP. (E) As in E, but AF594-siRNA instead. Data represent mean of at least three measurements n = 3 ± SD; *p < 0.05, **p < 0.01, ***p < 0.001 (One-way ANOVA).


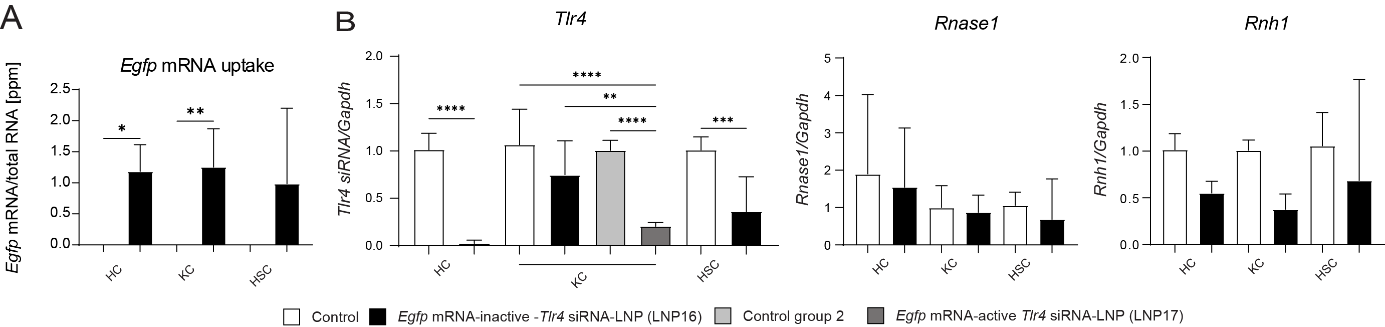


**Figure S7: Knockdown of Tlr4 by LNP with *Egfp* mRNA coencapsulated with inactive Tlr4 siRNA-LNP**. Animals were injected with 3 mg/kg of LNP16 (*Egfp*-mRNA-siRNA-LNP at the RNA weight ratio of 2:1 mRNA:siRNA) and sacrificed 16 hours after the injection of the LNP. Total RNA was isolated and qPCR was done. (A) QPCR-based quantification of the uptake of LNP by relating to the total RNA content of HC, KC, and HSC (*Egfp* mRNA was normalized as per mille of total RNA). (B) After injection of LNP16 (mRNA+NC-siRNA) and LNP17 (mRNA+Tlr4 siRNA), Tlr4, Ribonuclease 1 and Ribonuclease 1 inhibitor (Rhn1) were studied in HC, KC, and HSC. Data represent mean of n = 6 ± SD; *p < 0.05, **p < 0.01, ***p < 0.001 (One-way ANOVA).


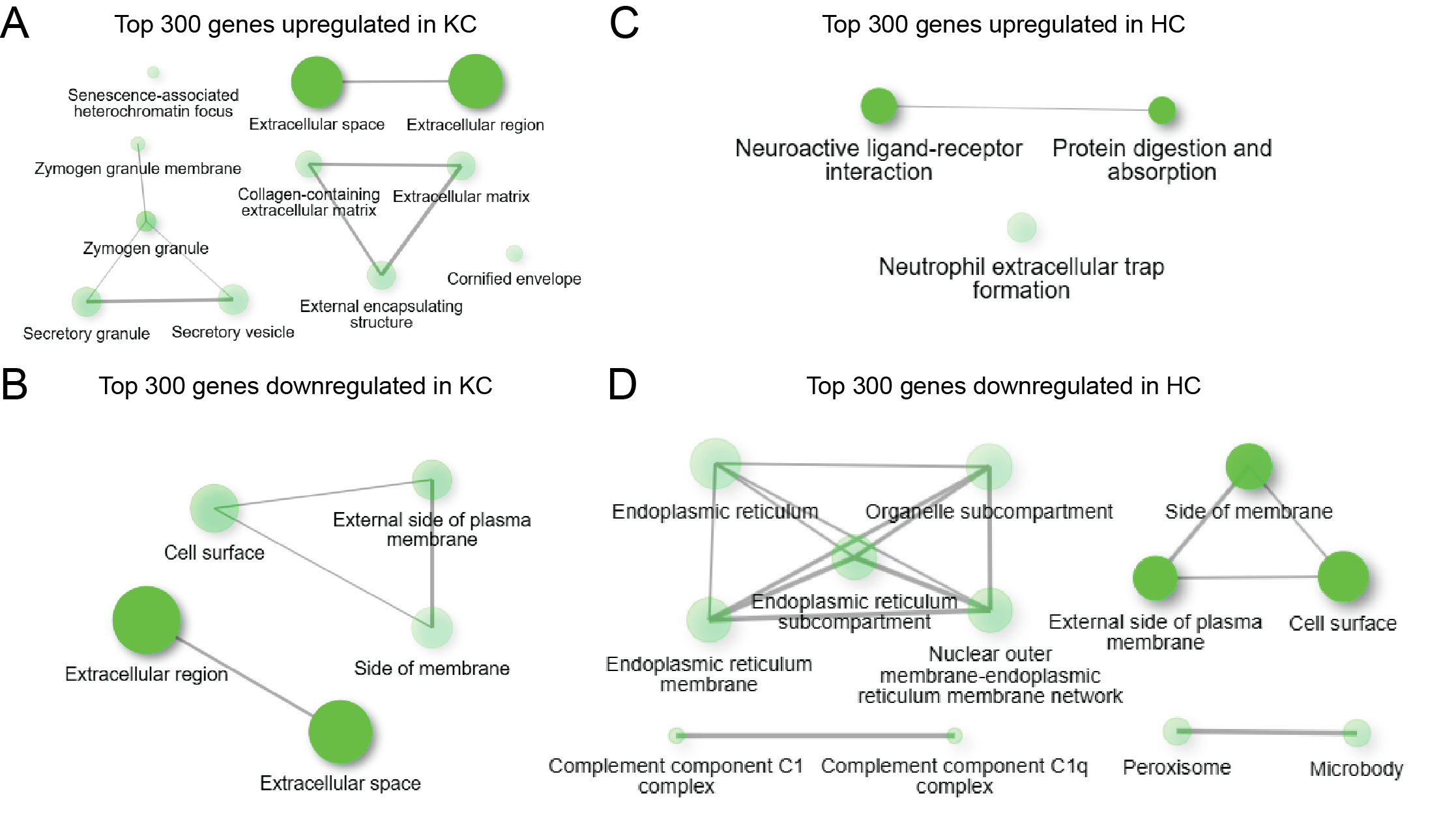


**Figure S8: Analysis of gene enrichment of the 300 most up and downregulated genes in hepatocytes and Kupffer cells *in vivo* after treatment with LNP.** The ShinyGO 0.8 was used with the setting GO term cellular compartment and standard settings. These plot show the relationship between enriched pathways. Two pathways (nodes) are connected if they share 20% (default) or more genes. Nodes were moved for clarity by dragging. Darker nodes are more significantly enriched gene sets. Bigger nodes represent larger gene sets. Thicker edges represent more overlapped genes.

**Expanded discussion and supplementary results on the effects of LNP on gene expression of hepatocytes and Kupffer cells**

Supplementary markers of HC were the *C-reactive protein* (*Crp*) which correlates with the severity of an inflammatory response, and *ATPase Phospholipid Transporting 8B1* (*Atp8b1*), an aminophospholipid-transporting ATPase (**Figure S9A**). It needs to be noted that *mt-Rnr2* and *mt-Cytb* are not protein-coding, but these genes are reflective of the strong involvement of HC in protein and energy production. The human protein atlas confirmed that *Atp8b1* and *Crp* are markers of hepatocytes (www.proteinatlas.org). We found that the protease inhibitors *Serpina 10,* which are important and well-known acute phase proteins (APP), were upregulated in HC. In addition, the carrier protein *Hemopexin* (*Hpx*) and the transport-associated *Orosomucoid 1* (*Orm1*) were upregulated. In addition, the LNP caused statistically significant elevations of the two RNA binding proteins *Interferon induced protein with tetratricopeptide repeats 1* (*Ifit1*), and *Inter-alpha-trypsin inhibitor heavy chain 3* (*Itih3*). Upregulation was also noted for *Haptoglobin* (*Hp*) and an additional APP *Serum amyloid 2* (*Saa2*) (**Figure S9B**). Significant reductions in gene expression of HC by LNP were notably observed for genes encoding proteins involved in coagulation such as *factor VII* and *Plasminogen*. In addition, significantly lower transcripts were detected for *Solute carrier organic anion transporter family member* (*Slco1b2*) that functions in transport of organic anions. Many of the downregulated genes were involved in drug metabolism such as *ATP binding cassette subfamily G member 5* (*Abcg5*), *Sulfotransferase family 2A member 1* (*Sult2a1*), as well as *Aquaporin 8, 9* and *11*. Importantly, the *Polymeric Ig receptor* (*Pigr*) that facilitates transcytotic transport through the hepatocytes, and also the important receptor *Asiasoglycoprotein receptor 1* (*Asgpr1*) that is the entry portal for GalNAc-siRNA, were also downregulated by the LNP (**Figure S9C**).

Additional markers for KC were *Transcobalamin 2* (*Tcn2*), a transport protein for vitamin B12, and also *Serglycin* (*Srgn*), the major secreted proteoglycan of macrophages. *Tcn2* was described in bone marrow and kidney, but not the liver, and *Srgn* was confirmed to be expressed by KC, but also by cholangiocytes, EC, and neutrophils (www.proteinatlas.org) (**Figure S9D**).

In addition, the *lymphocyte antigen 6 family member A* (*Ly6a*), a marker for stemness, and the *Coagulation factor II thrombin receptor* (*F2r*) were significantly upregulated by LNP. Further, the and the RNA binding proteins *Ubiquitin-like protein 15* (*Isg15*) and *Interferon-induced transmembrane protein 3* (*Ifitm3*) were induced in KC by the LNP. Upregulation was also noted for the gene encoding for the secretory protein *TIMP Metallopeptidase Inhibitor 3* (*Timp3*), which inhibits metalloproteinases, peptidases that degrade the extracellular matrix. We further noted downregulation of *Secreted protein acidic and cysteine rich* (*Sparc*) that was linked to hepatic inflammation. The two *Member RAS oncogene family 7* and *11e*, *Rab7* and *Rab11e,* that function in vesicle trafficking, as well as the *Cxc chemokine 10* (*Cxcl10*) which leads to an attraction of monocytes and T cells, and the *Solute carrier family 4 member 2* (*Slc4a2*) that functions in anion exchange (**Figure S9E**).

In KC, the LNP caused downregulation of Selenoprotein mRNA, a gene that encodes a secretory protein that transports Selenium to specific target tissues and exhibits antioxidant characteristics. LNP further led to downregulation of the *Insulin-like growth factor binding protein-7* (*Igfbp7*), that was linked to liver metabolism. Interestingly, In addition, we detected significantly lower levels of the *complement factor 1qa* (*C1qa*), of the *CD5 ligand* (*Cd5l*), an apoptosis inhibitor of macrophages, of *Rab5*, and of *monocyte to macrophage differentiation-associated* (*Mmd*) (**Figure S9F**).

**
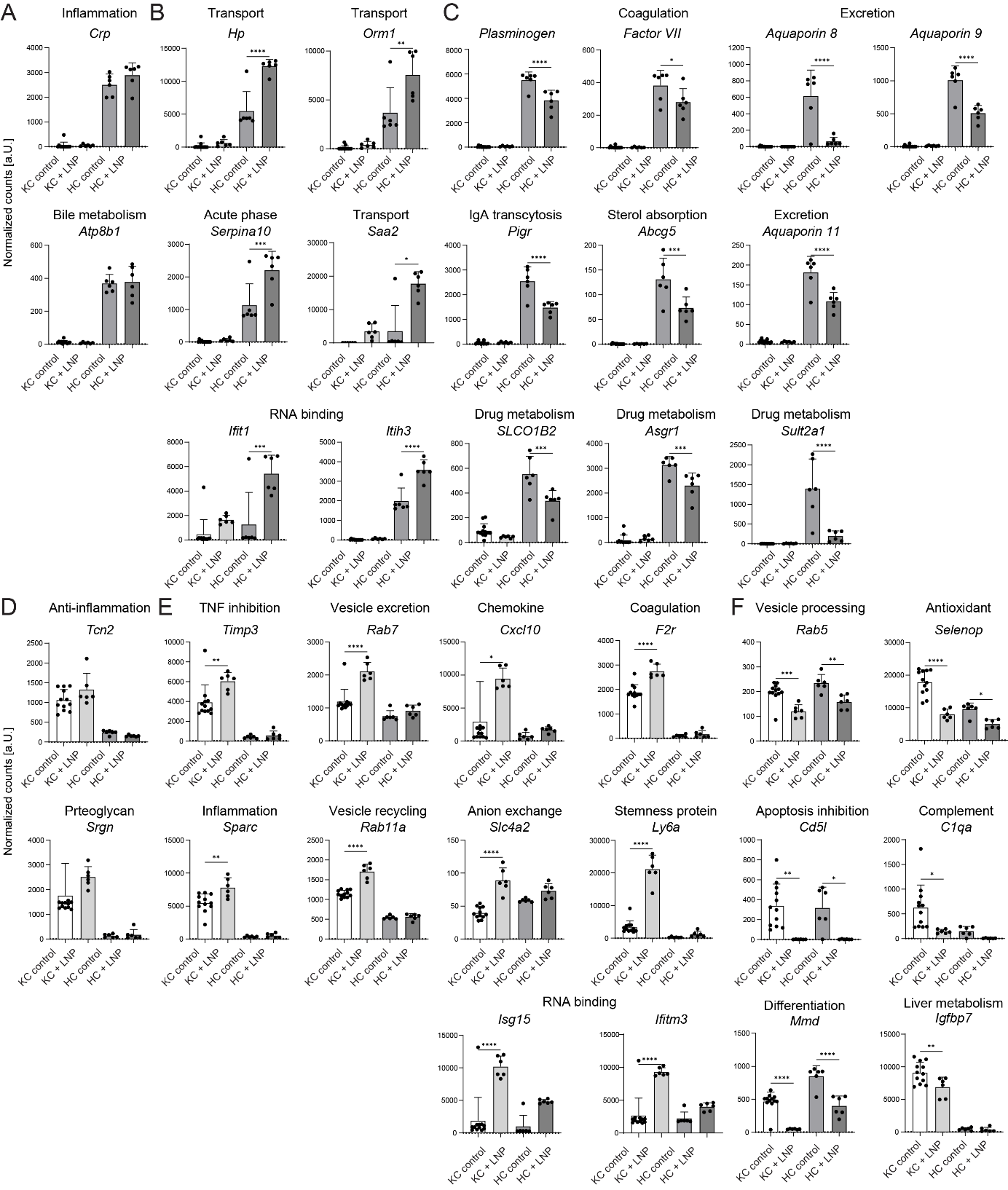
**

**Figure S9: Supplementary genes affected by LNP in hepatocytes and Kupffer cells.** Total RNA was purified from hepatocytes and Kupffer cells treated as described in Figure 5 and sequenced. (A) Marker genes hepatocytes of hepatocytes unaffected by LNP. (B) genes induced in hepatocytes by LNP. (C) Genes down-regulated by LNP in hepatocytes. (D) KC-specific genes, (E) genes upregulated in KC by LNP, and (F) genes down-regulated in KC by LNP. Data represent mean of n = 6-12 ± SD; *p < 0.05, **p < 0.01, ***p < 0.001 (One-way ANOVA).

**
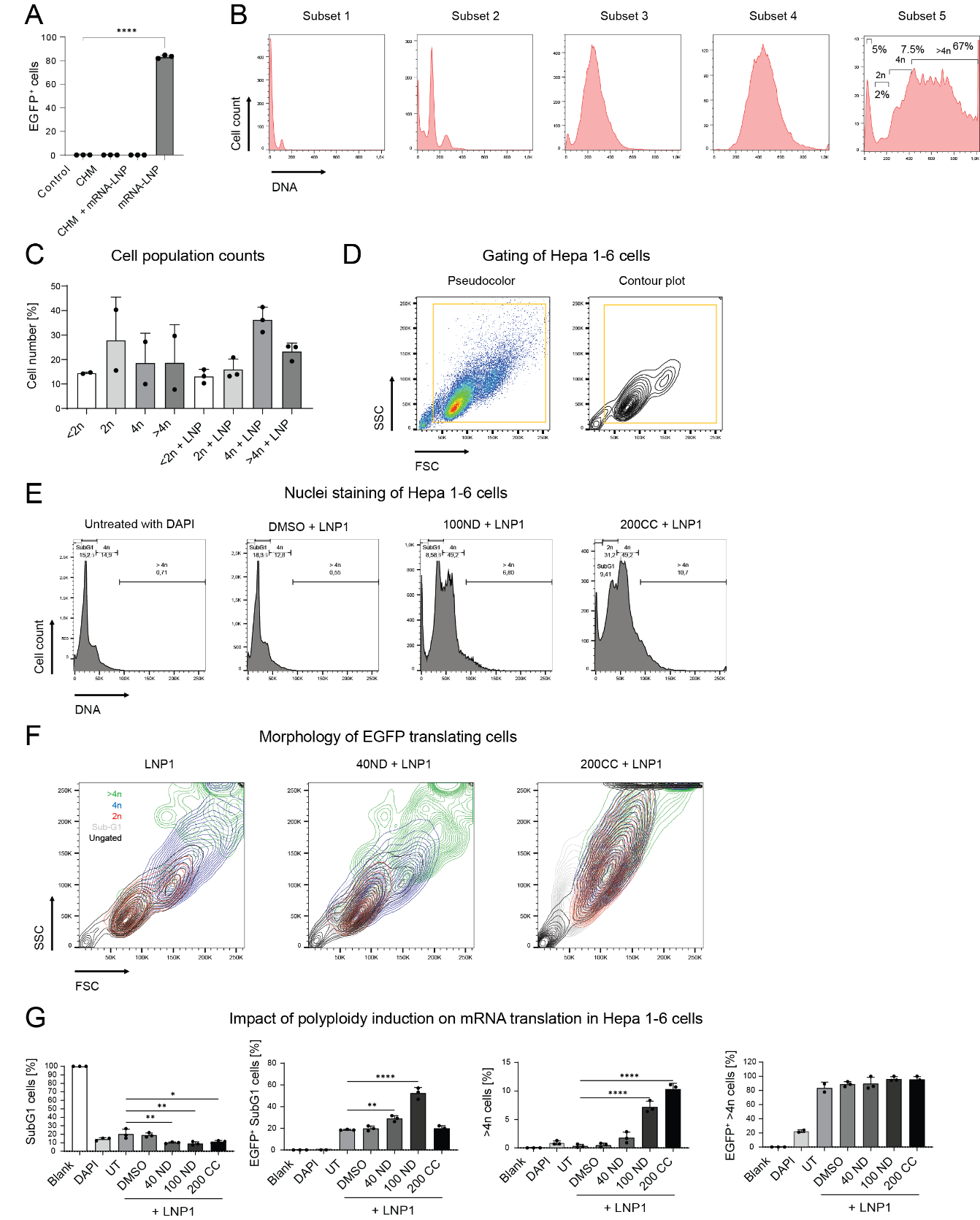
**

**Figure S10: Factors affecting translation of mRNA-LNP.** (A) HeLa cells were either left untreated, were treated with cycloheximide, were treated with cycloheximide and 1 µg/mL LNP1, or were transfected with LNP1 only. (B) Flow cytometric analysis of the DNA content of hepatocytes that were transfected with LNP1. (C) Cell numbers determined for the different degrees of ploidy for cells with stained nuclei or cells stained for nuclei and transfected with Egfp mRNA-LNP. (D) Gating strategy for Hepa 1-6 cells and comparison of Pseudocolor and contour plots, orange cells are selected for subsequent analysis using (E) nucleic staining using DAPI where the different stages of cell cycle progression are analysed. (F) Morphology of cells expressing EGFP at different ploidy levels. (G) Impact of polyploidy induction on the cell cycle and EGFP translation by the different cellular subsets. Data represent mean of n = 2-3 ± SD; *p < 0.05, **p < 0.01, ***p < 0.001 (One-way ANOVA).

**Details on the *in vitro* transcription for synthesis of mRNA**

The *in vitro* transcriptions were set up as following using the HiScribe® T7 High Yield RNA Synthesis Kit (New England Biolabs) for the AF-488 labeled RNA or HiScribe® T7 mRNA Kit with CleanCap® Reagent AG (New England Biolabs) for the mRNA and AF647 labeled mRNA with an amended synthesis:

|  | AF-488 RNA | mRNA | AF-647 mRNA |
| --- | --- | --- | --- |
| 10x Reaction Buffer (uL) | 4 | 0 | 0 |
| 10x Clean Cap AG Reaction Buffer (uL) | 0 | 4 | 4 |
| ATP (uL) | 4 | 4 | 4 |
| CTP (uL) | 3 | 4 | 3 |
| GTP (uL) | 4 | 4 | 4 |
| UTP (uL) | 4 | 4 | 4 |
| Clean Cap AG (uL) | 0 | 4 | 4 |
| CTP-PEG5-AF488 (uL) | 10 | 0 | 0 |
| CTP-PEG5-AF647(uL) | 0 | 0 | 10 |
| DEPC water (uL) | 3 | 8 | 0 |
| Ribo Lock RNase Inhibitor  (uL, c = 40 U/uL)) | 2 | 2 | 2 |
| DNA template linearized (uL, c = 1 ug/uL) | 2 | 2 | 2 |
| T7 Polymerase (uL) | 4 | 4 | 4 |

All reaction mixtures were briefly vortexed and incubated at 37 °C for 4 h.

Following, 2U DNase I from the HiScribe® T7 High Yield RNA Synthesis Kit (New England Biolabs) were added to the solution followed by brief vortexing and incubation at 37 °C for another 30 min. The RNA was purified from the reaction mixture using the Monarch® RNA Cleanup Kit (500 μg) (New England Biolabs). The obtained RNA was eluted from the columns with a 0.1 M Acetic Acid solution (pH = 4). Concentration and purity of the obtained RNA were assessed via UV/Vis (Thermo Scientific, Nanodrop One) and a denaturing Agarose Gel Electrophoresis (0,8%, 90 V, 80 min).

**DNA Template generation**: The sequence of the DNA Template used for the fabrication of all three RNA molecules is shown down below. The sequence contains the UTRs from the BioNTech/Pfizer covid vaccines [1,2,3] and encodes for eGFP. Additionally, the DNA template encodes a 2x60 nt long Poly(A) tail right after the 3’UTR. The template was amplified in a pJET 1.2 (Thermo Scientific) plasmid using the *e.coli* strain StbI4 (Invitrogen) and isolated from 150 ml bacterial culture in TB media with the GenElute™ HP Plasmid Maxiprep Kit (Sigma-Aldrich). The plasmid was digested with NotI and XbaI followed by separation of the two resulting fragments on an Agarose Gel Electrophoresis (0,8%, 90 V, 80 min). The band of interest was cut from the agarose gel, purified via the Monarch® DNA Gel Extraction Kit (New England Biolabs) and quantified via UV/Vis (Thermo Scientific, Nanodrop One).

DNA template for IVT:

TAATACGACTCACTATAAGGAATAAACTAGTATTCTTCTGGTCCCCACAGACTCAGAGAGAACCCGCCACCATGGTTTCCAAAGGCGAAGAACTGTTCACGGGCGTGGTTCCTATCTTGGTAGAATTGGATGGGGACGTTAACGGTCACAAGTTTAGCGTGTCAGGCGAGGGAGAGGGCGATGCAACGTATGGGAAGCTCACACTGAAATTTATTTGTACGACAGGTAAATTGCCCGTTCCCTGGCCCACGCTCGTGACGACACTGACGTATGGCGTGCAATGTTTCTCACGCTACCCTGACCACATGAAACAGCACGATTTCTTTAAATCCGCAATGCCGGAGGGTTACGTGCAGGAACGGACCATCTTCTTCAAAGATGATGGAAACTACAAGACACGCGCTGAAGTCAAGTTCGAGGGCGATACTCTCGTTAACCGCATTGAACTTAAAGGGATCGATTTTAAGGAGGACGGTAATATACTGGGTCACAAACTGGAGTATAACTACAACAGCCATAACGTCTATATCATGGCAGATAAGCAGAAGAATGGAATTAAAGTAAATTTCAAAATACGGCATAACATCGAAGACGGGTCTGTCCAGCTTGCAGACCATTATCAACAGAATACGCCTATAGGCGATGGGCCGGTCCTGCTCCCCGACAATCACTACTTGTCTACACAGTCCGCCCTGTCCAAGGACCCGAACGAGAAGCGGGATCACATGGTACTGTTGGAGTTCGTAACTGCCGCTGGCATCACACTGGGGATGGATGAACTGTACAAATAACTCGAGCTGGTACTGCATGCACGCAATGCTAGCTGCCCCTTTCCCGTCCTGGGTACCCCGAGTCTCCCCCGACCTCGGGTCCCAGGTATGCTCCCACCTCCACCTGCCCCACTCACCACCTCTGCTAGTTCCAGACACCTCCCAAGCACGCAGCAATGCAGCTCAAAACGCTTAGCCTAGCCACACCCCCACGGGAAACAGCAGTGATTAACCTTTAGCAATAAACGAAAGTTTAACTAAGCTATACTAACCCCAGGGTTGGTCAATTTCGTGCCAGCCACACCCTGGAGCTAGCTAGCCATATGAAAAAAAAAAAAAAAAAAAAAAAAAAAAAAAAAAAAAAAAAAAAAAAAAAATGCATAAAAAAAAAAAAAAAAAAAAAAAAAAAAAAAAAAAAAAAAAAAAAAAAAAGAATTC CTAGCATAACCCCTTGGGGCCTCTAAACGGGTCTTGAGGGGTTTTTTG

T7 Promotor, 5’ UTR from BioNTech, 3’UTR from BioNTech, Poly(A) side, T7 Terminator

**Table S6: The 300 most highly upregulated genes in Kupffer cells after 16 hours of treatment with LNP.**

| ENSMUSG00000071356.8 | *Reg3b* |
| --- | --- |
| ENSMUSG00000013523.14 | *Bcas1* |
| ENSMUSG00000083079.5 | *Amy2b* |
| ENSMUSG00000036938.18 | *Try5* |
| ENSMUSG00000044903.16 | *Psg22* |
| ENSMUSG00000079516.3 | *Reg3a* |
| ENSMUSG00000037390.14 | *Muc3* |
| ENSMUSG00000054446.9 | *Cpa1* |
| ENSMUSG00000038508.8 | *Gdf15* |
| ENSMUSG00000033196.18 | *Myh2* |
| ENSMUSG00000029522.13 | *Pla2g1b* |
| ENSMUSG00000096569.3 | *Amy2a2* |
| ENSMUSG00000090230.3 | *Gm16315* |
| ENSMUSG00000059654.8 | *Reg1* |
| ENSMUSG00000074344.8 | *Tmigd3* |
| ENSMUSG00000116836.2 | *Gm49727* |
| ENSMUSG00000054106.8 | *Try4* |
| ENSMUSG00000078520.4 | *Cela3a* |
| ENSMUSG00000031957.7 | *Ctrb1* |
| ENSMUSG00000071553.11 | *Cpa2* |
| ENSMUSG00000086527.2 | *Gm15856* |
| ENSMUSG00000002289.17 | *Angptl4* |
| ENSMUSG00000093931.3 | *Amy2a3* |
| ENSMUSG00000022501.7 | *Prm1* |
| ENSMUSG00000074268.5 | *Amy2a5* |
| ENSMUSG00000103017.2 | *Gm37505* |
| ENSMUSG00000103373.2 | *Gm37238* |
| ENSMUSG00000086327.3 | *Slfn5os* |
| ENSMUSG00000050147.10 | *F2rl3* |
| ENSMUSG00000030137.9 | *Tuba8* |
| ENSMUSG00000115727.2 | *4930432J09Rik* |
| ENSMUSG00000094392.2 | *Gm3788* |
| ENSMUSG00000068893.6 | *Sprr2a2* |
| ENSMUSG00000089827.2 | *1700023H06Rik* |
| ENSMUSG00000023433.9 | *Cela3b* |
| ENSMUSG00000049939.7 | *Lrrc4* |
| ENSMUSG00000032128.16 | *Robo3* |
| ENSMUSG00000071517.7 | *Gm10334* |
| ENSMUSG00000083606.4 | *Gm15916* |
| ENSMUSG00000096770.3 | *Amy2a4* |
| ENSMUSG00000057163.4 | *Prss2* |
| ENSMUSG00000060317.10 | *Acnat2* |
| ENSMUSG00000062380.5 | *Tubb3* |
| ENSMUSG00000026818.6 | *Cel* |
| ENSMUSG00000039084.9 | *Chad* |
| ENSMUSG00000030954.12 | *Gp2* |
| ENSMUSG00000107448.2 | *Gm43947* |
| ENSMUSG00002075770.1 | *Gm56298* |
| ENSMUSG00000110310.2 | *4930518J21Rik* |
| ENSMUSG00000040660.7 | *Cyp2b9* |
| ENSMUSG00000065721.3 | *Gm26168* |
| ENSMUSG00000042179.7 | *Pnliprp1* |
| ENSMUSG00000004885.6 | *Crabp2* |
| ENSMUSG00000100396.2 | *Gm29367* |
| ENSMUSG00000084607.3 | *Gm22208* |
| ENSMUSG00000030623.5 | *Prss23os* |
| ENSMUSG00000086213.3 | *A330040F15Rik* |
| ENSMUSG00000042333.17 | *Tnfrsf14* |
| ENSMUSG00000025091.5 | *Pnliprp2* |
| ENSMUSG00000108614.2 | *2610306O10Rik* |
| ENSMUSG00000120031.1 | *Gm32542* |
| ENSMUSG00000053980.5 | *Gm9930* |
| ENSMUSG00000034040.18 | *Galnt17* |
| ENSMUSG00000095686.8 | *Gm3099* |
| ENSMUSG00000085327.3 | *Gm16104* |
| ENSMUSG00000078664.3 | *Sprr2a1* |
| ENSMUSG00000094588.3 | *Or8c20* |
| ENSMUSG00000053615.2 | *Gm9913* |
| ENSMUSG00000025905.15 | *Oprk1* |
| ENSMUSG00000075552.5 | *Cyp3a41b* |
| ENSMUSG00000107898.2 | *Gm53051* |
| ENSMUSG00000011463.6 | *Cpb1* |
| ENSMUSG00000104781.2 | *Gm43303* |
| ENSMUSG00000064307.14 | *Lrrc51* |
| ENSMUSG00000029882.6 | *Prss3b* |
| ENSMUSG00000051839.8 | *Gypa* |
| ENSMUSG00000074109.5 | *Mrgprx2* |
| ENSMUSG00000038086.5 | *Hspb2* |
| ENSMUSG00000095195.9 | *Gm3005* |
| ENSMUSG00000075551.5 | *Cyp3a41a* |
| ENSMUSG00000024245.5 | *Tmem178* |
| ENSMUSG00000027202.13 | *Slc12a1* |
| ENSMUSG00000114053.3 | *Gm35330* |
| ENSMUSG00000027528.13 | *Fabp9* |
| ENSMUSG00000024786.10 | *Majin* |
| ENSMUSG00000090346.3 | *Vmn1r5* |
| ENSMUSG00000053522.12 | *Lgals7* |
| ENSMUSG00000037583.3 | *Nr0b2* |
| ENSMUSG00000120841.1 | *Gm56542* |
| ENSMUSG00002076484.1 | *Gm54995* |
| ENSMUSG00000094520.5 | *Or51q1* |
| ENSMUSG00000078706.6 | *Gm53* |
| ENSMUSG00000030226.13 | *Lmo3* |
| ENSMUSG00000106005.2 | *Gm29151* |
| ENSMUSG00000024503.4 | *Spink1* |
| ENSMUSG00000053442.5 | *4930597O21Rik* |
| ENSMUSG00000099536.2 | *Gm9569* |
| ENSMUSG00000091472.4 | *Gm3739* |
| ENSMUSG00000109517.2 | *Gm44763* |
| ENSMUSG00000028359.5 | *Orm3* |
| ENSMUSG00000085067.4 | *Gm15631* |
| ENSMUSG00000079507.11 | *H2-Q1* |
| ENSMUSG00000086665.3 | *Gm13067* |
| ENSMUSG00000019996.18 | *Map7* |
| ENSMUSG00000029337.3 | *Fgf5* |
| ENSMUSG00000058579.6 | *Cela2a* |
| ENSMUSG00000121190.1 | *Gm56997* |
| ENSMUSG00000078955.4 | *Gm14222* |
| ENSMUSG00000105785.2 | *Gm42526* |
| ENSMUSG00000106976.2 | *Gm42963* |
| ENSMUSG00000048806.5 | *Ifnb1* |
| ENSMUSG00000120853.1 | *Gm57216* |
| ENSMUSG00000068606.7 | *Gm4841* |
| ENSMUSG00000004791.8 | *Pgf* |
| ENSMUSG00000070427.5 | *Il18bp* |
| ENSMUSG00000020722.6 | *Cacng1* |
| ENSMUSG00000049350.7 | *Zg16* |
| ENSMUSG00000065822.3 | *Snord15a* |
| ENSMUSG00000066319.7 | *Rtp3* |
| ENSMUSG00000120648.1 | *Gm56935* |
| ENSMUSG00000074115.6 | *Saa1* |
| ENSMUSG00000064382.3 | *Gm26447* |
| ENSMUSG00000089744.3 | *Gm16146* |
| ENSMUSG00000117269.2 | *Gm9706* |
| ENSMUSG00000086775.8 | *Snhg7os* |
| ENSMUSG00000036766.13 | *Dner* |
| ENSMUSG00000046008.9 | *Pnlip* |
| ENSMUSG00000034394.15 | *Lif* |
| ENSMUSG00000092008.3 | *Cyp2c69* |
| ENSMUSG00000079523.9 | *Tmsb10* |
| ENSMUSG00000104675.3 | *Gm43689* |
| ENSMUSG00000086298.3 | *Gm11716* |
| ENSMUSG00000020475.4 | *Pgam2* |
| ENSMUSG00000057465.6 | *Saa2* |
| ENSMUSG00000064637.3 | *Snora20* |
| ENSMUSG00000024857.17 | *Cabp2* |
| ENSMUSG00000073652.11 | *Apol7d* |
| ENSMUSG00000100180.2 | *Gm28140* |
| ENSMUSG00000084797.2 | *Gm14321* |
| ENSMUSG00000089834.3 | *Gm16303* |
| ENSMUSG00000023905.16 | *Tnfrsf12a* |
| ENSMUSG00000045591.7 | *Olig3* |
| ENSMUSG00000000392.18 | *Fap* |
| ENSMUSG00000000204.17 | *Slfn4* |
| ENSMUSG00000025351.15 | *Cd63* |
| ENSMUSG00000037239.9 | *Spred3* |
| ENSMUSG00000023249.16 | *Parp3* |
| ENSMUSG00000020787.15 | *P2rx1* |
| ENSMUSG00000039329.9 | *Tex19.1* |
| ENSMUSG00000041449.19 | *Serpina3h* |
| ENSMUSG00000119521.1 | *n-R5s123* |
| ENSMUSG00000023403.15 | *Stk31* |
| ENSMUSG00000026204.16 | *Ptprn* |
| ENSMUSG00000055704.5 | *Gm9978* |
| ENSMUSG00000038060.16 | *Dlec1* |
| ENSMUSG00000000440.13 | *Pparg* |
| ENSMUSG00000002847.8 | *Pla1a* |
| ENSMUSG00000061540.4 | *Orm2* |
| ENSMUSG00000116725.2 | *Gm29686* |
| ENSMUSG00000086000.3 | *Gm12493* |
| ENSMUSG00000103006.2 | *4933417C20Rik* |
| ENSMUSG00000090708.2 | *Gm17196* |
| ENSMUSG00000040583.9 | *Cyp2b13* |
| ENSMUSG00000090942.2 | *F830016B08Rik* |
| ENSMUSG00000120312.1 | *Gm57403* |
| ENSMUSG00000026435.16 | *Slc45a3* |
| ENSMUSG00000110322.2 | *Gm8110* |
| ENSMUSG00000111003.2 | *Gm47122* |
| ENSMUSG00000006179.10 | *Prss16* |
| ENSMUSG00000085479.2 | *9430073C21Rik* |
| ENSMUSG00000040600.10 | *Eps8l3* |
| ENSMUSG00000111713.3 | *Gm20234* |
| ENSMUSG00000105328.2 | *Gm43180* |
| ENSMUSG00000059659.9 | *Gm10069* |
| ENSMUSG00000030107.11 | *Usp18* |
| ENSMUSG00000050366.7 | *Or6b13* |
| ENSMUSG00000097746.3 | *Gm6225* |
| ENSMUSG00000023224.13 | *Serping1* |
| ENSMUSG00000112384.2 | *Gm34921* |
| ENSMUSG00000039021.16 | *Ttc16* |
| ENSMUSG00000074637.8 | *Sox2* |
| ENSMUSG00000035896.6 | *Rnase1* |
| ENSMUSG00000091955.3 | *Tmsb10b* |
| ENSMUSG00000056155.7 | *Nanos3* |
| ENSMUSG00000047517.14 | *Dmbt1* |
| ENSMUSG00000109713.2 | *Pvrig* |
| ENSMUSG00000022894.7 | *Adamts5* |
| ENSMUSG00000030361.18 | *Klrb1a* |
| ENSMUSG00000021278.8 | *Amn* |
| ENSMUSG00000092349.2 | *Smim40* |
| ENSMUSG00000031896.8 | *Ctrl* |
| ENSMUSG00000024575.17 | *Pde6a* |
| ENSMUSG00000073430.7 | *Gm10505* |
| ENSMUSG00000066672.3 | *Or10x1* |
| ENSMUSG00000090298.3 | *Sult3a2* |
| ENSMUSG00000110273.2 | *Gm41231* |
| ENSMUSG00000117393.3 | *Gm36279* |
| ENSMUSG00000078349.5 | *AW011738* |
| ENSMUSG00000029092.10 | *D5Ertd615e* |
| ENSMUSG00000107163.2 | *Gm43496* |
| ENSMUSG00000092021.10 | *Gbp11* |
| ENSMUSG00000078853.9 | *Igtp* |
| ENSMUSG00000084846.2 | *A730011C13Rik* |
| ENSMUSG00000106673.2 | *Gm43374* |
| ENSMUSG00000074489.10 | *Bglap3* |
| ENSMUSG00000045780.4 | *Or51v8* |
| ENSMUSG00000086205.2 | *Gm12679* |
| ENSMUSG00000007030.9 | *Vwa7* |
| ENSMUSG00000028699.10 | *Tspan1* |
| ENSMUSG00000079173.12 | *Zan* |
| ENSMUSG00000087273.2 | *Gm13203* |
| ENSMUSG00000040035.15 | *Disp2* |
| ENSMUSG00000102200.2 | *Gm36957* |
| ENSMUSG00000047988.2 | *4933428G20Rik* |
| ENSMUSG00000117516.2 | *Gm18089* |
| ENSMUSG00000102914.2 | *Gm35106* |
| ENSMUSG00000001507.17 | *Itga3* |
| ENSMUSG00000005667.9 | *Mthfd2* |
| ENSMUSG00000034258.5 | *Flvcr2* |
| ENSMUSG00000050014.9 | *Apol10b* |
| ENSMUSG00000068246.7 | *Apol9b* |
| ENSMUSG00000103827.3 | *Gm32950* |
| ENSMUSG00000050092.4 | *Sprr2b* |
| ENSMUSG00000105804.2 | *Gm43654* |
| ENSMUSG00000054589.3 | *Gm9949* |
| ENSMUSG00000092368.3 | *A930015D03Rik* |
| ENSMUSG00000046711.17 | *Hmga1* |
| ENSMUSG00000030713.7 | *Klk7* |
| ENSMUSG00000001672.15 | *Marveld3* |
| ENSMUSG00000065228.3 | *Gm25789* |
| ENSMUSG00000104545.2 | *E030032P16Rik* |
| ENSMUSG00000102801.2 | *Gm37478* |
| ENSMUSG00000114629.2 | *Gm48244* |
| ENSMUSG00000092500.2 | *Gm20400* |
| ENSMUSG00000015653.14 | *Steap2* |
| ENSMUSG00000078249.6 | *Hmga1b* |
| ENSMUSG00000086695.2 | *Gm15247* |
| ENSMUSG00000066861.15 | *Oas1g* |
| ENSMUSG00000117796.2 | *Gm35406* |
| ENSMUSG00000118591.2 | *Gm52955* |
| ENSMUSG00000120098.1 | *Gm57432* |
| ENSMUSG00000091144.3 | *Phf11c* |
| ENSMUSG00000078507.2 | *Aadacl3* |
| ENSMUSG00000040966.11 | *Slc22a2* |
| ENSMUSG00000058163.15 | *Gm5431* |
| ENSMUSG00000073144.6 | *4930599N23Rik* |
| ENSMUSG00000024225.6 | *Clps* |
| ENSMUSG00000106115.2 | *Gm43420* |
| ENSMUSG00000047822.9 | *Angptl8* |
| ENSMUSG00000091756.3 | *Gm3095* |
| ENSMUSG00000107997.2 | *Gm44243* |
| ENSMUSG00000079491.11 | *H2-T10* |
| ENSMUSG00000120905.1 | *Gm56623* |
| ENSMUSG00000104612.2 | *Gm42449* |
| ENSMUSG00000054417.6 | *Cyp3a44* |
| ENSMUSG00000095366.3 | *Gm21860* |
| ENSMUSG00000072769.8 | *Gm10419* |
| ENSMUSG00000121398.1 | *Slfn10-ps* |
| ENSMUSG00000008734.10 | *Gprc5b* |
| ENSMUSG00000063388.6 | *BC023105* |
| ENSMUSG00000096822.3 | *Or1j15* |
| ENSMUSG00000092511.8 | *Gm20547* |
| ENSMUSG00000103580.2 | *Gm10417* |
| ENSMUSG00000109136.2 | *Gm45114* |
| ENSMUSG00000087424.3 | *5730405O15Rik* |
| ENSMUSG00000107734.2 | *Gm30055* |
| ENSMUSG00000023387.9 | *Kcnk16* |
| ENSMUSG00000078117.3 | *Gm16485* |
| ENSMUSG00000103513.2 | *Gm34780* |
| ENSMUSG00000023341.17 | *Mx2* |
| ENSMUSG00000092564.4 | *BC051226* |
| ENSMUSG00000055110.9 | *A630012P03Rik* |
| ENSMUSG00000085939.4 | *Cd63-ps* |
| ENSMUSG00000007888.16 | *Crlf1* |
| ENSMUSG00000087477.3 | *Gm13822* |
| ENSMUSG00000056508.7 | *1700001K19Rik* |
| ENSMUSG00000044734.17 | *Serpinb1a* |
| ENSMUSG00000044125.8 | *9530080O11Rik* |
| ENSMUSG00000069733.12 | *Ube2u* |
| ENSMUSG00000021335.14 | *Slc17a1* |
| ENSMUSG00000029752.13 | *Asns* |
| ENSMUSG00000095681.9 | *Gm8281* |
| ENSMUSG00000001739.15 | *Cldn15* |
| ENSMUSG00000071679.5 | *Rtl4* |
| ENSMUSG00000071001.5 | *Hrct1* |
| ENSMUSG00000033707.10 | *Lrrc24* |
| ENSMUSG00000029556.13 | *Hnf1a* |
| ENSMUSG00000120282.1 | *Gm56738* |
| ENSMUSG00000096546.3 | *Smlr1* |
| ENSMUSG00000037126.17 | *Psd* |
| ENSMUSG00000119414.1 | *n-R5s144* |
| ENSMUSG00000020461.11 | *Clhc1* |
| ENSMUSG00000029322.13 | *Plac8* |
| ENSMUSG00000110481.2 | *Gm45705* |
| ENSMUSG00000103756.2 | *Gm37285* |
| ENSMUSG00000090125.4 | *Pou3f1* |
| ENSMUSG00000074254.5 | *Cyp2a4* |
| ENSMUSG00000057265.14 | *Bbof1* |
| ENSMUSG00000039236.19 | *Isg20* |
| ENSMUSG00000035852.12 | *Misp* |

**Table S7: The 300 most highly upregulated genes in hepatocytes after 16 hours of treatment with LNP.**

| ENSMUSG00000044485.5 | *Klk1b11* |
| --- | --- |
| ENSMUSG00000065872.3 | *Gm25681* |
| ENSMUSG00000079516.3 | *Reg3a* |
| ENSMUSG00000105975.5 | *Gm9831* |
| ENSMUSG00000061780.7 | *Cfd* |
| ENSMUSG00000039720.8 | *Got1l1* |
| ENSMUSG00000044903.16 | *Psg22* |
| ENSMUSG00000078664.3 | *Sprr2a1* |
| ENSMUSG00000074444.5 | *Defa30* |
| ENSMUSG00000103593.2 | *Gm37352* |
| ENSMUSG00000030017.3 | *Reg3g* |
| ENSMUSG00000085478.2 | *Gm11851* |
| ENSMUSG00000121047.1 | *Gm57221* |
| ENSMUSG00000064408.3 | *Gm23924* |
| ENSMUSG00000105584.2 | *Gm42588* |
| ENSMUSG00000119019.1 | *Gm24968* |
| ENSMUSG00000085415.2 | *Selenok-ps1* |
| ENSMUSG00000109510.2 | *Gm42417* |
| ENSMUSG00000023140.5 | *Reg2* |
| ENSMUSG00000030069.16 | *Prok2* |
| ENSMUSG00000102742.2 | *Pcdhga11* |
| ENSMUSG00000065239.3 | *Gm22971* |
| ENSMUSG00000095366.3 | *Gm21860* |
| ENSMUSG00000030703.9 | *Gdpd3* |
| ENSMUSG00000000204.17 | *Slfn4* |
| ENSMUSG00000111713.3 | *Gm20234* |
| ENSMUSG00000109685.2 | *Gvin-ps1* |
| ENSMUSG00000119040.1 | *Gm22044* |
| ENSMUSG00000117128.2 | *Gm49915* |
| ENSMUSG00000120965.1 | *Gm57005* |
| ENSMUSG00000074446.4 | *Defa23* |
| ENSMUSG00000071356.8 | *Reg3b* |
| ENSMUSG00000116725.2 | *Gm29686* |
| ENSMUSG00000106884.2 | *Gm20156* |
| ENSMUSG00000058119.7 | *Gm5771* |
| ENSMUSG00000114414.2 | *A930014D07Rik* |
| ENSMUSG00000118746.1 | *Gm24389* |
| ENSMUSG00000078598.12 | *Skint5* |
| ENSMUSG00000041831.18 | *Sytl3* |
| ENSMUSG00000027876.5 | *Reg4* |
| ENSMUSG00000120651.1 | *Gm31828* |
| ENSMUSG00000109440.3 | *Bc1-ps1* |
| ENSMUSG00000085609.3 | *1700016P03Rik* |
| ENSMUSG00000033368.9 | *Trim69* |
| ENSMUSG00000103138.2 | *Gm2238* |
| ENSMUSG00000068341.9 | *Reg3d* |
| ENSMUSG00000118698.1 | *Gm25890* |
| ENSMUSG00000101132.3 | *Gm8000* |
| ENSMUSG00000062028.9 | *Irgc1* |
| ENSMUSG00000116079.2 | *Gm36245* |
| ENSMUSG00000044103.5 | *Il36g* |
| ENSMUSG00000093979.9 | *Gm2237* |
| ENSMUSG00000036395.16 | *Glb1l2* |
| ENSMUSG00000028545.14 | *Bend5* |
| ENSMUSG00000079852.5 | *Klra4* |
| ENSMUSG00000119213.1 | *Gm25935* |
| ENSMUSG00000087522.4 | *Gm371* |
| ENSMUSG00000056054.10 | *S100a8* |
| ENSMUSG00000056071.13 | *S100a9* |
| ENSMUSG00000096596.3 | *Gm10591* |
| ENSMUSG00000097336.9 | *Fendrr* |
| ENSMUSG00000110894.2 | *Gm48335* |
| ENSMUSG00000068893.6 | *Sprr2a2* |
| ENSMUSG00000086507.2 | *Adap2os* |
| ENSMUSG00000108694.2 | *Gm7287* |
| ENSMUSG00000114942.2 | *Gm49361* |
| ENSMUSG00000071001.5 | *Hrct1* |
| ENSMUSG00000116885.2 | *4930420G21Rik* |
| ENSMUSG00000020660.7 | *Pomc* |
| ENSMUSG00000045777.15 | *Ifitm10* |
| ENSMUSG00000036826.15 | *Igflr1* |
| ENSMUSG00000084120.2 | *Gm11624* |
| ENSMUSG00000073973.5 | *Or52k2* |
| ENSMUSG00000076615.5 | *Ighg3* |
| ENSMUSG00000070448.15 | *Vmn2r89* |
| ENSMUSG00000085779.2 | *Atcayos* |
| ENSMUSG00000107750.2 | *Gm44013* |
| ENSMUSG00000054206.3 | *Gzmm* |
| ENSMUSG00000099478.2 | *Gm28370* |
| ENSMUSG00000040133.3 | *Gpr176* |
| ENSMUSG00000029452.19 | *Tmem116* |
| ENSMUSG00000068818.6 | *Or8w1* |
| ENSMUSG00000100287.2 | *Gm28068* |
| ENSMUSG00000119767.1 | *n-R5s112* |
| ENSMUSG00000120690.1 | *Gm56654* |
| ENSMUSG00000087443.2 | *Ppp1r18os* |
| ENSMUSG00000113698.2 | *Gm47987* |
| ENSMUSG00000041771.15 | *Slc24a4* |
| ENSMUSG00000062751.6 | *Prss1* |
| ENSMUSG00000053338.10 | *Tarm1* |
| ENSMUSG00000086513.4 | *Gvin-ps1* |
| ENSMUSG00000045655.10 | *Fam216b* |
| ENSMUSG00000101603.3 | *Gm28730* |
| ENSMUSG00000040705.4 | *A930016O22Rik* |
| ENSMUSG00000102760.2 | *Gm37258* |
| ENSMUSG00000059900.15 | *Tmem40* |
| ENSMUSG00000096351.3 | *Samd11* |
| ENSMUSG00000120035.1 | *Gm32934* |
| ENSMUSG00000029005.5 | *Draxin* |
| ENSMUSG00000108798.2 | *Gm9521* |
| ENSMUSG00000023403.15 | *Stk31* |
| ENSMUSG00000025467.9 | *Prap1* |
| ENSMUSG00000114629.2 | *Gm48244* |
| ENSMUSG00000075387.3 | *Or1j13* |
| ENSMUSG00000100147.4 | *1700047M11Rik* |
| ENSMUSG00000104632.2 | *Gm42909* |
| ENSMUSG00000064637.3 | *Snora20* |
| ENSMUSG00000081078.2 | *Gm12568* |
| ENSMUSG00000064442.3 | *Gm26225* |
| ENSMUSG00000041681.3 | *Iapp* |
| ENSMUSG00000118305.2 | *Gm50131* |
| ENSMUSG00000095517.2 | *Gm13249* |
| ENSMUSG00000067599.14 | *Klra7* |
| ENSMUSG00000086904.2 | *Gm13404* |
| ENSMUSG00000098164.3 | *Gm5493* |
| ENSMUSG00000090581.10 | *Vmn2r6* |
| ENSMUSG00000111293.2 | *Gm34006* |
| ENSMUSG00000118908.1 | *Gm22997* |
| ENSMUSG00000058385.9 | *H2bc8* |
| ENSMUSG00000086527.2 | *Gm15856* |
| ENSMUSG00000058447.9 | *Gm26920* |
| ENSMUSG00000085059.2 | *Gm11750* |
| ENSMUSG00000111631.2 | *Gm32017* |
| ENSMUSG00000078763.3 | *Slfn1* |
| ENSMUSG00000113880.2 | *A030005L19Rik* |
| ENSMUSG00000116295.3 | *Gm32885* |
| ENSMUSG00000105021.2 | *Gm8234* |
| ENSMUSG00000109560.2 | *Gm8463* |
| ENSMUSG00000105925.2 | *Gm43846* |
| ENSMUSG00000108059.2 | *Gm44369* |
| ENSMUSG00000102316.2 | *Gm37629* |
| ENSMUSG00000120098.1 | *Gm57432* |
| ENSMUSG00000118972.1 | *Gm22306* |
| ENSMUSG00000081752.4 | *Sms-ps* |
| ENSMUSG00000121398.1 | *Slfn10-ps* |
| ENSMUSG00000024736.16 | *Tmem132a* |
| ENSMUSG00000005800.4 | *Mmp8* |
| ENSMUSG00000087452.3 | *Gm11998* |
| ENSMUSG00000034059.15 | *Ypel4* |
| ENSMUSG00000104851.2 | *E030026E10Rik* |
| ENSMUSG00000024857.17 | *Cabp2* |
| ENSMUSG00000119368.1 | *Gm22170* |
| ENSMUSG00000110240.2 | *Gm45486* |
| ENSMUSG00000097812.2 | *Gm26812* |
| ENSMUSG00000022584.15 | *Ly6c2* |
| ENSMUSG00000066515.4 | *Klk1b3* |
| ENSMUSG00000115539.2 | *Gm6997* |
| ENSMUSG00000087365.10 | *C430049B03Rik* |
| ENSMUSG00000039760.9 | *Il22ra2* |
| ENSMUSG00000100210.3 | *H3c7* |
| ENSMUSG00000065080.3 | *Gm26497* |
| ENSMUSG00000069307.8 | *H2bc23* |
| ENSMUSG00000106334.2 | *Gm43549* |
| ENSMUSG00000117796.2 | *Gm35406* |
| ENSMUSG00000100001.2 | *1810007D17Rik* |
| ENSMUSG00000097622.3 | *A330033J07Rik* |
| ENSMUSG00000119523.1 | *n-R5s113* |
| ENSMUSG00000120811.1 | *Gm56580* |
| ENSMUSG00000043410.17 | *Hfm1* |
| ENSMUSG00000089744.3 | *Gm16146* |
| ENSMUSG00000104988.2 | *Gm43622* |
| ENSMUSG00000118310.2 | *Gm50268* |
| ENSMUSG00000108112.2 | *Gm45193* |
| ENSMUSG00000026204.16 | *Ptprn* |
| ENSMUSG00000021541.15 | *Trpc7* |
| ENSMUSG00000032690.17 | *Oas2* |
| ENSMUSG00000003273.15 | *Car11* |
| ENSMUSG00000025330.7 | *Padi4* |
| ENSMUSG00000121342.1 | *ENSMUSG00000121342* |
| ENSMUSG00000107461.2 | *Gm44045* |
| ENSMUSG00000121499.1 | *Ly6a2* |
| ENSMUSG00000102153.2 | *Gm37474* |
| ENSMUSG00000037661.15 | *Gpr160* |
| ENSMUSG00000119260.1 | *Gm23731* |
| ENSMUSG00000097930.4 | *C330002G04Rik* |
| ENSMUSG00000020676.3 | *Ccl11* |
| ENSMUSG00000099241.3 | *Gvin-ps2* |
| ENSMUSG00000102137.2 | *Rpl28-ps2* |
| ENSMUSG00002076939.1 | *Gm56065* |
| ENSMUSG00000097938.2 | *Gm26933* |
| ENSMUSG00000071052.5 | *Rpl7a-ps5* |
| ENSMUSG00000102046.2 | *2810404M03Rik* |
| ENSMUSG00000120826.1 | *Gm57409* |
| ENSMUSG00000097882.4 | *0610038B21Rik* |
| ENSMUSG00000086638.8 | *4930405A21Rik* |
| ENSMUSG00000050345.10 | *4930486L24Rik* |
| ENSMUSG00000104222.2 | *Gm7292* |
| ENSMUSG00000107976.2 | *Gm44043* |
| ENSMUSG00000120482.1 | *Gm36440* |
| ENSMUSG00000114253.3 | *Gm47798* |
| ENSMUSG00000037346.6 | *Hrh4* |
| ENSMUSG00000097393.10 | *D030068K23Rik* |
| ENSMUSG00000086591.2 | *Dnah2os* |
| ENSMUSG00000102142.2 | *Gm26930* |
| ENSMUSG00000057060.9 | *Slc35f3* |
| ENSMUSG00000079055.11 | *Slc8a3* |
| ENSMUSG00000074604.10 | *Mgst2* |
| ENSMUSG00000107261.2 | *Trmt112-ps1* |
| ENSMUSG00000108766.2 | *Gm44760* |
| ENSMUSG00000020787.15 | *P2rx1* |
| ENSMUSG00000112831.2 | *Gm35533* |
| ENSMUSG00000121340.1 | *1810018F18Rik* |
| ENSMUSG00000064624.3 | *Gm22305* |
| ENSMUSG00000056494.8 | *Cngb3* |
| ENSMUSG00000072573.4 | *Gm10369* |
| ENSMUSG00000021464.16 | *Ror2* |
| ENSMUSG00000073491.12 | *Ifi213* |
| ENSMUSG00000117332.2 | *Gm49942* |
| ENSMUSG00000086907.2 | *Gm15298* |
| ENSMUSG00000082532.2 | *Rpl31-ps6* |
| ENSMUSG00000103749.2 | *Pcdhgb5* |
| ENSMUSG00000119730.1 | *n-R5s106* |
| ENSMUSG00000104396.2 | *Gm37959* |
| ENSMUSG00000073954.6 | *Or52e3* |
| ENSMUSG00000000093.7 | *Tbx2* |
| ENSMUSG00000028457.19 | *Atp8b5* |
| ENSMUSG00000097476.2 | *Gm26583* |
| ENSMUSG00000044220.14 | *Nkx2-3* |
| ENSMUSG00000107729.2 | *B230112I24Rik* |
| ENSMUSG00000120049.1 | *Gm57109* |
| ENSMUSG00000046610.16 | *Oacyl* |
| ENSMUSG00000101724.2 | *Gm29453* |
| ENSMUSG00000119058.1 | *Gm22628* |
| ENSMUSG00000119943.1 | *Gm46781* |
| ENSMUSG00000104947.2 | *Gm43721* |
| ENSMUSG00000114055.2 | *Gm32089* |
| ENSMUSG00000049315.5 | *Or1j19* |
| ENSMUSG00000103382.2 | *Gm37755* |
| ENSMUSG00000062987.7 | *Or2d2b* |
| ENSMUSG00000108076.2 | *9530086O07Rik* |
| ENSMUSG00000064686.3 | *Gm25810* |
| ENSMUSG00000077577.4 | *Gm24890* |
| ENSMUSG00000075167.4 | *Or5t16* |
| ENSMUSG00000030124.3 | *Lag3* |
| ENSMUSG00000067724.6 | *Gbx1* |
| ENSMUSG00000115000.2 | *Gm49192* |
| ENSMUSG00000120876.1 | *Gm56941* |
| ENSMUSG00000034226.8 | *Rhov* |
| ENSMUSG00000092094.6 | *Zfp804b* |
| ENSMUSG00000111064.2 | *Gm30285* |
| ENSMUSG00000053626.6 | *Tll1* |
| ENSMUSG00000023132.9 | *Gzma* |
| ENSMUSG00002074933.1 | *Gm55532* |
| ENSMUSG00000052767.6 | *Gm12703* |
| ENSMUSG00000089827.2 | *1700023H06Rik* |
| ENSMUSG00000062380.5 | *Tubb3* |
| ENSMUSG00000114656.2 | *2810403G07Rik* |
| ENSMUSG00000096753.8 | *Fam181a* |
| ENSMUSG00000121274.1 | *Gm57180* |
| ENSMUSG00000086533.4 | *Mypopos* |
| ENSMUSG00000043263.14 | *Ifi209* |
| ENSMUSG00000110141.2 | *Gm45684* |
| ENSMUSG00000098934.2 | *Gvin-ps4* |
| ENSMUSG00000102813.2 | *Gm37795* |
| ENSMUSG00000108592.3 | *Gm38973* |
| ENSMUSG00000050014.9 | *Apol10b* |
| ENSMUSG00000065107.4 | *n-R5s88* |
| ENSMUSG00000119204.1 | *n-R5s107* |
| ENSMUSG00000096999.2 | *Gm26793* |
| ENSMUSG00000084437.3 | *Gm23428* |
| ENSMUSG00000121130.1 | *Gm57150* |
| ENSMUSG00000049280.5 | *Or10ab5* |
| ENSMUSG00000087263.2 | *Gm15726* |
| ENSMUSG00000059108.5 | *Ifitm6* |
| ENSMUSG00000036185.10 | *Sapcd1* |
| ENSMUSG00000083912.2 | *Gm5391* |
| ENSMUSG00000102689.2 | *Gm37217* |
| ENSMUSG00000030144.5 | *Clec4d* |
| ENSMUSG00000084885.8 | *3010001F23Rik* |
| ENSMUSG00000106185.2 | *Gm7631* |
| ENSMUSG00000071517.7 | *Gm10334* |
| ENSMUSG00000117872.2 | *A530088E08Rik* |
| ENSMUSG00000078954.10 | *Arhgap8* |
| ENSMUSG00000078789.10 | *Dph1* |
| ENSMUSG00000105322.2 | *Gm43751* |
| ENSMUSG00000103373.2 | *Gm37238* |
| ENSMUSG00000095865.2 | *Gm13237* |
| ENSMUSG00000071519.7 | *Prss3* |
| ENSMUSG00000103585.2 | *Pcdhgb4* |
| ENSMUSG00000112139.2 | *Gm47431* |
| ENSMUSG00000069305.4 | *H4c18* |
| ENSMUSG00000120573.1 | *Gm57291* |
| ENSMUSG00000090556.7 | *Or2h2b-ps1* |
| ENSMUSG00000026581.15 | *Sell* |
| ENSMUSG00000085889.2 | *1700054M17Rik* |
| ENSMUSG00000038357.11 | *Camp* |
| ENSMUSG00000085918.2 | *Gm13032* |
| ENSMUSG00000074482.6 | *Gm16589* |
| ENSMUSG00000112324.2 | *Gm47939* |
| ENSMUSG00000030427.18 | *Lilra6* |
| ENSMUSG00000030717.10 | *Nupr1* |
| ENSMUSG00000073144.6 | *4930599N23Rik* |
| ENSMUSG00000048996.7 | *Or1f12* |
| ENSMUSG00000017300.10 | *Tnnc2* |
| ENSMUSG00000032936.14 | *Camkv* |
| ENSMUSG00000073433.12 | *Arhgdig* |
| ENSMUSG00000039209.13 | *Rpl39l* |
| ENSMUSG00000047897.11 | *Ripply2* |
| ENSMUSG00000063698.10 | *Sfxn4* |
| ENSMUSG00000083606.4 | *Gm15916* |

**Table S8: The 300 most highly downregulated genes in Kupffer cells after 16 hours of treatment with LNP.**

| ENSMUSG00000028599.11 | *Tnfrsf1b* |
| --- | --- |
| ENSMUSG00000004651.7 | *Tyr* |
| ENSMUSG00000059657.6 | *Stfa2l1* |
| ENSMUSG00000104736.2 | *Gm33609* |
| ENSMUSG00000032845.17 | *Alpk2* |
| ENSMUSG00000058818.14 | *Pirb* |
| ENSMUSG00000030214.8 | *Plbd1* |
| ENSMUSG00000053411.17 | *Cbx7* |
| ENSMUSG00000032656.15 | *Marchf3* |
| ENSMUSG00000090174.2 | *Gm10612* |
| ENSMUSG00000034463.5 | *Scara3* |
| ENSMUSG00000045312.13 | *Lhfpl2* |
| ENSMUSG00000110755.3 | *BC049987* |
| ENSMUSG00000021186.10 | *Fbln5* |
| ENSMUSG00000082165.2 | *Gm7091* |
| ENSMUSG00000038668.15 | *Lpar1* |
| ENSMUSG00000009378.5 | *Slc16a12* |
| ENSMUSG00000059319.4 | *Or14c41* |
| ENSMUSG00000030560.18 | *Ctsc* |
| ENSMUSG00000078653.5 | *Cntd1* |
| ENSMUSG00000106472.2 | *Gm43111* |
| ENSMUSG00000037922.14 | *Bank1* |
| ENSMUSG00000114529.2 | *Gm4939* |
| ENSMUSG00000020256.15 | *Aldh1l2* |
| ENSMUSG00000039814.16 | *Xkr5* |
| ENSMUSG00000105495.2 | *Gm42995* |
| ENSMUSG00000000244.18 | *Tspan32* |
| ENSMUSG00000030762.12 | *Aqp8* |
| ENSMUSG00000032274.10 | *Cyp19a1* |
| ENSMUSG00000049321.18 | *Zfp2* |
| ENSMUSG00000084221.2 | *Hmgb1-ps4* |
| ENSMUSG00000051969.8 | *Tlr11* |
| ENSMUSG00000111531.2 | *Gm32688* |
| ENSMUSG00000018570.18 | *2810408A11Rik* |
| ENSMUSG00000001494.7 | *Sost* |
| ENSMUSG00000044017.17 | *Adgrd1* |
| ENSMUSG00000028678.14 | *Kif2c* |
| ENSMUSG00000017466.11 | *Timp2* |
| ENSMUSG00000036278.8 | *Macrod1* |
| ENSMUSG00000059089.5 | *Fcgr4* |
| ENSMUSG00000083929.5 | *Gm10600* |
| ENSMUSG00000073678.5 | *Pgap1* |
| ENSMUSG00000112400.3 | *Gm47725* |
| ENSMUSG00000113626.3 | *Gm7240* |
| ENSMUSG00000029126.8 | *Nsg1* |
| ENSMUSG00000031877.10 | *Ces2g* |
| ENSMUSG00000051177.17 | *Plcb1* |
| ENSMUSG00000006411.13 | *Nectin4* |
| ENSMUSG00000108819.2 | *Gm45177* |
| ENSMUSG00000030069.16 | *Prok2* |
| ENSMUSG00000078673.11 | *Mup19* |
| ENSMUSG00000073492.3 | *Gm10521* |
| ENSMUSG00000103502.2 | *9330121J05Rik* |
| ENSMUSG00000020914.18 | *Top2a* |
| ENSMUSG00000068877.13 | *Selenbp2* |
| ENSMUSG00000028182.15 | *Lrriq3* |
| ENSMUSG00000107689.3 | *Gm44386* |
| ENSMUSG00000022534.15 | *Mefv* |
| ENSMUSG00000027219.14 | *Slc28a2* |
| ENSMUSG00000121134.1 | *Gm57446* |
| ENSMUSG00000026683.15 | *Nuf2* |
| ENSMUSG00000067818.7 | *Myl9* |
| ENSMUSG00000057280.16 | *Musk* |
| ENSMUSG00000113070.2 | *Gm48420* |
| ENSMUSG00000044229.10 | *Nxpe4* |
| ENSMUSG00000027015.5 | *Cybrd1* |
| ENSMUSG00000031442.22 | *Mcf2l* |
| ENSMUSG00000036777.9 | *Anln* |
| ENSMUSG00000004105.9 | *Angptl2* |
| ENSMUSG00000027398.14 | *Il1b* |
| ENSMUSG00000026463.18 | *Atp2b4* |
| ENSMUSG00000105186.2 | *Gm43778* |
| ENSMUSG00000041762.17 | *Gpr155* |
| ENSMUSG00000056596.9 | *Trnp1* |
| ENSMUSG00000107512.2 | *Gm44433* |
| ENSMUSG00000058656.14 | *Samd12* |
| ENSMUSG00000004473.11 | *Clec11a* |
| ENSMUSG00000030246.13 | *Ldhb* |
| ENSMUSG00000111939.2 | *Gm48177* |
| ENSMUSG00000089942.10 | *Pira2* |
| ENSMUSG00000081665.3 | *Pira1* |
| ENSMUSG00000099687.4 | *Or51b6b* |
| ENSMUSG00000111609.2 | *Gm6713* |
| ENSMUSG00000044201.11 | *Cdc25c* |
| ENSMUSG00000023349.15 | *Clec4n* |
| ENSMUSG00000044365.16 | *Cxxc4* |
| ENSMUSG00000022033.10 | *Pbk* |
| ENSMUSG00000066684.11 | *Pilrb1* |
| ENSMUSG00000045275.18 | *Lca5l* |
| ENSMUSG00000100838.2 | *Gm29094* |
| ENSMUSG00000042988.11 | *Notum* |
| ENSMUSG00000048779.6 | *P2ry6* |
| ENSMUSG00000042453.15 | *Reln* |
| ENSMUSG00000021087.19 | *Rtn1* |
| ENSMUSG00000032076.21 | *Cadm1* |
| ENSMUSG00000048065.9 | *Cyb5r2* |
| ENSMUSG00000032528.6 | *Vipr1* |
| ENSMUSG00000104912.2 | *Gm43023* |
| ENSMUSG00000030004.7 | *Nat8* |
| ENSMUSG00000034687.9 | *Fras1* |
| ENSMUSG00000081113.2 | *Gm7308* |
| ENSMUSG00000120878.1 | *Gm56663* |
| ENSMUSG00000038816.15 | *Ctnnal1* |
| ENSMUSG00000028558.15 | *Calr4* |
| ENSMUSG00000106450.2 | *Gm43018* |
| ENSMUSG00000041577.6 | *Prelp* |
| ENSMUSG00000086969.2 | *4930443O20Rik* |
| ENSMUSG00000045875.14 | *Adra1a* |
| ENSMUSG00000119216.1 | *n-R5s151* |
| ENSMUSG00000028132.16 | *Tlcd4* |
| ENSMUSG00000000983.14 | *Wfdc18* |
| ENSMUSG00000068086.7 | *Cyp2d9* |
| ENSMUSG00000098132.2 | *Rassf10* |
| ENSMUSG00000016918.16 | *Sulf1* |
| ENSMUSG00000106045.2 | *Gm42996* |
| ENSMUSG00000022034.11 | *Esco2* |
| ENSMUSG00000029177.10 | *Cenpa* |
| ENSMUSG00000001741.13 | *Il16* |
| ENSMUSG00000021485.15 | *Mxd3* |
| ENSMUSG00000003948.18 | *Mmd* |
| ENSMUSG00000017309.12 | *Cd300lg* |
| ENSMUSG00000034071.16 | *Zfp551* |
| ENSMUSG00000106186.2 | *Gm43627* |
| ENSMUSG00000042985.8 | *Upk3b* |
| ENSMUSG00000038943.17 | *Prc1* |
| ENSMUSG00000041789.8 | *2700046A07Rik* |
| ENSMUSG00000031495.9 | *Cd209d* |
| ENSMUSG00000045404.17 | *Kcnk13* |
| ENSMUSG00000049119.15 | *Fam110b* |
| ENSMUSG00000006576.17 | *Slc4a3* |
| ENSMUSG00000054383.3 | *Pnma1* |
| ENSMUSG00000031342.18 | *Gpm6b* |
| ENSMUSG00000013089.16 | *Etv5* |
| ENSMUSG00000071347.4 | *C1qtnf9* |
| ENSMUSG00000020808.4 | *Pimreg* |
| ENSMUSG00000066682.12 | *Pilrb2* |
| ENSMUSG00000086448.2 | *9330162012Rik* |
| ENSMUSG00000085603.4 | *Gm11346* |
| ENSMUSG00000019232.15 | *Etnppl* |
| ENSMUSG00000113622.2 | *Gm49749* |
| ENSMUSG00000027864.10 | *Ptgfrn* |
| ENSMUSG00000026177.12 | *Slc11a1* |
| ENSMUSG00000078683.10 | *Mup1* |
| ENSMUSG00000004347.18 | *Pde1c* |
| ENSMUSG00000110439.2 | *Mup22* |
| ENSMUSG00000030865.5 | *Chp2* |
| ENSMUSG00000053897.16 | *Slc39a8* |
| ENSMUSG00000040488.19 | *Ltbp4* |
| ENSMUSG00000056220.15 | *Pla2g4a* |
| ENSMUSG00000034641.3 | *Cd300ld* |
| ENSMUSG00000078719.3 | *Msmp* |
| ENSMUSG00000041559.8 | *Fmod* |
| ENSMUSG00000102620.2 | *Gm37675* |
| ENSMUSG00000026622.16 | *Nek2* |
| ENSMUSG00000120007.1 | *Gm17619* |
| ENSMUSG00000114243.2 | *Gm48163* |
| ENSMUSG00000053063.12 | *Clec12a* |
| ENSMUSG00000120309.1 | *Gm56745* |
| ENSMUSG00000032254.11 | *Kif23* |
| ENSMUSG00000040264.11 | *Gbp2b* |
| ENSMUSG00000051906.16 | *Cd209f* |
| ENSMUSG00000049037.9 | *Clec4a1* |
| ENSMUSG00000113571.2 | *Gm48137* |
| ENSMUSG00000026587.16 | *Astn1* |
| ENSMUSG00000078674.3 | *Mup18* |
| ENSMUSG00000070509.16 | *Rgma* |
| ENSMUSG00000056752.17 | *Dnah9* |
| ENSMUSG00000024109.19 | *Nrxn1* |
| ENSMUSG00000021684.18 | *Pde8b* |
| ENSMUSG00000099027.2 | *Gm27176* |
| ENSMUSG00000057191.15 | *AB124611* |
| ENSMUSG00000078687.10 | *Mup8* |
| ENSMUSG00000106121.2 | *Gm42679* |
| ENSMUSG00000023336.7 | *Wfdc1* |
| ENSMUSG00000036298.11 | *Slc2a13* |
| ENSMUSG00000067158.10 | *Col4a4* |
| ENSMUSG00000051439.8 | *Cd14* |
| ENSMUSG00000036782.14 | *Klhl13* |
| ENSMUSG00000030187.16 | *Klra2* |
| ENSMUSG00000116627.2 | *Gm30124* |
| ENSMUSG00000016529.6 | *Il10* |
| ENSMUSG00000083282.4 | *Ctsf* |
| ENSMUSG00000037962.8 | *Rflna* |
| ENSMUSG00000105681.2 | *Gm43428* |
| ENSMUSG00000046192.5 | *Iqub* |
| ENSMUSG00000073830.12 | *Mup14* |
| ENSMUSG00000033192.6 | *Lpcat2* |
| ENSMUSG00000079580.10 | *Tmem217* |
| ENSMUSG00000020388.13 | *Pdlim4* |
| ENSMUSG00000112189.2 | *C730027H18Rik* |
| ENSMUSG00000004730.16 | *Adgre1* |
| ENSMUSG00000113701.3 | *B230303A05Rik* |
| ENSMUSG00000034648.10 | *Lrrn1* |
| ENSMUSG00000073879.5 | *Gm54215* |
| ENSMUSG00000045502.7 | *Hcar2* |
| ENSMUSG00000105720.2 | *Gm42440* |
| ENSMUSG00000104950.2 | *4833413G10Rik* |
| ENSMUSG00000072849.11 | *Serpina1e* |
| ENSMUSG00000094707.4 | *A830019P07Rik* |
| ENSMUSG00000056870.10 | *Gulp1* |
| ENSMUSG00000102037.2 | *Bcl2a1a* |
| ENSMUSG00000031698.15 | *Mylk3* |
| ENSMUSG00000089929.2 | *Bcl2a1b* |
| ENSMUSG00000035004.4 | *Igsf6* |
| ENSMUSG00000028003.7 | *Lrat* |
| ENSMUSG00000028175.16 | *Depdc1a* |
| ENSMUSG00000067786.17 | *Nnat* |
| ENSMUSG00000038591.4 | *Colec10* |
| ENSMUSG00000011008.14 | *Mcoln2* |
| ENSMUSG00000032915.7 | *Adgre4* |
| ENSMUSG00000049404.8 | *Rarres1* |
| ENSMUSG00000075012.5 | *Fjx1* |
| ENSMUSG00000051517.15 | *Arhgef39* |
| ENSMUSG00000032649.15 | *Colgalt2* |
| ENSMUSG00000017417.15 | *Plxdc1* |
| ENSMUSG00000038576.16 | *Susd4* |
| ENSMUSG00000045555.4 | *Mettl24* |
| ENSMUSG00000096688.2 | *Mup17* |
| ENSMUSG00000024730.8 | *Ms4a8a* |
| ENSMUSG00000054256.12 | *Msi1* |
| ENSMUSG00000043300.3 | *B3galnt1* |
| ENSMUSG00000051359.16 | *Ncald* |
| ENSMUSG00000051257.4 | *Trap1a* |
| ENSMUSG00000099974.2 | *Bcl2a1d* |
| ENSMUSG00000058523.3 | *Mup5* |
| ENSMUSG00000015354.9 | *Pcolce2* |
| ENSMUSG00000020429.8 | *Igfbp1* |
| ENSMUSG00000022901.14 | *Cd86* |
| ENSMUSG00000069515.7 | *Lyz1* |
| ENSMUSG00000038777.20 | *Sema6c* |
| ENSMUSG00000015568.17 | *Lpl* |
| ENSMUSG00000107655.2 | *Gm44220* |
| ENSMUSG00000078780.8 | *Gm5150* |
| ENSMUSG00000035283.6 | *Adrb1* |
| ENSMUSG00000048498.9 | *Cd300e* |
| ENSMUSG00000027559.6 | *Car3* |
| ENSMUSG00000056673.15 | *Kdm5d* |
| ENSMUSG00000078686.12 | *Mup9* |
| ENSMUSG00000035836.3 | *Ugt2b1* |
| ENSMUSG00000020811.17 | *Wscd1* |
| ENSMUSG00000024044.20 | *Epb41l3* |
| ENSMUSG00000078689.9 | *Mup6* |
| ENSMUSG00000030217.3 | *Art4* |
| ENSMUSG00000033361.14 | *Prrg3* |
| ENSMUSG00000085042.10 | *Abhd11os* |
| ENSMUSG00000026981.16 | *Il1rn* |
| ENSMUSG00000121091.1 | *Gm56861* |
| ENSMUSG00000004359.17 | *Spic* |
| ENSMUSG00000069792.6 | *Wfdc17* |
| ENSMUSG00000030255.14 | *Sspn* |
| ENSMUSG00000033578.8 | *Tmem35a* |
| ENSMUSG00000039977.17 | *Deup1* |
| ENSMUSG00000069516.9 | *Lyz2* |
| ENSMUSG00000038357.11 | *Camp* |
| ENSMUSG00000005124.11 | *Ccn4* |
| ENSMUSG00000024621.17 | *Csf1r* |
| ENSMUSG00000108358.2 | *Gm44509* |
| ENSMUSG00000045551.8 | *Fpr1* |
| ENSMUSG00000028444.18 | *Cntfr* |
| ENSMUSG00000001128.8 | *Cfp* |
| ENSMUSG00000094793.2 | *Mup12* |
| ENSMUSG00000026768.11 | *Itga8* |
| ENSMUSG00000085095.3 | *Gm15635* |
| ENSMUSG00000009292.19 | *Trpm2* |
| ENSMUSG00000068457.15 | *Uty* |
| ENSMUSG00000050447.16 | *Lypd6* |
| ENSMUSG00000090338.3 | *Gm17081* |
| ENSMUSG00000078597.3 | *Cyp4a12b* |
| ENSMUSG00000051682.16 | *Treml4* |
| ENSMUSG00000036362.3 | *P2ry13* |
| ENSMUSG00000002489.17 | *Tiam1* |
| ENSMUSG00000079597.3 | *Cstdc4* |
| ENSMUSG00000041333.7 | *Mup4* |
| ENSMUSG00000004814.11 | *Ccl24* |
| ENSMUSG00000034783.8 | *Cd207* |
| ENSMUSG00000042115.5 | *Klhdc8a* |
| ENSMUSG00000073842.11 | *Mup7* |
| ENSMUSG00000022181.17 | *C6* |
| ENSMUSG00000066153.3 | *Mup21* |
| ENSMUSG00000079355.6 | *Ackr4* |
| ENSMUSG00000060402.9 | *Chst8* |
| ENSMUSG00000070873.6 | *Lilra5* |
| ENSMUSG00000086843.4 | *E030013I19Rik* |
| ENSMUSG00000096674.2 | *Mup15* |
| ENSMUSG00000120881.1 | *Gm56664* |
| ENSMUSG00000073834.11 | *Mup11* |
| ENSMUSG00000055546.7 | *Timd4* |
| ENSMUSG00000038754.6 | *Elovl3* |
| ENSMUSG00000026390.8 | *Marco* |
| ENSMUSG00000078672.3 | *Mup20* |
| ENSMUSG00000069049.12 | *Eif2s3y* |
| ENSMUSG00000008845.10 | *Cd163* |
| ENSMUSG00000066071.7 | *Cyp4a12a* |
| ENSMUSG00000015854.8 | *Cd5l* |
| ENSMUSG00000044206.4 | *Vsig4* |
| ENSMUSG00000019874.12 | *Fabp7* |
| ENSMUSG00000082644.2 | *Mup-ps19* |
| ENSMUSG00000069045.12 | *Ddx3y* |
| ENSMUSG00000032725.11 | *Folr2* |
| ENSMUSG00000014542.4 | *Clec4f* |

**Table S9: The 300 most highly downregulated genes in hepatocytes after 16 hours of treatment with LNP.**

| ENSMUSG00000114097.2 | *Gm35615* |
| --- | --- |
| ENSMUSG00000101875.2 | *Gm6028* |
| ENSMUSG00000030664.4 | *Sox6os* |
| ENSMUSG00000080021.6 | *Gm5915* |
| ENSMUSG00000042248.5 | *Cyp2c37* |
| ENSMUSG00000030804.9 | *Gm21974* |
| ENSMUSG00000025429.10 | *Pstpip2* |
| ENSMUSG00000054493.3 | *Gm9947* |
| ENSMUSG00000037071.4 | *Scd1* |
| ENSMUSG00000097691.2 | *9030616G12Rik* |
| ENSMUSG00000110381.2 | *Gm34370* |
| ENSMUSG00000032418.16 | *Me1* |
| ENSMUSG00000089929.2 | *Bcl2a1b* |
| ENSMUSG00000102753.2 | *Gm37056* |
| ENSMUSG00000095929.3 | *Or5p63* |
| ENSMUSG00000100866.2 | *Gm28830* |
| ENSMUSG00000030364.7 | *Clec2h* |
| ENSMUSG00000110063.2 | *Gm31045* |
| ENSMUSG00000078799.6 | *Sult2a5* |
| ENSMUSG00000078650.3 | *G6pc* |
| ENSMUSG00000021884.19 | *Hacl1* |
| ENSMUSG00000024044.20 | *Epb41l3* |
| ENSMUSG00000021423.7 | *Ly86* |
| ENSMUSG00000093726.2 | *Gm20667* |
| ENSMUSG00000120635.1 | *Gm56857* |
| ENSMUSG00000022157.9 | *Mcpt8* |
| ENSMUSG00000082110.2 | *Gm11196* |
| ENSMUSG00000086279.3 | *Gm15634* |
| ENSMUSG00000025175.13 | *Fn3k* |
| ENSMUSG00000066896.7 | *Or7e178* |
| ENSMUSG00000112926.2 | *Gm7172* |
| ENSMUSG00000086614.3 | *Gm14330* |
| ENSMUSG00000023070.7 | *Rgn* |
| ENSMUSG00000038217.14 | *Tlcd2* |
| ENSMUSG00000028989.4 | *Angptl7* |
| ENSMUSG00000001014.7 | *Icam4* |
| ENSMUSG00000053303.15 | *Slc22a26* |
| ENSMUSG00000111074.2 | *Gm36435* |
| ENSMUSG00000062365.5 | *Gm4968* |
| ENSMUSG00000027403.13 | *Tgm6* |
| ENSMUSG00000067225.3 | *Cyp2c54* |
| ENSMUSG00000115242.2 | *Gm49018* |
| ENSMUSG00000075125.3 | *Or4p20* |
| ENSMUSG00000020897.13 | *Aurkb* |
| ENSMUSG00000035472.15 | *Slc25a21* |
| ENSMUSG00000070550.3 | *Mrgprb4* |
| ENSMUSG00000059375.6 | *Vmn1r33* |
| ENSMUSG00000026683.15 | *Nuf2* |
| ENSMUSG00000036223.17 | *Ska1* |
| ENSMUSG00000079263.9 | *Gm6614* |
| ENSMUSG00000045055.6 | *Rpsa-ps2* |
| ENSMUSG00000087400.10 | *Gm15270* |
| ENSMUSG00000006398.16 | *Cdc20* |
| ENSMUSG00000103037.2 | *Pcdhgb1* |
| ENSMUSG00000021228.16 | *Acot3* |
| ENSMUSG00000028194.16 | *Ddah1* |
| ENSMUSG00000051682.16 | *Treml4* |
| ENSMUSG00000038641.13 | *Akr1d1* |
| ENSMUSG00000022347.9 | *A1bg* |
| ENSMUSG00000074547.5 | *Rps8-ps4* |
| ENSMUSG00000054827.13 | *Cyp2c50* |
| ENSMUSG00000020150.14 | *Gamt* |
| ENSMUSG00000038656.6 | *Cyp3a16* |
| ENSMUSG00000027533.11 | *Fabp5* |
| ENSMUSG00000024397.15 | *Aif1* |
| ENSMUSG00000118117.3 | *Gm30961* |
| ENSMUSG00000062224.7 | *4933411G06Rik* |
| ENSMUSG00000106917.2 | *Gm7832* |
| ENSMUSG00000109171.2 | *Gm5735* |
| ENSMUSG00000097047.3 | *1110020A21Rik* |
| ENSMUSG00000085952.2 | *Gm12315* |
| ENSMUSG00000098207.2 | *Arl14* |
| ENSMUSG00000030562.18 | *Nox4* |
| ENSMUSG00000082579.2 | *Gm11979* |
| ENSMUSG00000027579.7 | *Srms* |
| ENSMUSG00000087579.9 | *Hectd2os* |
| ENSMUSG00000029482.5 | *Aacs* |
| ENSMUSG00000017309.12 | *Cd300lg* |
| ENSMUSG00000105135.2 | *Gm43667* |
| ENSMUSG00000049685.9 | *Cyp2g1* |
| ENSMUSG00000106736.2 | *Gm42573* |
| ENSMUSG00000055254.16 | *Ntrk2* |
| ENSMUSG00000042097.18 | *Zfp239* |
| ENSMUSG00000030474.10 | *Siglece* |
| ENSMUSG00000053613.7 | *Notumos* |
| ENSMUSG00000050069.4 | *Grem2* |
| ENSMUSG00000060198.8 | *Gm11353* |
| ENSMUSG00000089694.3 | *Nat8f7* |
| ENSMUSG00000028240.3 | *Cyp7a1* |
| ENSMUSG00000118533.2 | *Gm53049* |
| ENSMUSG00000117405.2 | *Rpl19-ps7* |
| ENSMUSG00000026177.12 | *Slc11a1* |
| ENSMUSG00000114895.2 | *4930579J19Rik* |
| ENSMUSG00000074375.4 | *Sult2a3* |
| ENSMUSG00000109412.2 | *Gm44535* |
| ENSMUSG00000034641.3 | *Cd300ld* |
| ENSMUSG00000092008.3 | *Cyp2c69* |
| ENSMUSG00000041431.17 | *Ccnb1* |
| ENSMUSG00000114886.2 | *Gm48432* |
| ENSMUSG00000069516.9 | *Lyz2* |
| ENSMUSG00000098915.2 | *Rpl15-ps2* |
| ENSMUSG00000080896.6 | *Gm14567* |
| ENSMUSG00000082715.2 | *Gm11633* |
| ENSMUSG00000120050.1 | *Gm52113* |
| ENSMUSG00000108431.2 | *Gm42375* |
| ENSMUSG00000097706.2 | *E030037K01Rik* |
| ENSMUSG00000020330.17 | *Hmmr* |
| ENSMUSG00000078798.5 | *Sult2a1* |
| ENSMUSG00000075524.4 | *4930407I10Rik* |
| ENSMUSG00000025153.10 | *Fasn* |
| ENSMUSG00000120149.1 | *Gm36180* |
| ENSMUSG00000107168.2 | *Gm42507* |
| ENSMUSG00000115347.2 | *Gm8238* |
| ENSMUSG00000115003.2 | *Gm48933* |
| ENSMUSG00000066682.12 | *Pilrb2* |
| ENSMUSG00000085546.2 | *Gm14252* |
| ENSMUSG00000024292.16 | *Cyp4f14* |
| ENSMUSG00000069456.5 | *Rdh16* |
| ENSMUSG00000044461.7 | *Shisa2* |
| ENSMUSG00000062896.4 | *Rpl31-ps11* |
| ENSMUSG00000038180.12 | *Spag4* |
| ENSMUSG00000021747.13 | *Cfap20dc* |
| ENSMUSG00000026938.11 | *Fcna* |
| ENSMUSG00000071419.8 | *Rps15-ps2* |
| ENSMUSG00000065952.14 | *Rps23rg1* |
| ENSMUSG00000078917.4 | *Gm11281* |
| ENSMUSG00000103505.2 | *Gm38374* |
| ENSMUSG00000038092.7 | *Hsd3b5* |
| ENSMUSG00000121424.1 | *ENSMUSG00000121424* |
| ENSMUSG00000004038.10 | *Gstm3* |
| ENSMUSG00000104415.2 | *Gm37069* |
| ENSMUSG00000058802.6 | *Or1o2* |
| ENSMUSG00000095427.3 | *Rps2-ps6* |
| ENSMUSG00000066270.4 | *Gm10157* |
| ENSMUSG00000104868.2 | *Gm9954* |
| ENSMUSG00000037406.8 | *Htra4* |
| ENSMUSG00000120615.1 | *Gm56802* |
| ENSMUSG00000043020.14 | *Dnai3* |
| ENSMUSG00000010651.5 | *Acaa1b* |
| ENSMUSG00000107606.2 | *Gm43873* |
| ENSMUSG00000097231.3 | *Gm26852* |
| ENSMUSG00000071343.3 | *Gm10327* |
| ENSMUSG00000044811.15 | *Cd300c2* |
| ENSMUSG00000042834.16 | *Nrep* |
| ENSMUSG00000115023.2 | *Gm48967* |
| ENSMUSG00000083907.3 | *Plk-ps1* |
| ENSMUSG00000031147.9 | *Magix* |
| ENSMUSG00000095419.2 | *Gm14328* |
| ENSMUSG00000092056.3 | *Gm7289* |
| ENSMUSG00000030519.15 | *Apba2* |
| ENSMUSG00000115022.2 | *Gm49387* |
| ENSMUSG00000119971.1 | *Gm56821* |
| ENSMUSG00000090894.5 | *Or5v1* |
| ENSMUSG00000042988.11 | *Notum* |
| ENSMUSG00000037482.3 | *Erv3* |
| ENSMUSG00000019232.15 | *Etnppl* |
| ENSMUSG00000033595.8 | *Lgi3* |
| ENSMUSG00000102659.2 | *Gm37077* |
| ENSMUSG00000022901.14 | *Cd86* |
| ENSMUSG00000083722.3 | *Adgrd2-ps* |
| ENSMUSG00000109328.3 | *Or5a21* |
| ENSMUSG00000091019.2 | *Gm7502* |
| ENSMUSG00000003477.6 | *Inmt* |
| ENSMUSG00000020889.12 | *Nr1d1* |
| ENSMUSG00000120316.1 | *Gm57272* |
| ENSMUSG00000001128.8 | *Cfp* |
| ENSMUSG00000094222.2 | *Mup-ps6* |
| ENSMUSG00000027820.13 | *Mme* |
| ENSMUSG00000112397.3 | *Gm10824* |
| ENSMUSG00000000318.17 | *Clec10a* |
| ENSMUSG00000055523.8 | *Gucy2g* |
| ENSMUSG00000120793.1 | *Gm39167* |
| ENSMUSG00000002489.17 | *Tiam1* |
| ENSMUSG00000027762.7 | *Sucnr1* |
| ENSMUSG00000054417.6 | *Cyp3a44* |
| ENSMUSG00000071656.8 | *Lrrn4cl* |
| ENSMUSG00000027690.14 | *Slc2a2* |
| ENSMUSG00000081034.2 | *Gm5944* |
| ENSMUSG00000025002.6 | *Cyp2c55* |
| ENSMUSG00000041828.16 | *Abca8a* |
| ENSMUSG00000055022.15 | *Cntn1* |
| ENSMUSG00000022033.10 | *Pbk* |
| ENSMUSG00000113113.2 | *Gm2614* |
| ENSMUSG00000057933.11 | *Gsta2* |
| ENSMUSG00000028654.14 | *Mycl* |
| ENSMUSG00000070811.7 | *Sult2a2* |
| ENSMUSG00000114019.2 | *Gm47155* |
| ENSMUSG00000026220.7 | *Slc16a14* |
| ENSMUSG00000041220.11 | *Elovl6* |
| ENSMUSG00000031725.9 | *Ces1f* |
| ENSMUSG00000041809.6 | *Efhc1* |
| ENSMUSG00000020649.12 | *Rrm2* |
| ENSMUSG00000051906.16 | *Cd209f* |
| ENSMUSG00000053081.4 | *1700069B07Rik* |
| ENSMUSG00000067719.6 | *Gm10221* |
| ENSMUSG00000026768.11 | *Itga8* |
| ENSMUSG00000032013.7 | *Trim29* |
| ENSMUSG00000111535.3 | *Gm35154* |
| ENSMUSG00000098154.3 | *Gm5787* |
| ENSMUSG00000027408.8 | *Cpxm1* |
| ENSMUSG00000030762.12 | *Aqp8* |
| ENSMUSG00000025519.6 | *Tktl2* |
| ENSMUSG00000028238.7 | *Atp6v0d2* |
| ENSMUSG00000090236.4 | *Car15* |
| ENSMUSG00000031326.3 | *Cdx4* |
| ENSMUSG00000030378.16 | *Sult2a8* |
| ENSMUSG00000105837.6 | *Gm35986* |
| ENSMUSG00000090097.9 | *Or4p21* |
| ENSMUSG00000039873.5 | *Neurl2* |
| ENSMUSG00000038768.9 | *9130409I23Rik* |
| ENSMUSG00000094446.2 | *Gm6344* |
| ENSMUSG00000083947.6 | *Or10al6* |
| ENSMUSG00000117358.3 | *Gm31645* |
| ENSMUSG00000015396.5 | *Cd83* |
| ENSMUSG00000095093.3 | *Vmn2r111* |
| ENSMUSG00000026475.8 | *Rgs16* |
| ENSMUSG00000031494.8 | *Cd209a* |
| ENSMUSG00000004296.15 | *Il12b* |
| ENSMUSG00000115970.3 | *8430426J06Rik* |
| ENSMUSG00000053957.7 | *Gm12474* |
| ENSMUSG00000082436.2 | *Gm11688* |
| ENSMUSG00000111709.2 | *Gm3776* |
| ENSMUSG00000121091.1 | *Gm56861* |
| ENSMUSG00000059852.8 | *Kcng2* |
| ENSMUSG00000020037.16 | *Rfx4* |
| ENSMUSG00000040264.11 | *Gbp2b* |
| ENSMUSG00000104000.2 | *Gm38335* |
| ENSMUSG00000024694.10 | *Keg1* |
| ENSMUSG00000027605.19 | *Acss2* |
| ENSMUSG00000099536.2 | *Gm9569* |
| ENSMUSG00000106786.2 | *Gm42781* |
| ENSMUSG00000108822.2 | *Gm44787* |
| ENSMUSG00000081321.2 | *Gm15304* |
| ENSMUSG00000120881.1 | *Gm56664* |
| ENSMUSG00000079355.6 | *Ackr4* |
| ENSMUSG00000027556.16 | *Car1* |
| ENSMUSG00000059030.6 | *Or14j7* |
| ENSMUSG00000021416.12 | *Eci3* |
| ENSMUSG00000099655.3 | *2310034G01Rik* |
| ENSMUSG00000029663.11 | *Gngt1* |
| ENSMUSG00000106069.3 | *Gm6135* |
| ENSMUSG00000055782.10 | *Abcd2* |
| ENSMUSG00000102611.2 | *Gm37847* |
| ENSMUSG00000106623.2 | *Gm43335* |
| ENSMUSG00000120336.1 | *Gm57195* |
| ENSMUSG00000090610.4 | *Gm3571* |
| ENSMUSG00002075924.1 | *Gm54850* |
| ENSMUSG00000026691.11 | *Fmo3* |
| ENSMUSG00000035686.9 | *Thrsp* |
| ENSMUSG00000061684.7 | *Rpl21-ps8* |
| ENSMUSG00000056978.9 | *Hamp2* |
| ENSMUSG00000102037.2 | *Bcl2a1a* |
| ENSMUSG00000082809.4 | *Gm14150* |
| ENSMUSG00000070873.6 | *Lilra5* |
| ENSMUSG00000038526.15 | *Car14* |
| ENSMUSG00000115604.2 | *Gm7691* |
| ENSMUSG00000074377.13 | *Sult2a4* |
| ENSMUSG00000081207.5 | *Gm13775* |
| ENSMUSG00000027559.6 | *Car3* |
| ENSMUSG00000072596.5 | *Ear2* |
| ENSMUSG00000008845.10 | *Cd163* |
| ENSMUSG00000073835.6 | *Mup-ps12* |
| ENSMUSG00000060044.9 | *Tmem26* |
| ENSMUSG00000081870.2 | *Gm15141* |
| ENSMUSG00000038754.6 | *Elovl3* |
| ENSMUSG00000030147.13 | *Clec4b1* |
| ENSMUSG00000027577.15 | *Chrna4* |
| ENSMUSG00000078452.11 | *Raet1d* |
| ENSMUSG00000048498.9 | *Cd300e* |
| ENSMUSG00000024621.17 | *Csf1r* |
| ENSMUSG00000035948.14 | *Acss3* |
| ENSMUSG00000082173.2 | *Mup-ps10* |
| ENSMUSG00000120776.1 | *Gm34235* |
| ENSMUSG00000032915.7 | *Adgre4* |
| ENSMUSG00000016529.6 | *Il10* |
| ENSMUSG00000089942.10 | *Pira2* |
| ENSMUSG00000018868.5 | *Pnpla5* |
| ENSMUSG00000027870.9 | *Hao2* |
| ENSMUSG00000086201.2 | *Gm6270* |
| ENSMUSG00000036896.6 | *C1qc* |
| ENSMUSG00000077391.3 | *Gm24336* |
| ENSMUSG00000074213.4 | *Gm10642* |
| ENSMUSG00000036887.6 | *C1qa* |
| ENSMUSG00000120853.1 | *Gm57216* |
| ENSMUSG00000032725.11 | *Folr2* |
| ENSMUSG00000116903.2 | *Gm19522* |
| ENSMUSG00000034783.8 | *Cd207* |
| ENSMUSG00000046687.7 | *Gm5424* |
| ENSMUSG00000082065.2 | *Mup-ps14* |
| ENSMUSG00000121161.1 | *Gm31249* |
| ENSMUSG00000055546.7 | *Timd4* |
| ENSMUSG00000022057.9 | *Adamdec1* |
| ENSMUSG00000019874.12 | *Fabp7* |
| ENSMUSG00000004730.16 | *Adgre1* |
| ENSMUSG00000004814.11 | *Ccl24* |
| ENSMUSG00000053063.12 | *Clec12a* |
| ENSMUSG00000044206.4 | *Vsig4* |
| ENSMUSG00000015854.8 | *Cd5l* |
| ENSMUSG00000014542.4 | *Clec4f* |
| ENSMUSG00000026390.8 | *Marco* |

**Supplementary references**

[1] Xia, X. Detailed dissection and critical evaluation of the Pfizer/BioNTech and Moderna mRNA vaccines. *Vaccines* **9**, 734 (2021).

[2] *World Health Organization*. *Messenger RNA encoding the full-length SARS-CoV-2 Spike Glycoprotein*,[https://web.archive.org/web/20210105162941/https://mednet-communities.net/inn/db/media/docs/11889.doc](https://web.archive.org/web/20210105162941/https:/mednet-communities.net/inn/db/media/docs/11889.doc) (2023).

[3] Fang, E., Liu, X., Li, M. *et al.* Advances in COVID-19 mRNA vaccine development. *Sig*. *Transduct*. *Target Ther*. **7**, 94 (2022)
